# Investigation of the impact of a broad range of temperatures on the physiological and transcriptional profiles of *Zymomonas mobilis* ZM4 for high-temperature-tolerant recombinant strain development

**DOI:** 10.1186/s13068-021-02000-1

**Published:** 2021-06-27

**Authors:** Runxia Li, Wei Shen, Yongfu Yang, Jun Du, Mian Li, Shihui Yang

**Affiliations:** 1 State Key Laboratory of Biocatalysis and Enzyme Engineering, Environmental Microbial Technology Center of Hubei Province, and School of Life Sciences, Hubei University, Wuhan, 430062 China; 2 Department of Biological and Chemical Engineering, Zhixing College of Hubei University, Wuhan, 430011 China; 3 China Biotech Fermentation Industry Association, Beijing, 100833 China; 4 Zhejiang Huakang Pharmaceutical Co., Ltd., Kaihua County, Zhejiang China

**Keywords:** *Zymomonas mobilis*, Temperature, Morphology, EGFP, RNA-Seq, Transcriptomics, Heat tolerance

## Abstract

The model ethanologenic bacterium *Zymomonas mobilis* has many advantages for diverse biochemical production. Although the impact of temperature especially high temperature on the growth and ethanol production of *Z. mobilis* has been reported, the transcriptional profiles of *Z. mobilis* grown at different temperatures have not been systematically investigated. In this study, *Z. mobilis* wild-type strain ZM4 was used to study the effect of a broad range of temperatures of 24, 30, 36, 40, and 45 °C on cell growth and morphology, glucose utilization and ethanol production, as well as the corresponding global gene expression profiles using RNA-Seq-based transcriptomics. In addition, a recombinant *Z. mobilis* strain expressing reporter gene *EGFP* (ZM4_EGFP) was constructed to study the effect of temperature on heterologous protein expression at different temperatures. Our result demonstrated that the effect of temperature on the growth and morphology of ZM4 and ZM4_EGFP were similar. The biomass of these two strains decreased along with the temperature increase, and an optimal temperature range is needed for efficient glucose utilization and ethanol production. Temperatures lower or higher than normal temperature investigated in this work was not favorable for the glucose utilization and ethanol production as well as the expression of exogenous protein EGFP based on the results of flow cytometry and Western blot. Temperature also affected the transcriptional profiles of *Z. mobilis* especially under high temperature. Compared with ZM4 cultured at 30 °C, 478 genes were up-regulated and 481 genes were down-regulated at 45 °C. The number of differentially expressed genes of ZM4 cultured at other temperatures (24, 36 or 40 °C) was relatively small though compared with those at 30 °C. Since temperature usually increases during the fermentation process, and heat tolerance is one of the important robustness traits of industrial strains, candidate genes related to heat resistance based on our RNA-Seq result and literature report were then selected for genetics study using the strategies of plasmid overexpression of candidate gene or replacement of the native promoter of candidate gene by an inducible P_*tet*_ promoter. The genetics studies indicated that *ZMO0236*, *ZMO1335*, *ZMO0994*, operon *groESL*, and *cspL,* which encodes Mrp family chromosome partitioning ATPase, flavoprotein WrbA, an uncharacterized protein, chaperonin Cpn10 and GroEL, and an exogenous cold shock protein, respectively, were associated with heat tolerance, and recombinant strains over-expressing these genes can improve their heat tolerance. Our work thus not only explored the effects of temperature on the expression of exogenous gene *EGFP* and endogenous genes, but also selected and confirmed several genes associated with heat tolerance in *Z. mobilis*, which provided a guidance on identifying candidate genes associated with phenotypic improvement through systems biology strategy and genetics studies for other microorganisms.

**Supplementary Information:**

The online version contains supplementary material available at 10.1186/s13068-021-02000-1.

## Background

Biofuel is an attractive substitute of fossil fuels to help address the severe problems of energy crisis and global warming [1, 2]. Yeast is the dominant microorganism for the first-generation bioethanol production. Another native ethanologen, *Zymomonas mobilis,* has the advantages as the biocatalyst for lignocellulosic biofuel production. *Z. mobilis* is a facultative anaerobic Gram-negative bacterium, producing ethanol through the unique anaerobic Entner–Doudoroff (ED) pathway [3, 4], which has the following advantages compared with yeast such as high sugar absorption efficiency, high ethanol yield, less biomass, no need to control oxygenation during fermentation, and relatively high tolerance against the inhibitory compounds in the lignocellulosic hydrolysates and the toxic end-products such as isobutanol [5–12].

High-temperature ethanol fermentation (HTEF) is an economic process for the large-scale bioethanol fermentation, which has advantages of reduced pollution risk and cooling costs, higher bioconversion rate, less energy cost for product recovery, and possibility for the more effective process of simultaneous saccharification and fermentation (SSF) than the classical batch fermentation [13, 14]. Based on their differences in growth, metabolism, and genotype, *Z. mobilis* was subdivided into three subspecies, which were *Z. mobilis* subsp*. mobilis*, *Z. mobilis* subsp*. pomaceae,* and *Z. mobilis* subsp*. Francensis*. The dramatic difference between *Z. mobilis* subsp*. mobilis* and other two subspecies is that *Z. mobilis* subsp*. mobilis* can grow above 36 °C, while *Z. mobilis* subsp*. pomaceae* and *Z. mobilis* subsp*. Francensis* cannot [15–17].

Temperature, pH, and oxygen level are the common physical factors that affect the growth and fermentation performance of biocatalysts. Previous studies indicated that temperature affected the growth and ethanol production of *Z. mobilis* with biomass and ethanol production reduced at higher temperatures [14, 18, 19]. Temperature also affected the morphology of *Z. mobilis*, and the cell shape changed from short rods under optimal temperature to elongated filaments under high temperature, which is similar to the morphology under other stress conditions such as wood hydrolysate, molasses, urea or various salts [20–22]. Stevnsborg et al. discovered that the shape of *Z. mobilis* cells returned to normal short rods from filaments when the culture temperature was reduced from 36 to 30 °C, and proposed that the arrest of septation and cell division was due to temperature-sensitive enzymes involved in septation [20]. Kosaka et al. further reported that cell elongation of *Z. mobilis* was repressed by Mg^2+^ at 39 °C [21]. Although the detailed mechanism behind this phenomenon is not clear, the activity of temperature-sensitive enzymes involved in septation may be stabilized by the Mg^2+^ at 39 °C [20].

Temperature not only affects cell morphology, but also changes cell membrane composition, fluidity, and leakage. Benschoter et al. showed that as the temperature increased, the components of saturated fatty acids, cardiolipin, and phosphatidylcholine increased correspondingly, which eventually reduced the membrane fluidity and increased the leakage of intracellular magnesium ions and proteins [23]. Temperature also affects the expression of heterologous proteins. For example, Vigants et al. found out that temperature was inversely proportional to the ratio of sucrose enzyme synthesis and enzymatic activity [24].

To develop robust strains for industrial applications, genes responsive for heat tolerance were identified through systems biology studies, and heat-resistant strains were developed through mutagenesis and metabolic engineering approaches. For example, Charoensuk et al. identified 26 genes related to heat tolerance in *Z. mobilis* through transposon mutagenesis, and classified these genes into nine categories of membrane stabilization, transporter, DNA repair, tRNA modification, protein quality control, translation control, cell division, and transcriptional regulation [25]. Samappito et al. obtained a mutant strain ZM AD41 through thermal adaptive evolution, the cell length of AD41 at high temperature was shorter than that of wild-type strain. In addition, AD41 grew faster and had better sedimentation performance than the wild-type strain at high temperature [26]. Kosaka et al. performed thermo-adaptive evolution of *Z. mobilis* TISTR548, CP4, and *Escherichia coli* K-12 strain W3110, which increased the critical high temperature (CHT) of these strains by 2–3 °C [27]. The cell length of the heat-adapted mutant strains of *Z. mobilis* TISTR548, CP4, and *E. coli* W3110 was significantly shorter than that of the corresponding wild-type strains or intermediate mutants under their CHTs [21]. They also studied the effects of different metal ions on the high-temperature tolerance of *Z. mobilis* TISTR548, and discovered that the addition of Mg^2+^ and K^+^ increased a CHT by 1 °C, reduced intracellular reactive oxygen species (ROS) level, and recovered ethanol productivity, but cell elongation was repressed by Mg^2+^, but not by K^+^ [21].

Although quite a few genes related to heat tolerance have been obtained, and the effects of temperature on cellular morphology and growth, sugar utilization and ethanol production as well as the expression of heterologous proteins of *Z. mobilis* have been investigated, nearly all of these works were focused on the performance of *Z. mobilis* under high temperature. There has no systemic research of *Z. mobilis* on a broad temperature range at transcriptional level to better understand the mechanism of heat resistance. In this work, the impact of temperature at a broad range of 24, 30, 36, 40 and 45 °C on cellular morphology and growth, glucose utilization and ethanol production, as well as the expression of the exogenous protein EGFP of recombinant strain ZM4_EGFP were studied, and gene expression profiles of *Z. mobilis* ZM4 under different temperatures were investigated using the RNA-Seq based transcriptomics, which help understand cellular responses to temperature systematically and provides gene targets for heat-tolerant recombinant strain development through genetics approaches.

## Results and discussion

### Impact of temperature on heterologous gene expression and physiological responses of *Z. mobilis*

**Fig. 1:**
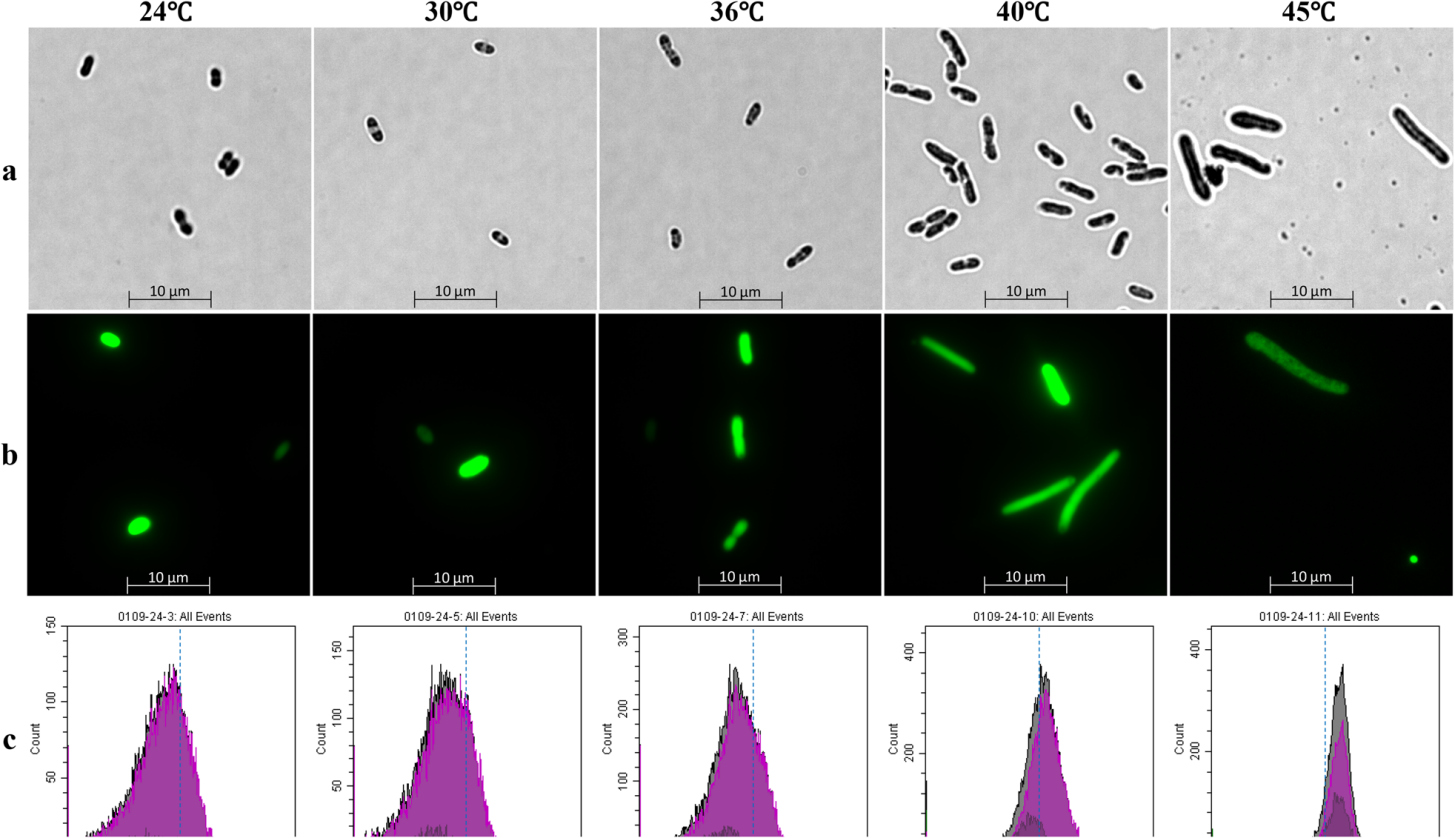
Microscopic observation of ZM4 (**a**) and ZM4_EGFP (**b**) as well as flow cytometry results of ZM4_EGFP (**c**) grown at different temperatures of 24, 30, 36, 40, and 45 °C. FSC is a forward scattering angle, generally representative of the volume of the cell

**Fig. 2:**
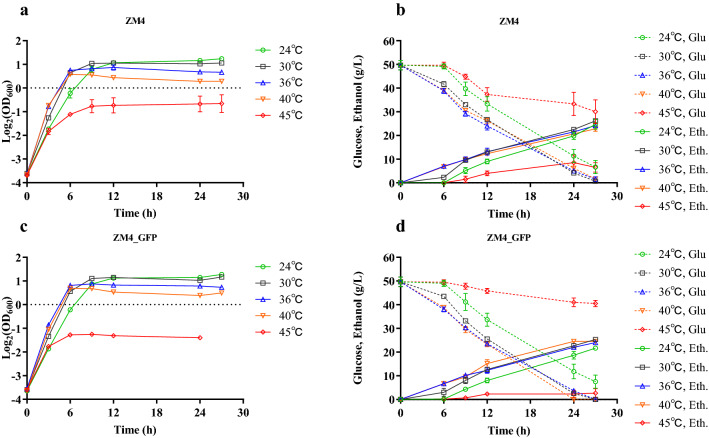
Cell growth (**a**), glucose (Glu) consumption and ethanol (Eth) production (**b**) of ZM4, as well as cell growth (**c**), glucose (Glu) consumption and ethanol (Eth) production (**d**) of ZM4_GFP in rich medium (RM) at different temperatures of 24, 30, 36, 40 and 45 °C

To explore the impact of temperature on cell growth and the expression of heterologous protein in *Z. mobilis*, a recombinant strain ZM4_EGFP carrying the shuttle plasmid fused with reporter gene *EGFP* [28–30] encoding green fluorescent protein (pEZ_EGFP) was constructed. Our result indicated that the expression of heterologous gene *EGFP* had minimal impact on cell morphology, growth, glucose utilization and ethanol production compared with the wild-type strain ZM4, while temperature had a great influence on these two strains (Figs. 1, 2).

The microscopic and flow cytometry results of ZM4 and ZM4_EGFP grown at different temperatures are shown in Fig. 1. Within the temperature range of 24–45 °C, cells gradually changed from a short rod shape to a filament shape as the temperature increased (Fig. 1a, b), which was consistent with previous studies [20, 27]. At the same time, the results of the flow cytometer were also consistent with those of the microscopic ones (Fig. 1). As the temperature increased, the peak value of FSC that was positively correlated with the cell size gradually increased from less than 10^4^ to greater than 10^4^ (Fig. 1c).

Cell biomass of the strain, however, decreased along with the increase of temperatures. Compared with the time for ZM4 and ZM4_EGFP to reach stationary phase at normal temperature of 30 °C, the time for ZM4 and ZM4_EGFP grown at low (24 °C) temperature was longer, while the time for ZM4 and ZM4_EGFP grown at 36 °C and 40 °C was shorter (Fig. 2). In the range of 24 and 36 °C, the specific growth rate (*µ*) of the two strains increased with the increase of temperature. The specific growth rates of these two strains grown at 36 °C and 40 °C were similar while the specific growth rates of two strains grown at 45 °C were the lowest (Fig. 2 and Additional file 1: Table S1). At high temperatures especially at 45 °C, the ethanol productivity (Qp) of ZM4 decreased significantly compared with normal temperature of 30 °C (Fig. 2 and Additional file 1: Table S1).

In short, the optimum growth temperature of ZM4 and ZM4_GFP was 30 °C with the highest biomass within short period of growth time (Fig. 2a, c), and the specific growth rate of both ZM4 and ZM4_GFP increased when temperature increased from 24 to 40 °C (Fig. 2 and Additional file 1: Table S1). However, both cell growth and sugar to ethanol conversion were severely inhibited when the temperature reached 45 °C, and the overexpression of heterologous protein such as EGFP in this study affected cell growth, glucose consumption, and ethanol production at high temperature of 45 °C (Fig. 2). Compared with the highest OD_600_ value around 0.70 for ZM4 strain at 45 °C, the highest OD_600_ value of ZM4_GFP strain at 45 °C was only about 0.40. Correspondingly, the highest ethanol that ZM4 produced was 8.5 ± 3.3 g with 19.7 ± 6.8 g glucose consumed while the highest ethanol that ZM4_GFP can produced was only 3.8 ± 0.4 g with only 9.2 ± 2.8 g glucose consumed within 27 h post-inoculation (Fig. 2 and Additional file 1: Table S1).

### The effect of temperature on the expression of heterologous gene in *Z. mobilis*

**Fig. 3:**
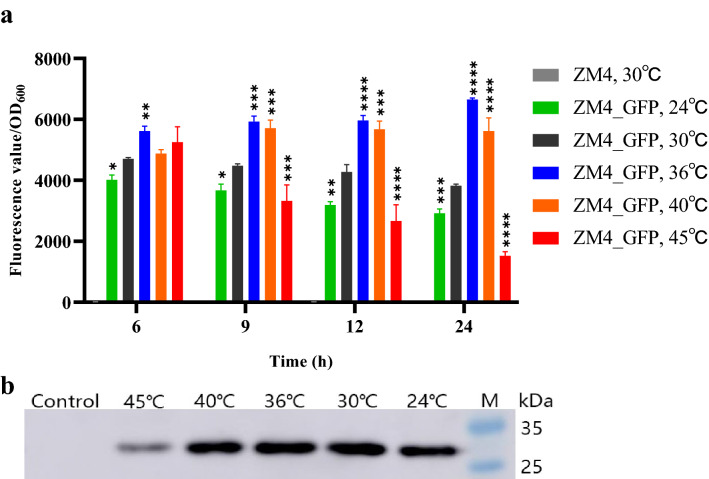
The ratios of fluorescence value/OD_600_ of recombinant strain ZM4_EGFP at different temperatures of 24, 30, 36, 40, and 45 °C using a microplate reader (**a**), and Western blot result of EGFP in ZM4_EGFP cultured at different temperatures of 24, 30, 36, 40, and 45 °C (**b**). “ZM4, 30 °C” indicated the control condition of wild-type strain ZM4 grown at 30 °C. Control represented wild-type strain ZM4 cultured at 30 °C. M was Pre-dyed SDS-PAGE Standard. One-way ANOVA of the ratios of fluorescence intensity value and OD_600_ of ZM4_GFP at 24, 36, 40, and 45 °C were conducted with 30 °C as a control at 6, 9, 12, and 24 h, respectively, * represents a significant difference (0.01 < *P*-value < 0.05), ** represents a very significant difference (*P*-value < 0.01), *** represents *P*-value < 0.001, **** represents *P*-value < 0.0001

The introduction of exogenous proteins and metabolic pathways can not only broaden the substrate spectrum of *Z. mobilis*, but also provide the possibility for diverse biochemical production in *Z. mobilis*. Since temperature is one of the important elements that affect the expression and activity of proteins, and there are relatively few reports about the influence of the temperature on heterologous gene expression in *Z. mobilis*, a recombinant *Z. mobilis* strain expressing reporter gene *EGFP* (ZM4_EGFP) was constructed in this work to study the effects of temperature on the growth and fermentation performance of ZM4_GFP at different temperatures as discussed above (Fig. 2c, d). We further investigated the impact of different temperatures on the expression of heterologous gene such as *EGFP* in ZM4_EGFP using SpectraMax M2e Microplate Reader (Fig. 3a), flow cytometer (data not shown), and Western blot (Fig. 3b), which can be used as a reference for the efforts to express other exogenous genes and to construct metabolic pathways in ZM4 under different temperatures in the future.

Within 12 h, the ratios of fluorescence value/OD_600_ increased significantly over time at 30, 36, and 40 °C, but not at the lowest and the highest temperatures of 24 and 45 °C. The ratios of fluorescence value/OD_600_ were relatively high at 30, 36, 40 °C with the highest ratio of fluorescence/OD_600_ at 36 °C (Fig. 3a). The results of flow cytometer were consistent with the results using a microplate reader (Fig. 3a), both showing that the fluorescence value of individual cell was higher at 36 °C and 40 °C, which indicated that higher temperatures may be beneficial to the expression of the heterologous EGFP protein with high fluorescence intensity.

The results of Western blot experiments also exhibited that the expression of EGFP protein in the recombinant strain ZM4_EGFP was strong at 30, 36, and 40 °C, while the expression of EGFP protein decreased at 24 °C and especially at 45 °C (Fig. 3b). Similar to the results of Fig. 3a, high temperature (45 °C) was disadvantageous for heterologous EGFP protein expression or caused protein degradation due to cell lysis (Fig. 3b).

### Effects of temperatures on global transcriptional profiles of *Z. mobilis*

**Fig. 4:**
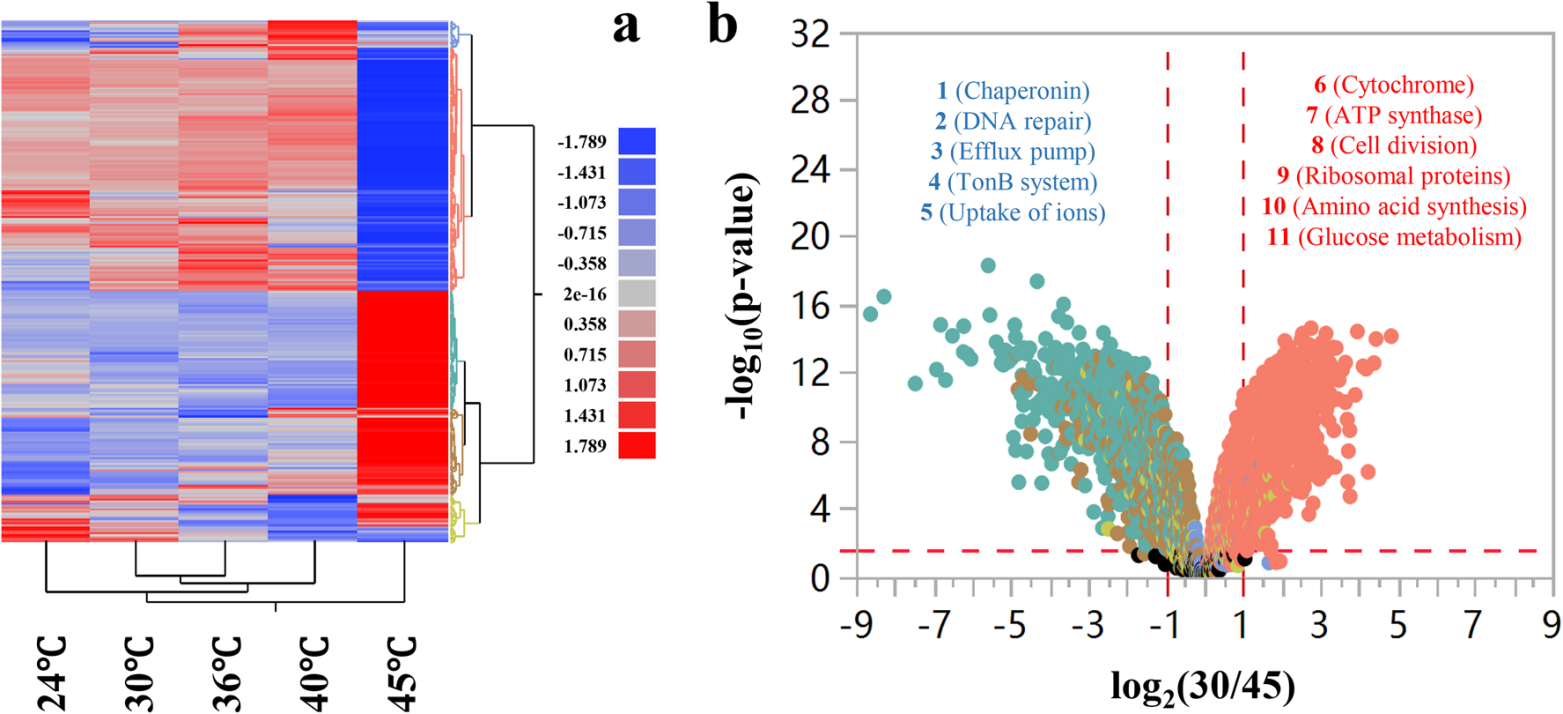
Heatmap analysis of ZM4 transcriptional profiles at different temperatures of 24, 30, 36, 40, and 45 °C (**a**), and Volcano plots of significantly differentially expressed genes of *Z. mobilis* cultured at 30 °C, compared with 45 °C (30/45) (**b**). In **b**, the dots above the horizontal red dash line indicate genes significantly differentially expressed, and the vertical red dash line indicate genes significantly differentially expressed with ratio greater than 2 (log_2_-based ratio greater than 1). Gene name with red and blue color font indicates up-regulated and down-regulated genes of ZM4 cultured at 30 °C compared with 45 °C, respectively. The numbers 1–5 referred to genes related to chaperonin, DNA repair, efflux pump, TonB system, and ions uptake, respectively. The numbers 6–11 referred to genes related to cytochrome, ATP synthesis, cell division, ribosomal proteins, amino acid synthesis, and glucose metabolism, respectively

The effect of temperature on the transcriptional level of the entire genome was investigated by RNA-Seq transcriptomics to explore genes responsive for temperature changes. Genes that were differentially expressed in response to temperature raise and those at high temperature can be used to identify gene targets for heat-tolerant strain construction. The RNA-seq results obtained by culturing ZM4 at different temperatures showed that gene expression fluctuates significantly at 45 °C (Fig. 4).

Among significantly differentially expressed genes, there were 478 genes up-regulated and 481 genes down-regulated in ZM4 cultured at 45 °C compared with 30 °C, which showed that the temperature had a significant effect on transcriptomic profiles, especially at 45 °C (Fig. 4a). Compared with normal temperature 30 °C, ZM4 had fewer differentially expressed genes at low temperature (24 °C) and other temperatures examined in this study (36 °C, 40 °C). In terms of temperature increase (24/30, 30/36, 36/40, 40/45), when the culture temperature did not exceed 40 °C, the number of differentially expressed genes was less than a hundred, but it can be clearly seen that compared with 40 °C, the number of differentially expressed genes in ZM4 exceeded 1000 under high temperature of 45 °C (Table 1). Compared with those at 30 °C, 959 genes including those encoding chaperones, cell division proteins, out membrane transporters were significantly differentially expressed at 45 °C, accounting for more than half of the total genes of ZM4 (Fig. 4b and Additional file 2: Table S2).

**Table 1:** Number of significantly differentially expressed genes at different temperatures, the numbers in the brackets are the numbers of up-regulated and down-regulated genes, respectively

Comparisons	Number of differentially expressed genes (up-regulated, down-regulated)	Subtab number in Additional file 2: Table S2 for detailed information
30/45	959 (481, 478)	Table S2_a
30/40	81 (54, 27)	Table S2_b
30/36	21 (20, 1)	Table S2_c
24/30	12 (12, 0)	Table S2_d
40/45	1014 (527, 487)	Table S2_e
36/40	62 (23, 39)	Table S2_f

For example, “30/45” referred to differentially expressed genes between ZM4 cultured at 30 °C and 45 °C. Differentially expressed genes at different temperatures are listed in Additional file 2: Table S2

Compared with normal temperature of 30 °C, the genes encoding chaperone proteins (DnaK, DnaJ, ClpB, Cpn10, and GroEL), TonB-related proteins (ZMO0789, ZMO0902, ZMO1040, ZMO1463, ZMO1522, ZMO0561, ZMO1298, ZMO1986, ZMO1631, ZMO1815, and ZMO1822), DNA repair-related proteins (RecA, RedC, RecR, RecF, MutL, and MutS), and efflux pump-related proteins (ZMO0965, ZMO0282, ZMO0283, and ZMO0287) were up-regulated in ZM4 cultured at the high temperature of 45 °C. It was reported that the upregulation of genes encoding chaperone proteins can improve the stress resistance of cells [31]. The overexpression of chaperone proteins under high temperature may be one of the strategies that cells imply to deal with the damages caused by heat stress.

Interestingly, more than 70% of TonB-related proteins in ZM4 were up-regulated at 45 °C. More than two-thirds of Gram-negative bacteria possessed the TonB system, which plays an important role in the intake of various nutrients such as vitamin B_12_, carbohydrates, iron and zinc ions [32–36]. Meanwhile, in the transcriptome data, genes related to the uptake of iron ions (*ZMO0423*, *ZMO0428*, *ZMO0429*, and *ZMO1596*) and zinc ions (*ZMO1236* and *ZMO1341*) were up-regulated at high temperature. These results suggested that cells may up-regulate genes associated with TonB system and transporters of important nutrients to maintain normal cell metabolism by promoting the uptake of nutrients and cofactors under the high temperature stress condition.

As a cofactor of many enzymes and necessary for the stability of zinc finger proteins, zinc was associated with cellular metabolism including stress responses and biosynthesis of plasma membrane, such as biosynthesis of phospholipid in *Saccharomyces cerevisiae* and biosynthesis of ergosterol and trehalose in self-flocculating yeast [37–39]. The supplementation of zinc significantly improved the ethanol and heat tolerance of self-flocculating yeast, which was closely related to the increased ergosterol and trehalose contents in the yeast flocs [37]. The upregulation of genes related to the uptakes of iron and zinc in this study suggested that a similar role of zinc may also exist in *Z. mobilis* to help stabilize the structure of cell membranes and enzyme activities in response to heat and ethanol stresses.

Genes down-regulated under high temperature compared with normal temperature mainly involved in energy metabolism and protein biosynthesis such as ATP synthase-related genes (*ZMO1686*, *ZMO0667*, *ZMO0239*, *ZMO0669*, *ZMO0671*, *ZMO0241*, *ZMO0668*, *ZMO0242*, *ZMO0240*, and *ZMO0238*), cytochrome-related genes (*ZMO0957*, *ZMO1571*, *ZMO1258*, *ZMO1255*, *ZMO1253*, *ZMO1572*, and *ZMO0806*), glucose metabolism-related genes (*ZMO1757*, *ZMO1756*, *ZMO1649*, *ZMO0366*, *ZMO1981*, and *ZMO0689*), glutamate, histidine, and cysteine synthesis-related genes (*ZMO1117*, *ZMO0457*, *ZMO1964*, *ZMO0783*, *ZMO0782*, *ZMO0784*; *ZMO0480*, *ZMO1105*, *ZMO1962*, *ZMO0752*, *ZMO1508*; *ZMO0005*, *ZMO0007*, and *ZMO0008*), as well as ribosome protein-related genes (*ZMO0884*, *ZMO0532*, *ZMO1079*, *ZMO0534*, and *ZMO1145*). The down-regulation of genes related to glucose metabolism at high temperature was consistent with the decrease of glucose consumption in ZM4 at high temperature (Fig. 2). The down-regulation of genes related to cellular respiratory chain and protein biosynthesis could be a strategy for cell survival by reducing energy consumption in response to extreme environments, and the down-regulation of genes related to cell division (*ZMO0830* and *ZMO0835*) under high temperature may lead to a decrease in the frequency of cell division and thus the phenomenon of cell lengthening (Fig. 1).

**Fig. 5:**
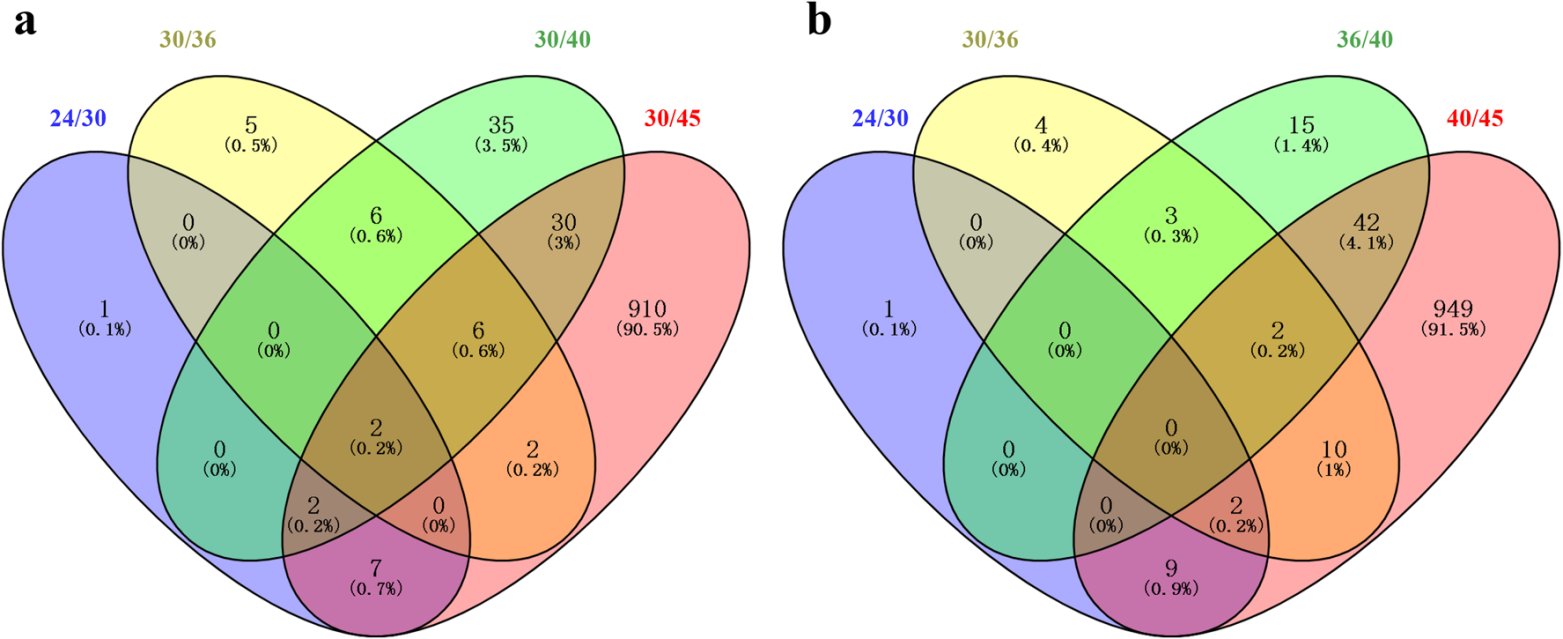
Venn diagram of differential expressed genes of ZM4 cultured at 24 °C compared with 30 °C (24/30), ZM4 cultured at 30 °C compared with 36 °C (30/36), ZM4 cultured at 30 °C compared with 40 °C (30/40), and ZM4 cultured at 30 °C compared with 45 °C (30/45) (**a**). Venn diagram of differential expressed genes with the raise of temperature gradually for ZM4 cultured at 24 °C compared with 30 °C (24/30), ZM4 cultured at 30 °C compared with 36 °C (30/36), ZM4 cultured at 36 °C compared with 40 °C (36/40), and ZM4 cultured at 40 °C compared with 45 °C (40/45) (**b**)

We further drew two Venn diagrams for four datasets of differentially expressed genes of ZM4 cultured at different temperatures of 24, 36, 40, and 45 °C compared with 30 °C (Fig. 5a), and four datasets of differentially expressed genes of ZM4 with the increase of temperatures from 24 to 45 °C gradually (Fig. 5b) to identify common genes that are potentially responsive to temperature changes. Our results indicated that the differences between differentially expressed genes due to temperature raise from 24 to 45 °C or different temperature comparisons with 30 °C were similar (Fig. 5). There were too many differentially expressed genes for ZM4 cultured at 45 °C compared 30 °C as well as 45 °C compared with 40 °C, and high temperature of 45 °C had great impact on global gene expression.

Forty-nine genes in the region that overlaps with the differentially expressed genes of ZM4 cultured at 45 °C compared with 30 °C (“30/45”) with those from the comparisons of “24/30”, “30/36”, and “30/40” were likely to respond to heat stress (Fig. 5a and Table 2). Similarly, genes related to chaperonin and DNA repair were mainly up-regulated at high temperature, while genes related to glucose metabolism and amino acid synthesis were down-regulated at high temperature. Eleven genes shown in bold font in Table 2 were up-regulated in both high (45 °C) and low temperature (24 °C) conditions than at normal temperatures (30 °C) (Additional file 3: Table S3). These temperature-responsive genes could serve as temperature control switches to the changing temperatures.

**Table 2:** List of 49 significantly differentially expressed genes between ZM4 cultured at 30 °C and 45 °C

Gene ID	Product	Ratio	− log_10_ (*P*-value)
Upregulated gene in ZM4 cultured at 30 °C compared with 45 °C			
* ZMO0005*	Sulfate adenylyltransferase small subunit	1.35	3.24
* ZMO0008*	Sulfite reductase hemoprotein beta-component	1.31	2.59
* ZMO0374*	Levansucrase	1.83	5.90
* ZMO0375*	Levansucrase/invertase	1.64	5.43
* ZMO0379*	PBSX family phage terminase large subunit	1.53	5.18
* ZMO0395*	Hypothetical protein	2.20	5.52
* ZMO0397*	Hypothetical protein	1.84	4.79
* ZMO0454*	Formate-tetrahydrofolate ligase	2.39	10.71
* ZMO0492*	Nitrogen regulatory protein PII	3.16	11.78
* ZMO1117*	Glutamate synthase large subunit	1.44	7.99
* ZMO1719*	Fructokinase	1.73	10.52
* ZMO1748*	ArsR family transcriptional regulator	1.36	5.73
* ZMOp36x011*	Tail sheath protein	1.82	4.51
* ZMOp36x012*	Tail tube protein	1.60	4.35
Downregulated gene in ZM4 cultured at 30 °C compared with 45 °C			
*** ZMO0122***	**Uncharacterized protein**	− **2.86**	**9.99**
*** ZMO0286***	**DUF541 domain-containing protein**	− **2.56**	**9.78**
*** ZMO0693***	**OsmC family protein**	− **1.57**	**8.5**
*** ZMO0740***	**General stress protein CsbD**	− **3.06**	**8.03**
*** ZMO1113***	**FAD-dependent pyridine nucleotide-disulfide oxidoreductase**	− **1.62**	**8.34**
*** ZMO1237***	**Lactate dehydrogenase**	− **2.82**	**9.39**
*** ZMO1522***	**TonB-dependent receptor**	− **1.01**	**3.88**
*** ZMO1533***	**Hypothetical protein**	− **3.01**	**12.07**
*** ZMO1754***	**Succinate-semialdehyde dehydrogenase SSADH**	− **3.95**	**11.33**
*** ZMO1776***	**Aminopeptidase N**	− **1.97**	**9.64**
*** ZMO1940***	**Hypothetical protein**	− **4.87**	**7.45**
* ZMO0003*	Adenylyl-sulfate kinase	− 1.23	2.12
* ZMO0246*	ATP-dependent protease subunit HslV	− 2.06	11.16
* ZMO0400*	Hypothetical protein	− 1.17	5.94
* ZMO0422*	BadM/Rrf2 family transcriptional regulator	− 1.25	7.82
* ZMO0660*	Chaperone protein DnaK	− 3.52	10.65
* ZMO0748*	Cysteine synthase	− 3.3	9.68
* ZMO0989*	Heat-shock protein IbpA	− 8.63	15.44
* ZMO1062*	Putative phage shock protein pspD	− 2.33	8.1
* ZMO1063*	Phage shock protein A PspA	− 2.9	10.94
* ZMO1271*	Siroheme synthase CysG	− 4.29	11.34
* ZMO1424*	ATP-dependent chaperone ClpB	− 1.82	11.22
* ZMO1426*	DNA repair protein RadC	− 1.22	6.63
* ZMO1463*	TonB-dependent receptor	− 1.41	4.44
* ZMO1586*	Bacterioferritin	− 1.46	5.72
* ZMO1721*	Glyoxalase/bleomycin resistance protein dioxygenase	− 4.59	13.11
* ZMO1849*	Uncharacterized protein	− 3.55	8.15
* ZMO1928*	Chaperonin Cpn10	− 6.22	13.21
* ZMO1929*	Chaperonin GroEL	− 6.03	12.81
* ZMOp33x029*	Azospirillum phage Cd Gp10 family protein	− 2.4	7.07
* ZMOp33x030*	Hypothetical protein	− 2.45	8.57
* ZMOp39x009*	Putative partitioning protein ParA ATPase	− 1.39	8.45
* ZMOp39x010*	Putative partitioning protein ParB	− 1.23	6.27
* ZMOp39x036*	Hypothetical protein	− 3.79	12.36
* ZMOp39x037*	Hypothetical protein	− 4.21	11.34

Ratio is the log_2_-based expression difference between ZM4 cultured at 30 °C and 45 °C. Gene name with red and blue color font indicates up-regulated and down-regulated at 30 °C compared with 45 °C, respectively. Eleven genes shown in bold font were up-regulated in both high (45 °C) and low temperature (24 °C) conditions than at normal temperatures (30 °C) (Additional file 3: Table S3)

### Selection and characterization of heat-tolerant candidate genes

Among three subspecies of *Z. mobilis*, *Z. mobilis* subsp*. mobilis* is heat resistant, while *Z. mobilis* subsp*. pomaceae* and *Z. mobilis* subsp*. francensis* are not [15]. The complete protein sequences of ZM4 were compared with those of ATCC 29192 that is belonging to subsp*. pomaceae* with 12 genes unique to ZM4 identified, which could be potentially heat-tolerant genes in ZM4. The expression values of these genes are listed in Table 3.

**Table 3:** Unique genes of ZM4 compared with ATCC 29192 and their expression values (log_2_RPKM) at different temperatures

Gene ID	Product	log_2_RPKM				
		24 °C	30 °C	36 °C	40 °C	45 °C
*ZMO1465*	Hypothetical protein	8.09	8.38	8.40	7.95	6.25
*ZMO1483*	Hypothetical protein	2.07	2.43	2.30	3.15	3.52
*ZMO1628*	PepSY-associated TM helix domain protein	5.24	5.20	5.45	5.45	5.77
*ZMO2037*	Hypothetical protein	3.31	2.51	2.41	3.03	7.63
*pZYM32_014*	Hypothetical protein	7.31	7.43	6.93	8.02	10.71
*pZYM32_016*	Hypothetical protein	0.50	0.25	0.00	0.00	0.00
*pZYM32_028*	Hypothetical protein	6.98	7.03	7.05	7.51	6.47
*pZYM36_005*	Phage morphogenesis protein	4.82	5.05	4.50	5.19	7.01
*pZYM36_026*	Hypothetical protein	6.24	6.23	5.98	5.61	5.93
*pZYM36_030*	Hypothetical protein	9.22	9.47	8.96	8.94	10.93
*pZYM36_037*	Hypothetical protein	6.66	6.59	6.29	6.31	6.58
*pZYM36_045*	Small terminase subunit	4.80	4.54	4.06	5.32	5.66

Through transposon mutagenesis, Charoensuk et al. found 26 genes related to heat tolerance in *Z. mobilis* TISTR 548 (ATCC 29191) [25]. The protein sequences of these genes were also compared with complete ZM4 protein sequences to identify their homologous proteins in ZM4, and the expression values of genes in ZM4 with homologues in *Z. mobilis* TISTR 548 that were reported to be associated with heat tolerance at different temperatures are summarized in Table 4.

**Table 4:** The expression values (log_2_RPKM) of genes in ZM4 corresponding to 26 genes related to heat tolerance of *Z. mobilis* TISTR 548 (ATCC 29191) at different temperatures

ATCC29191 Gene ID	Product	ZM4 Gene ID	log_2_RPKM				
			24 °C	30 °C	36 °C	40 °C	45 °C
*ZZ6_0707*	Glucose sorbosone dehydrogenase	ZMO0558	7.20	7.16	7.09	7.15	7.22
*ZZ6_1376*	5,10-methylenetetrahydrofolate reductase	ZMO1747	6.39	6.57	7.20	7.87	6.08**
*ZZ6_1146*	Glucosamine-fructose-6-phosphate aminotransferase	ZMO0056	7.66	7.53	7.53	7.48	8.08*
*ZZ6_0929*	Glycosyl transferase group	ZMO0306	6.62	6.36	6.40	6.30	5.73*
*ZZ6_0923*	Phospholipase D/transphosphatidylase	ZMO0314	6.41	6.42	6.47	6.50	6.74*
*ZZ6_1551*	Squalene-hopene cyclase	ZMO1548	7.19	7.25	7.34	7.45	7.98**
*ZZ6_1046*	MotA/TolQ/ExbB proton channel	ZMO0161	7.93	8.12	7.99	7.69	6.75**
*ZZ6_1043*	Protein TolB	ZMO0165	8.56	8.68	8.45	8.09	7.91**
*ZZ6_1254*	Protein-export protein	ZMO1897	8.88	9.14	9.24	9.13	7.49**
*ZZ6_1477*	Import inner membrane	ZMO1636	9.27	9.34	9.12	9.29	10.89****
*ZZ6_0158*	Protein of unknown function	ZMO1173	6.85	7.04	7.19	7.33	6.78*
*ZZ6_1210*	ComEC/Rec2-related protein	ZMO1965	4.84	5.01	5.17	5.18	5.52**
*ZZ6_0840*	MJ0042 family finger-like	ZMO0414	8.30	8.36	8.35	8.26	7.00***
*ZZ6_0541*	Protein of unknown function	ZMO0746	7.47	7.68	7.70	7.89	7.56
*ZZ6_1289*	Protein of unknown function	ZMO1860	7.12	7.14	7.06	7.02	6.27***
*ZZ6_0616*	DNA repair protein	ZMO0663	5.67	5.66	5.63	5.82	5.67
*ZZ6_0934*	Exodeoxyribonuclease 7 large	ZMO0300	6.50	6.68	6.71	6.82	7.39**
*ZZ6_0681*	DNA repair protein	ZMO0589	6.70	6.81	6.68	6.68	6.26*
*ZZ6_0023*	tRNA/rRNA methyltransferase	ZMO1328	7.85	8.08	8.21	8.19	7.19*
*ZZ6_1659*	Peptidase M16 domain	ZMO1422	7.74	7.46	7.23	7.20	6.67**
*ZZ6_0980*	Protease Do	ZMO0234	8.97	8.74	8.56	8.57	10.08****
*ZZ6_0702*	ATP-dependent helicase HrpB	ZMO0565	6.20	6.50	6.69	6.87	6.81*
*ZZ6_0979*	ATPase-like, ParA/MinD	ZMO0236	7.06	7.20	7.13	7.06	8.98***
*ZZ6_0019*	Flavoprotein WrbA	ZMO1335	7.56	7.08	7.07	7.33	9.44****
*ZZ6_0962*	Pseudogene	No homologue identified					
*ZZ6_0861*	Hypothetical protein	ZMO0391	4.23	4.93	4.24	2.27	5.70

*T*-test of ZM4 cultured at 45 °C were conducted with 30 °C as a control, * represents a significant difference (0.01 < *P*-value < 0.05), ** represents a very significant difference (*P*-value < 0.01), *** represents *P*-value < 0.001, **** represents *P*-value < 0.0001

At 45 °C, the expression of *ZMO1636* (membrane stability), *ZMO0234* (protein quality control), *ZMO0236* (cell division), and *ZMO1335* (transcription control) were significantly increased in ZM4 (Table 4). There are about 500 bp DNA fragment between *ZMO0234* and *ZMO0236*, which could be a bi-directional promoter, driving the transcription of both *ZMO0234* and *ZMO0236*. Considering the expression levels of *ZMO0234* and *ZMO0236* increased significantly at 45 °C (Table 4), it is possible that regulatory elements related to thermal stability exist within the 500 bp DNA fragment between these two genes.

The constitutive overexpression of GroEL, GroES, and ClpB in *Pseudomonas putida* KT2440 can improve its tolerance to a variety of thermochemical wastewater samples [31]. The first two proteins can form a GroESL complex for protein folding. The function of ClpB is to depolymerize the protein, by combining with DnaJKE and/or GroESL complexes to break down and refold the protein aggregates into functional proteins. In addition, overexpression of *ZMO0994* gene of *Z. mobilis* in *E. coli* DE3 improved the tolerance of DE3 to abiotic stresses such as ethanol, furfural, hydroxymethyl furfural, and heat [26], indicating that *ZMO0994* is associated with the resistance to abiotic stress. However, this impact of overexpression of ZMO0994 gene on heat tolerance in *Z. mobilis* has not been explored yet. In addition to the chaperones discussed above, cold shock protein CspL from the thermophilic bacterium *Bacillus coagulans* 2–6 was systematically investigated recently, and the results demonstrated that CspL plays a role in heat tolerance, which can promote the growth of diverse microorganisms under heat stress including *E. coli* DH5α, *S. cerevisiae* INVSc1, and *P. putida* KT2440 through binding diverse RNA species at high temperatures [40].

Based on the results in Tables 3 and 4, the following genes were selected for recombinant strain construction to verify whether these genes are related to heat resistance in *Z. mobilis*. ZM4_014, ZM4_005, ZM4_028, ZM4_1465, ZM4_1483, ZM4_1628, ZM4_2037, ZM4_0234, ZM4_0236, ZM4_1335, ZM4_1636, ZM4_0015 referred to recombinant strains over-expressing genes of *pZYM32_014* (an operon including genes *pZYM32_012-015*), *pZYM36_005*, *pZYM32_028*, *ZMO1465*, *ZMO1483*, *ZMO1628*, *ZMO2037*, *ZMO0234*, *ZMO0236*, *ZMO1335*, *ZMO1636*, and *ZMO0015* by homologous recombination to replace the native promoters of these genes with a tetracycline inducible promoter P_*tet*_, respectively. In addition, genes encoding the chaperone proteins, an uncharacterized protein, and an exogenous cold shock protein CspL were overexpressed using the shuttle plasmid pEZ15Asp [41] to obtain recombinant strains ZM4 (pEZ_clpB), ZM4 (pEZ_clpB_groESL), ZM4 (pEZ_groESL), ZM4 (pEZ_0994), and ZM4 (pEZ_cspL), respectively.

**Fig. 6:**
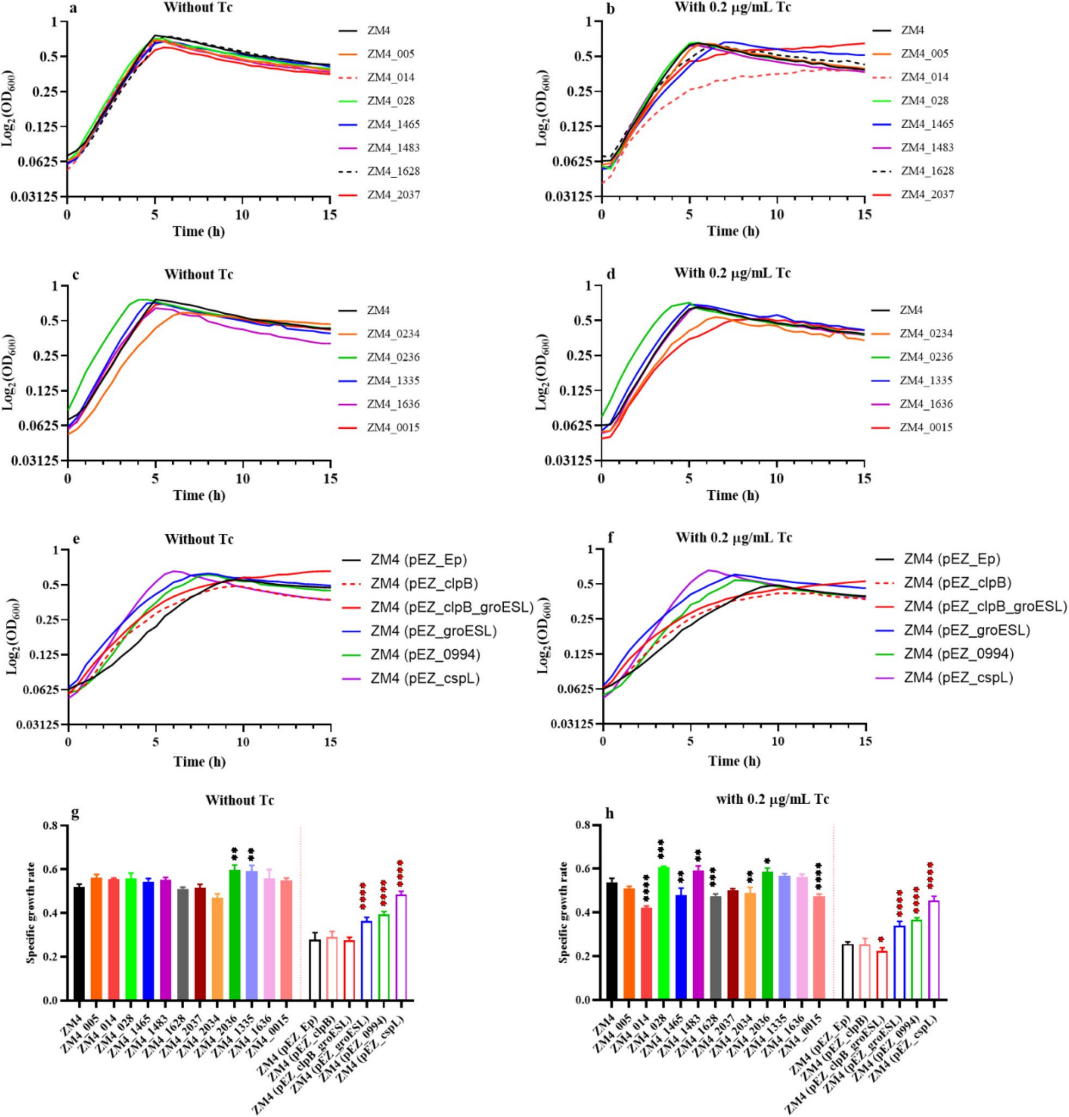
Growth curves and specific growth rates of mutants at 40 °C with the concentration of tetracycline at 0 or 0.2 µg/mL. The tetracycline concentration in the culture medium of figures **a**, **c**, **e** and **g** was 0 µg/mL, while the tetracycline concentration in the culture medium of figures **b**, **d**, **f** and **h** was 0.2 µg/mL. The media in figures **a**–**d** and the left of the red dashed line in figure** g** and **h** were RM, while the media in figures **e**, **f** and the right of the red dashed line in figure** g** and **h** were RM supplemented with 50 µg/mL spectinomycin. ZM4 was wild-type strain used as the control for mutants with the native promoter replaced by an inducible promoter P_*tet*_. ZM4 (pEZ_Ep) was ZM4 strain containing a shuttle vector pEZ15Asp as the control for recombinant strains over-expressing candidate genes. ZM4_XXX referred to mutants with native promoters replaced by an inducible promoter P_*tet*_ (for example, ZM4_005 and ZM4_1465 referred to gene *pZYM36_00*5 and *ZMO1465* with their native promoters replaced by the inducible promoter P_*tet*_, respectively.). ZM4 (pEZ_XXX) referred to recombinant strains over-expressing candidate genes. For example, ZM4 (pEZ_clpB) referred to ZM4 with an extra copy of *clpB* driving by P_*tet*_ on the shuttle vector pEZ15Asp except that *groESL* in ZM4 (pEZ_groESL) was driven by its native promoter. One-way ANOVA of specific growth rate of mutants at 40 °C were conducted with ZM4 or ZM4 (pEZ_Ep) as the control, * represents a significant difference (0.01 < *P*-value < 0.05), ** represents a very significant difference (*P*-value < 0.01), *** represents *P*-value < 0.001, **** represents *P*-value < 0.0001

The results showed that ZM4_0236 and ZM4_1335 grew better than the wild-type ZM4 under 0 µg/mL tetracycline induction (Fig. 6c, g), indicating that the overexpression of above two genes is beneficial to improve the growth of ZM4 at high temperature. It needs to be pointed out that when the tetracycline concentration was 0 µg/mL, the P_*tet*_ promoter is still functional due to the leaky background expression of P_*tet*_ promoter. However, ZM4_014 or ZM4_0015 grew significantly worse when operon *pZYM32_012*-*015* or gene *ZMO0015* was overexpressed with 0.2 µg/mL tetracycline induction compared with no tetracycline induction (Fig. 6a–d, g, h). *ZMO0015* encodes the heat-induced transcriptional repressor HcrA, which is a negative regulator of heat shock-like genes (*grpE*-*dnaK*-*dnaJ* and *groELS* operons) and prevents heat shock induction of these heat shock genes. ZM4_014 was obtained by replacing the promoter of *pZYM32_012*-*015* operon encoding several hypothetical proteins. Under 0.2 µg/mL tetracycline induction conditions, the tolerance of ZM4_014 and ZM4_0015 to high temperature was significantly reduced, which indicated that overexpression of these genes resulted in heat sensitive instead of heat tolerance.

For mutants over-expressing candidate genes using shuttle vector pEZ15Asp, recombinant strains of ZM4 (pEZ-0994) and ZM4 (pEZ_cspL) grew significantly better than the control strain ZM4 (pEZ_Ep) (Fig. 6e–h), which was consistent with previous reports that the overexpression of gene *ZMO0994* and heterologous gene *cspL* can help promote cellular growth significantly at a high temperature [26, 40]. In addition, when only *groESL* operon was overexpressed, the specific growth rate of recombinant strain ZM4 (pEZ_groESL) was significantly higher than that of ZM4 (pEZ_Ep) at high temperatures (Fig. 6e–h), which was also consistent with previous report in *P. putida* [31]. However, when *groESL* was overexpressed together with *clpB* or *clpB* was overexpressed alone, both recombinant strains of ZM4 (pEZ_clpB_groESL) and ZM4 (pEZ_clpB) grew worse than the control strain ZM4 (pEZ_Ep) at the concentration of 0 and 0.2 ug/mL tetracycline (Fig. 6e, f). Further study is needed to help understand the role of *clpB* and the effect of different combinations of chaperone genes on heat tolerance in *Z. mobilis*.

## Conclusion

The effects of a broad range of temperatures at 24, 30, 36, 40, and 45 °C on cell growth, fermentation performance, heterologous protein expression, and global transcriptional profiles of *Z. mobilis* have been systematically investigated in this study. Our result indicated that temperature especially high temperature affected cell morphology and growth, sugar utilization and ethanol production, as well as the expression of exogenous protein such as EGFP. In addition, temperature affected the transcriptional profiles of *Z. mobilis* especially at high temperature with 478 genes up-regulated and 481 genes down-regulated in ZM4 cultured at 45 °C compared to 30 °C.

Genes encoding chaperonins, cell division proteins, and out membrane transporters were suggested to be associated with temperature increase, and therefore were selected together with other candidate genes obtained through bioinformatics studies and literature report for genetics studies to explore their roles on heat tolerance using strategies of plasmid overexpression and inducible promoter replacement. Genetics studies indicated that overexpression of *ZMO0236*, *ZMO1335*, *ZMO0994*, *groESL*, and an exogenous gene *cspL* can improve the heat tolerance of *Z. mobilis*. The heat produced by microorganisms during growth can raise the temperature of fermentation environment and then inhibit cell growth and fermentation performance, which needs costly cooling equipment to maintain normal cell growth. Our work thus not only explored the effects of temperature on the expression of endogenous and exogenous genes, but also selected and confirmed several genes associated with heat tolerance in *Z. mobilis*, which provided both gene candidates for heat-tolerant recombinant strain development and a guidance on identifying candidate genes associated with phenotypic improvement through systems biology strategy and genetics studies.

## Materials and methods

### Medium, strains, and chemicals

Rich Medium (RM) (50 g/L glucose, 10 g/L yeast extract, 2 g/L KH_2_PO_4_) autoclaved at 108 °C for 30 min was used to culture ZM4 (ATCC 31821) and derived mutants. Luria–Bertani (LB) culture (10 g/L tryptone, 5 g/L yeast extract, 10 g/L NaCl, LB solid medium requires additional 15 g/L agar) autoclaved at 121 °C for 30 min was used to culture *E. coli* DH5α. Glucose, KH_2_PO_4_, and NaCl were purchased from Sinopharm Chemical Reagent Co., Ltd (Shanghai, China). Yeast extract was purchased from OXOID. Agar was purchased from Guangzhou Saiguo Biotech Co., Ltd (Guangzhou, China).

### Flask fermentation and analytic methods

ZM4 or ZM4_EGFP revived in an appropriate volume of RM at 30 °C for about 6–8 h at 30 °C in RM was used as seed culture. The seed culture of *Z. mobilis* was then used to inoculate the shake flask containing 80% of RM with a sealing gas permeable membrane sealed at an initial OD_600_ of 0.1, and cultured at different temperatures (24, 30, 36, 40 and 45 °C), 100 rpm. At least three replicates were used for each condition. It should be noted that 50 μg/mL spectinomycin was added to the culture medium (RM) of the ZM4_EGFP to prevent the loss of pEZ_EGFP under high temperature.

The OD_600_ value of the bacterial culture was measured by UV–visible spectrophotometer UV-1800 (AoYi Instrument Co., Ltd, Shanghai, China) every 3 h. At the same time, 1-mL culture was centrifuged (12,000 rpm, 1 min) to obtain the supernatant for measuring the glucose and ethanol concentrations in the culture using Biosensor analyzer M-100 (Sieman Technology Co., Ltd., Shenzhen, China), and a Shimadzu LC-2030 high pressure liquid chromatography (HPLC) equipped with refractive index detector (RID) and Bio-Rad Aminex HPX-87H (300 × 7.8 mm) column [42]. Briefly, the supernatant was filtered through a 0.45-μm filter before applying on HPLC. 0.005 M H_2_SO_4_ was used as the mobile phase at a flow rate of 0.5 mL/min, and temperatures of the detector and column were 40 and 60 °C, respectively.

At 24 h, ZM4 cultured at different temperatures were stained by Gram-stain kit (Qingdao Hope Bio-Technology Co., Ltd, Qingdao, China) and then observed using Leica DMi8 fluorescence microscope. The live ZM4_EGFP cultured at different temperatures were observed directly using Leica DMi8 fluorescence microscope after washing twice with 1× PBS.

### Transcriptomic analysis and statistical analysis

The transcriptomics study was followed previous work [37, 42–49]. Briefly, cell culture samples at different temperatures were collected during the mid-exponential phase followed by total RNA extraction and rRNA depletion using TRIzol reagent (Invitrogen, USA) and Ribo-Zero rRNA Removal Kit (Illumina, USA), respectively. RNA-Seq was performed using paired-end sequencing technology according to standard Illumina protocols with a library construction kit (NEBNext^®^ Ultra™ Directional RNA Library Prep Kit for Illumina^®^) and an Illumina HiSeq instrument (Genewiz Inc, China).

RNA-Seq fastq data passing the quality control was evaluated using FastQC software (Babraham Bioinformatics, UK) before importing into CLC Genomics Workbench (Ver. 11.0) for reads trimming and RNA-Seq analysis to get the RPKM value (reads mapping to the genome per kilobase of transcript per million reads sequenced) of each gene with the reference genome. Genome sequence of *Z. mobilis* was used as the reference for RPKM calculation [50]. The RPKM value of each gene was then imported into JMP Genomics (Ver. 9.0, SAS Inc., NC, USA). Data normalization and statistical analysis were conducted to identify differentially expressed genes. Triplicate samples were used for each condition. The statistical analysis involved in this study including *t*-test and one-way ANOVA, were all conducted through JMP Genomics (Ver. 9.0, SAS Inc., NC, USA).

### Construction of recombinant strains

#### Construction of ZM4_EGFP strain for exogenous EGFP gene expression

**Fig. 7:**
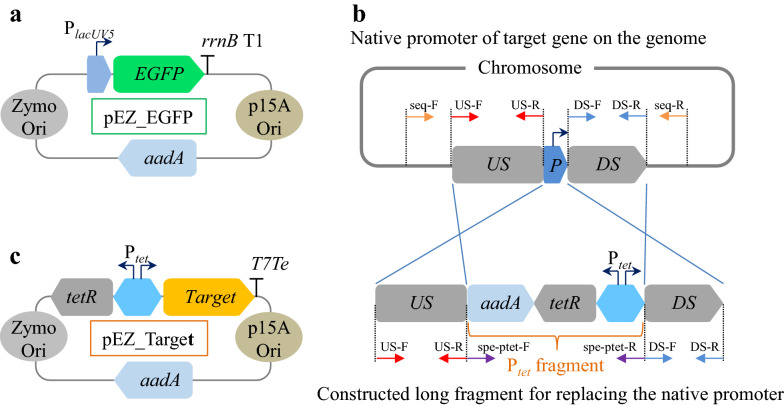
Schematic diagram of plasmids and promoter replacement method. The constructed pEZ_EGFP (**a**), long fragment for replacing the native promoter of target gene on the genome (**b**), and pEZ_Target (Target means one of these three constructs: *clpB*, an operon containing *clpB_groESL* with *groESL* driven by their native promoter, and *ZMO0994*) (**c**). P_*lacUV5*_ is a constitutive promoter. *rrnB* T1 and *T7Te* are terminators. Zymo Ori means an origin of replication with promoters for *Z. mobilis*. p15A Ori means an origin of replication with promoters for *E. coli*. *P* refers to the original promoter of the gene of interest. *US* means the upstream of the original promoter. *aadA* refers to spectinomycin resistance gene. P_*tet*_ is a bi-directional promoter, which simultaneously initiates the expression of *tetR* gene and the candidate gene. P_*tet*_ fragment included *aadA*, *tetR,* and P_*tet*_

The reporter gene *EGFP* driven by a constitutive promoter P_*lacUV5*_ was assembled to the shuttle vector pEZ15Asp containing an origin of replication with promoters for *E. coli* as well as *Z. mobilis* [41] to construct the plasmid pEZ_EGFP (Fig. 7a). The plasmid was then transferred to ZM4 competent cells prepared as described previously through electroporation (electroporation condition: 1 mm electrode gap, 1600 V, 200 Ω, 25 μF) using a Gene Pulser^®^ (Bio-rad, USA) [28, 41]. Subsequently, the cells were spread on a plate containing 100 μg/mL spectinomycin and cultured at 30 °C for approximately 2 days. The transformants were verified by colony PCR, and then confirmed by Sanger sequencing to obtain the recombinant strain ZM4_EGFP.

#### Construction of mutant with native promoter replaced by inducible promoter P_*tet*_

The native promoter of candidate gene was predicted using BPROM promoter prediction webserver at http://www.softberry.com. Upstream (US) and downstream (DS) with length between 500 and 1000 bp of the promoter were used as homology arms (Fig. 7b).

DNA fragment used to replace native promoters abbreviated as P_*tet*_ fragment includes the spectinomycin resistance gene *aadA*, *tetR* gene encoding the TetR repressor protein and an inducible promoter P_*tet*_ (Fig. 7b). The primer pairs for amplifying upstream (primers: US-F, US-R) and downstream (primers: DS-F, DS-R) homology arms and the promoter to be replaced (spe-ptet-F, spe-ptet-R) were designed, and all of them contained 15–20 nucleotides overlapping regions (Additional file 4: Table S4). Among them, the primers US-F and DS-R overlapped with the cloning vector pUC57 by about 20 bp. The upstream and downstream homology arms of native promoters of candidate genes were amplified from ZM4 genomic DNA. Three DNA fragments including the upstream and downstream homology arms of native promoters of candidate genes and the P_*tet*_ fragment were assembled by overlapping PCR.

The overlapping PCR method includes two steps. The first step was to assemble a long fragment through the three fragments above. The upstream and downstream homology arms and the P_*tet*_ fragment were all 100 ng, and 10 μL 2× PrimerStar mix was added, water was then added to a total volume of 20 μL. The reaction procedure for the first step was set as following: 98 °C 3 min, (98 °C 15 s, 44 °C 20 s, 72 °C 30 s) for 12 cycles, 72 °C 2 min. One microliter (1 μL) primers of US-F and DS-R were added into 20 μL reaction system from the first step at the second step. The reaction procedure for the second step was set as following: 98 °C 3 min, (98 °C 15 s, 55 °C 20 s, 72 °C 1 min) for 25 cycles, 72 °C 2 min.

PCR products were separated by gel electrophoresis, followed by gel purification, and subsequently quantified using NanoDrop 2000 (Thermo Fisher Scientific, USA). The long fragments from overlapping PCR and vector pUC57 were assembled by using T5 exonuclease (NEB, USA) [28], and the obtained recombinant plasmids were verified by PCR using US-F and DS-R and Sanger sequencing. The sequencing confirmed plasmid was then transferred to ZM4 competent cells prepared as described previously through electroporation (electroporation condition: 1 mm electrode gap, 1600 V, 200 Ω, 25 μF.) using a Gene Pulser^®^ (Bio-Rad, USA) to obtain the recombinant strains [28, 41]. Subsequently, the cells were spread on a plate containing 100 μg/mL spectinomycin and cultured at 30 °C for approximately 2 days. The transformants were verified using primer pairs of seq-F and seq-R that were outside the US-F and DS-R and further confirmed using Sanger Sequencing to obtain the final mutants.

#### Construction of recombinant strains over-expressing candidate gene

The target gene (like *clpB*, *ZMO0994*) driven by an inducible promoter P_*tet*_ was assembled to the vector pEZ15Asp [41] to construct the plasmid pEZ_Target (Fig. 7c), which was then transformed into ZM4 to obtain ZM4 (pEZ_Target). The procedures for competent cell preparation, electroporation, and transformant confirmation were same as described above. The sequence information of all primers as well as vectors, genes and biological parts used in this article are listed in Additional file 4: Table S4 and Additional file 5: Table S5.

### Flow cytometry and plate reader analysis

ZM4_EGFP cultured at different temperatures were washed with 1× phosphate-buffered saline (PBS) twice and then resuspended into PBS. Cells were analyzed by flow cytometry using Beckman CytoFLEX FCM (Beckman Coulter, USA) with the PBS as the sheath fluid, or using the SpectraMax M2e Microplate Reader (Molecular Devices, USA). The fluorescence of EGFP was excited with the 488 nm and detected with FITC by flow cytometry, and excited with the 485 nm by enzyme-labeled instrument.

### Western bolt

ZM4_EGFP grown at different temperatures was harvested to conduct the western blot experiment followed previous work [28]. Cells cultured at different temperatures were harvested and lysed for total protein extraction using Protein Extraction Kit (Zomanbio, China). Total protein concentrations of samples were measured by the Bradford method. Sodium dodecyl sulphate polyacrylamide gel electrophoresis (SDS-PAGE) was performed with a 5% stacking and a 12% running gel. The loading amount of each sample was 200 ng total protein, and a pre-stained protein ladder (10–170 kDa, Thermo, Lithuania) was loaded for estimating molecular weight [51].

Gel was transferred to methanol-activated PVDF membranes^®^ using the Trans-Blot Semi-Dry Electrophoretic Transfer Cell (Bio-Rad, USA) and run for 20 min at 25 V. PVDF membranes was then blocked with 5% skimmed milk in phosphate-buffered saline with Tween 20 (PBST) for 1 h at room temperature, and subsequently EGFP was probed with the primary antibody (1:5000, Proteintech, China) for 1 h. Peroxidase-conjugated goat anti-Mouse IgG (1:5000, Proteintech, China) was used as secondary antibody. PVDF membrane after blocking with skimmed milk, incubating primary antibody, and incubating secondary antibody was washed to remove the excess protein. Color development was performed by West Dure Extended Duration Substrate Kit (AntGene, China). All images were visualized using AI600 Imaging System (GE, USA).

### Characterization of recombinant strains

Recombinant strains with the native promoter of candidate genes replaced and candidate genes overexpressed revived in an appropriate volume of RM at 30 °C for about 6–8 h prior to inoculating overnight seed cultures at 30 °C in RM was used as seed culture.

The seed cultures of recombinant strains were centrifuged (12,000 rpm, 1 min) to remove RM. Recombinant strains were resuspended, respectively, with RM and RM with 0.2 µg/mL tetracycline, which were then inoculated into 96-well plate containing a total volume of 200 µL test medium at an initial OD_600_ value of 0.05. Cells were incubated without shaking at 40 °C. Triplicate were used for each condition, and turbidity measurements (OD_600_) were taken every 15 min by the FLUOstar^®^ Omega (BMG LABTECH, Germany) till cells grew into stationary phase.

## Supplementary Information

**Additional file 1: Table S1.** Glucose consumption (*Y*_s_), ethanol titer (*Y*_p_), ethanol yield (*Y*_p/s_), ethanol productivity (*Q*_P_), and specific growth rate (*μ*) of ZM4 or ZM4_GFP at different temperatures within 27 h. Multiple comparisons of each parameter of ZM4 or ZM4_GFP at 24, 36, 40, and 45 °C were conducted with ZM4 or ZM4_GFP at 30 °C as a control, * represents a significant difference (0.01 < *P*-value < 0.05), ** represents a very significant difference (*P*-value < 0.01), *** represents *P*-value < 0.001, **** represents *P*-value < 0.0001.

**Additional file 2: Table S2.** List the differentially expressed genes of ZM4 at different temperatures. It contained a total of 6 tables which were Table S2_a, Table S2_b, Table S2_c, Table S2_d, Table S2_e, and Table S2_f, listing differentially expressed genes between ZM4 cultured at 30 °C and 45 °C (30/45), 30 °C and 40 °C (30/40), 30 °C and 36 °C (30/36), 24 °C and 30 °C (24/30), 40 °C and 45 °C (40/45), 36 °C and 40 °C (36/40).

**Additional file 3: Table S3.** List of eleven up-regulated genes of ZM4 cultured at 24 °C and 45 °C compared with 30 °C. Ratio (24/30) is the log_2_-based expression difference between ZM4 cultured at 24 °C and 30 °C. Ratio (45/30) is the log_2_-based expression difference between ZM4 cultured at 45 °C and 30 °C. *P*-value is − log_10_(*P*-value).

**Additional file 4: Table S4. **List of primers used in this work. The underlined sequence indicates the homology arm for overlapping PCR or plasmid assembly by T5 exonuclease.

**Additional file 5: Table S5.** Sequences of pEZ15A and pUC57 vectors, *EGFP *and *cspL* genes, as well as promoters and terminators used in this study.

## Data Availability

The RNA-Seq raw data were deposited at Sequence Read Archive (SRA) database.

## Abbreviations

ED: Entner–Doudoroff
EMP: Embden–Meyerhof–Parnas
GRAS: Generally regarded as safe status
RM: Rich medium
RPKM: Reads mapping to the genome per kilobase of transcript per million reads sequenced
