# Supplementary material for: Investigation of the impact of a broad range of temperatures on the physiological and transcriptional profiles of Zymomonas mobilis ZM4 for high-temperature-tolerant recombinant strain development
Source: Biotechnol Biofuels. 2021 Jun 27;14:146. doi: 10.1186/s13068-021-02000-1 (PMC8237431; doi:10.1186/s13068-021-02000-1)
Supplement: Supplementary file 1 — Additional file 1: Table S1. Glucose consumption (Ys), ethanol titer (Yp), ethanol yield (Yp/s), ethanol productivity (QP), and specific growth rate (μ) of ZM4 or ZM4_GFP at different temperatures within 27 h. Multiple comparisons of each parameter of ZM4 or ZM4_GFP at 24, 36, 40, and 45 °C were conducted with ZM4 or ZM4_GFP at 30 °C as a control, * represents a significant difference (0.01 < P-value < 0.05), ** represents a very significant difference (P-value < 0.01), *** represents P-value < 0.001, **** represents P-value < 0.0001. [file 13068_2021_2000_MOESM1_ESM.docx]

**Table S1.** Glucose consumption (*Y_s_*), ethanol titer (*Y_p_*), ethanol yield (*Y_p/s_*), ethanol productivity (*Q_P_*), and specific growth rate (*μ*) of ZM4 or ZM4_GFP at different temperatures within 27 h. Multiple comparisons of each parameter of ZM4 or ZM4_GFP at 24, 36, 40, and 45℃ were conducted with ZM4 or ZM4_GFP at 30℃ as a control, * represents a significant difference (0.01<*P*-value<0.05), ** represents a very significant difference (*P*-value<0.01), *** represents *P*-value<0.001, **** represents *P*-value<0.0001.

| **Strains** | **Conditions** | ***Y_s_* (g/L)** | ***Y_p_* (g/L)** | ***Y_p/s_* (g/g)** | ***Q_p_* (g/L/h)** | ***μ* (h^-1^)** |
| --- | --- | --- | --- | --- | --- | --- |
| ZM4 | 24°C | 43.0±3.5 | 24.7±2.0 | 0.58±0.05 | 0.91±0.07 | 0.21±0.03^⁎⁎⁎⁎^ |
| ZM4 | 30°C | 49.0±2.2 | 26.2±0.3 | 0.53±0.02 | 0.97±0.01 | 0.31±0.01 |
| ZM4 | 36°C | 48.2±2.3 | 24.2±0.3 | 0.50±0.02 | 0.90±0.01 | 0.42±0.01^⁎⁎⁎⁎^ |
| ZM4 | 40°C | 48.0±1.7 | 22.8±1.0 | 0.48±0.03 | 0.85±0.04 | 0.44±0.01^⁎⁎⁎⁎^ |
| ZM4 | 45°C | 19.7±6.8^⁎⁎⁎⁎^ | 8.5±3.3^⁎⁎⁎⁎^ | 0.33±0.02^⁎⁎⁎⁎^ | 0.35±0.04^⁎⁎⁎⁎^ | 0.18±0.01^⁎⁎⁎⁎^ |
| ZM4_GFP | 24°C | 42.2±3.2^⁎^ | 21.7±0.8^⁎⁎⁎⁎^ | 0.52±0.03 | 0.80±0.03^⁎⁎⁎⁎^ | 0.20±0.02^⁎⁎⁎^ |
| ZM4_GFP | 30°C | 49.7±2.0 | 25.3±0.3 | 0.51±0.02 | 0.94±0.01 | 0.28±0.01 |
| ZM4_GFP | 36°C | 49.7±2.0 | 24.0±0.5^⁎^ | 0.48±0.01 | 0.89±0.02^⁎^ | 0.38±0.01^⁎⁎⁎⁎^ |
| ZM4_GFP | 40°C | 49.7±2.0 | 24.5±0.5 | 0.49±0.02 | 0.91±0.02 | 0.34±0.02^⁎⁎^ |
| ZM4_GFP | 45°C | 9.2±2.8^⁎⁎⁎⁎^ | 3.8±0.4^⁎⁎⁎⁎^ | 0.44±0.15 | 0.14±0.01^⁎⁎⁎⁎^ | 0.14±0.01^⁎⁎⁎⁎^ |

The calculation formula of each parameter in the above table was as follows：

***Y_s_***= consumed glucose (g/L);

***Y_p_***= maximum ethanol titer (g/L);

***Y_p/s_***= maximum ethanol (g)/consumed glucose (g);

***Q_P_***= maximum ethanol titer (g/L)/time to reach the maximum ethanol.

***μ***=ln((OD_600_ at t_2_)/ (OD_600_ at t_1_))/(t_2_-t_1_), t_1_ and t_2_ are the time points in the log phase.
