# Supplementary material for: Investigation of the impact of a broad range of temperatures on the physiological and transcriptional profiles of Zymomonas mobilis ZM4 for high-temperature-tolerant recombinant strain development
Source: Biotechnol Biofuels. 2021 Jun 27;14:146. doi: 10.1186/s13068-021-02000-1 (PMC8237431; doi:10.1186/s13068-021-02000-1)
Supplement: Supplementary file 2 — Additional file 2: Table S2. List the differentially expressed genes of ZM4 at different temperatures. It contained a total of 6 tables which were Table S2_a, Table S2_b, Table S2_c, Table S2_d, Table S2_e, and Table S2_f, listing differentially expressed genes between ZM4 cultured at 30 °C and 45 °C (30/45), 30 °C and 40 °C (30/40), 30 °C and 36 °C (30/36), 24 °C and 30 °C (24/30), 40 °C and 45 °C (40/45), 36 °C and 40 °C (36/40). [file 13068_2021_2000_MOESM2_ESM.docx]

**Table S2** list the differentially expressed genes of ZM4 at different temperatures. It contained a total of 6 tables which were **Table S2_a**, **Table S2_b**, **Table S2_c**, **Table S2_d**, **Table S2_e**, and **Table S2_f**, listing differentially expressed genes between ZM4 cultured at 30℃ and 45℃ (**30/45**), 30℃ and 40℃ (**30/40**), 30℃ and 36℃ (**30/36**), 24℃ and 30℃ (**24/30**), 40℃ and 45℃ (**40/45**), 36℃ and 40℃ (**36/40**).

**Table S2_a.** List of significantly differentially expressed genes between ZM4 cultured at 30℃ and 45℃. Ratio is the log_2_-based expression difference between ZM4 cultured at 30℃ and 45℃ (30/45). Gene name with red and blue color font indicates up-regulated and down-regulated at 30℃ compared with 45℃, respectively.

| **Name** | **Product** | **Ratio** | **-log_10_ (p-value)** |
| --- | --- | --- | --- |
| **Upregulated gene in ZM4 cultured at 30℃ compared with 45℃** | | | |
| *ZMO0005* | sulfate adenylyltransferase small subunit | 1.35 | 3.24 |
| *ZMO0007* | phosphoadenosine phosphosulfate reductase | 1.08 | 2.19 |
| *ZMO0008* | sulfite reductase (NADPH) hemoprotein beta-component | 1.31 | 2.59 |
| *ZMO0013* | rdgB/HAM1 family non-canonical purine NTP pyrophosphatase | 1.54 | 8.74 |
| *ZMO0020* | LUD domain-containing protein | 1.73 | 7.87 |
| *ZMO0021* | iron-sulfur cluster-binding protein YkgF | 2.23 | 9.11 |
| *ZMO0022* | Fe-S oxidoreductase | 2.10 | 9.53 |
| *ZMO0026* | Sel1 domain protein repeat-containing protein | 1.04 | 5.33 |
| *ZMO0027* | bifunctional phosphoribosyl aminoimidazole carboxamide formyltransferase/IMP cyclohydrolase | 2.07 | 11.81 |
| *ZMO0028* | restriction endonuclease | 1.63 | 11.40 |
| *ZMO0030* | glyoxalase/bleomycin resistance protein/dioxygenase | 1.54 | 9.97 |
| *ZMO0040* | dephospho-CoA kinase | 1.01 | 7.75 |
| *ZMO0043* | Maf family protein | 1.08 | 6.64 |
| *ZMO0080* | chemoreceptor glutamine deamidase CheD | 1.67 | 9.79 |
| *ZMO0081* | chemotaxis signal transduction response regulator CheB | 2.12 | 9.57 |
| *ZMO0082* | CheR-type MCP methyltransferase | 2.04 | 9.41 |
| *ZMO0099* | major facilitator superfamily transporter | 1.06 | 7.85 |
| *ZMO0100* | HxlR family transcriptional regulator | 1.79 | 7.56 |
| *ZMO0130* | acid phosphatase | 1.46 | 9.15 |
| *ZMO0142* | YggS family pyridoxal phosphate enzyme | 1.24 | 8.17 |
| *ZMO0143* | ABC transporter related protein | 1.22 | 8.40 |
| *ZMO0149* | tRNA (guanine-N(7)-)-methyltransferase | 1.71 | 9.87 |
| *ZMO0152* | pyruvate kinase | 1.43 | 10.54 |
| *ZMO0160* | thioesterase superfamily protein | 1.10 | 7.69 |
| *ZMO0161* | MotA/TolQ/ExbB proton channel | 1.24 | 8.08 |
| *ZMO0162* | Tol-Pal cell envelope complex membrane spanning protein TolR | 1.19 | 7.83 |
| *ZMO0164* | Tol-Pal cell envelope complex membrane achoring protein TolA | 1.60 | 8.92 |
| *ZMO0175* | uncharacterized protein | 1.40 | 5.63 |
| *ZMO0184* | hypothetical protein | 2.58 | 8.71 |
| *ZMO0190* | RpiR family transcriptional regulator | 2.35 | 12.45 |
| *ZMO0195* | N-acetylmuramoyl-L-alanine amidase | 1.34 | 9.81 |
| *ZMO0202* | methyl-accepting chemotaxis sensory transducer | 2.16 | 11.56 |
| *ZMO0220* | ferredoxin | 1.17 | 7.70 |
| *ZMO0232* | SpoU type tRNA/rRNA methyltransferase | 1.08 | 8.57 |
| *ZMO0238* | ATP synthase F1 delta subunit | 1.49 | 8.04 |
| *ZMO0239* | ATP synthase F0F1 alpha subunit | 2.01 | 9.65 |
| *ZMO0240* | ATP synthase F0F1 gamma subunit | 2.24 | 9.72 |
| *ZMO0241* | ATP synthase F0F1 beta subunit | 1.64 | 9.16 |
| *ZMO0242* | ATP synthase F0F1 epsilon subunit | 1.53 | 8.98 |
| *ZMO0244* | HU family DNA-binding protein | 2.05 | 9.54 |
| *ZMO0264* | uncharacterized protein | 1.04 | 6.25 |
| *ZMO0265* | uncharacterized protein | 1.21 | 8.89 |
| *ZMO0270* | uncharacterized protein | 2.03 | 8.09 |
| *ZMO0271* | apolipoprotein N-acyltransferase | 1.40 | 10.10 |
| *ZMO0276* | OstA family protein | 2.01 | 10.87 |
| *ZMO0278* | ribonuclease D | 2.15 | 12.41 |
| *ZMO0296* | tRNA-specific adenosine deaminase | 1.25 | 5.26 |
| *ZMO0305* | stress responsive alpha-beta barrel domain protein | 1.24 | 8.80 |
| *ZMO0306* | glycosyl transferase group 1 | 1.22 | 6.44 |
| *ZMO0307* | capsule polysaccharide biosynthesis protein | 1.39 | 7.50 |
| *ZMO0321* | dihydrofolate reductase region | 1.39 | 9.57 |
| *ZMO0322* | riboflavin biosynthesis protein RibF | 1.19 | 5.43 |
| *ZMO0329* | inositol monophosphatase | 1.24 | 8.90 |
| *ZMO0331* | DUF882 domain-containing protein | 2.04 | 10.83 |
| *ZMO0343* | aminotransferase class IV | 1.17 | 8.06 |
| *ZMO0345* | endonuclease/exonuclease/phosphatase | 1.41 | 10.23 |
| *ZMO0361* | uncharacterized protein | 1.12 | 4.85 |
| *ZMO0366* | glucose facilitated diffusion protein | 2.13 | 13.21 |
| *ZMO0372* | AsnC family transcriptional regulator | 1.35 | 5.22 |
| *ZMO0373* | hypothetical protein | 1.03 | 4.58 |
| *ZMO0374* | levansucrase | 1.83 | 5.90 |
| *ZMO0375* | levansucrase/invertase | 1.64 | 5.43 |
| *ZMO0379* | PBSX family phage terminase large subunit | 1.53 | 5.18 |
| *ZMO0395* | hypothetical protein | 2.20 | 5.52 |
| *ZMO0397* | hypothetical protein | 1.84 | 4.79 |
| *ZMO0413* | YggT family protein | 1.10 | 7.17 |
| *ZMO0445* | GDSL lipase/esterase | 1.57 | 10.25 |
| *ZMO0446* | membrane bound O-acyl transferase MBOAT family protein | 2.23 | 11.78 |
| *ZMO0454* | formate--tetrahydrofolate ligase | 2.39 | 10.71 |
| *ZMO0456* | ferredoxin | 1.68 | 11.35 |
| *ZMO0457* | glutamate uptake regulatory protein | 1.46 | 10.53 |
| *ZMO0458* | lipoprotein-releasing system ATP-binding protein LolD | 1.49 | 9.16 |
| *ZMO0459* | lipoprotein releasing system transmembrane protein LolC/E family | 1.33 | 10.88 |
| *ZMO0460* | proline--tRNA ligase | 1.23 | 9.65 |
| *ZMO0462* | CTP synthase | 1.56 | 9.98 |
| *ZMO0464* | preprotein translocase SecG subunit | 1.21 | 7.68 |
| *ZMO0467* | PpiC-type peptidyl-prolyl cis-trans isomerase | 1.29 | 8.23 |
| *ZMO0476* | riboflavin biosynthesis protein RibD | 1.32 | 8.51 |
| *ZMO0477* | uncharacterized protein | 1.33 | 8.20 |
| *ZMO0480* | histidine kinase | 1.24 | 10.54 |
| *ZMO0481* | uncharacterized protein | 1.69 | 10.26 |
| *ZMO0483* | homoserine dehydrogenase | 1.54 | 12.02 |
| *ZMO0492* | nitrogen regulatory protein PII | 3.16 | 11.78 |
| *ZMO0493* | type I glutamine synthetase | 2.24 | 10.69 |
| *ZMO0497* | phosphoglycolate phosphatase | 1.30 | 7.48 |
| *ZMO0500* | uncharacterized protein | 1.75 | 10.34 |
| *ZMO0501* | polysaccharide synthesis protein GtrA | 1.09 | 7.37 |
| *ZMO0502* | uncharacterized protein | 1.41 | 10.97 |
| *ZMO0503* | glycosyl transferase family 2 | 2.27 | 11.36 |
| *ZMO0505* | pseudouridine synthase | 1.00 | 7.00 |
| *ZMO0529* | ribosomal protein S14 | 1.02 | 8.85 |
| *ZMO0530* | ribosomal protein S8 | 1.16 | 8.44 |
| *ZMO0531* | ribosomal protein L6 signature 1 | 1.50 | 9.38 |
| *ZMO0532* | ribosomal protein L18 | 1.30 | 7.33 |
| *ZMO0533* | ribosomal protein S5 | 1.24 | 7.54 |
| *ZMO0534* | ribosomal protein L30 | 1.59 | 7.93 |
| *ZMO0537* | preprotein translocase SecY subunit | 1.70 | 11.32 |
| *ZMO0543* | aconitate hydratase | 1.95 | 10.00 |
| *ZMO0544* | isocitrate dehydrogenase NADP-dependent | 1.56 | 8.09 |
| *ZMO0545* | bifunctional N-(5-phosphoribosyl)anthranilate isomerase/indole-3-glycerolphosphate synthase | 1.52 | 7.78 |
| *ZMO0548* | signal transduction histidine kinase | 2.75 | 10.40 |
| *ZMO0549* | polyribonucleotide nucleotidyltransferase | 1.40 | 9.84 |
| *ZMO0568* | succinate dehydrogenase (SQR) hydrophobic subunit | 1.24 | 9.17 |
| *ZMO0573* | glutaredoxin 2 GrxB family | 1.32 | 7.09 |
| *ZMO0575* | RsmD family protein | 1.43 | 8.90 |
| *ZMO0576* | pseudouridine synthase | 1.34 | 9.57 |
| *ZMO0578* | sodium:dicarboxylate symporter | 1.76 | 6.65 |
| *ZMO0581* | major facilitator superfamily transporter | 1.14 | 8.64 |
| *ZMO0583* | acetyl-CoA carboxylase beta subunit | 1.06 | 9.39 |
| *ZMO0584* | tryptophan synthase alpha subunit | 2.16 | 11.80 |
| *ZMO0585* | tryptophan synthase beta subunit | 1.39 | 9.41 |
| *ZMO0586* | phosphoribosylanthranilate isomerase | 1.18 | 6.84 |
| *ZMO0589* | DNA repair protein RadA | 1.04 | 7.62 |
| *ZMO0602* | flagellar motor protein MotB | 1.77 | 9.50 |
| *ZMO0603* | flagellar motor protein MotA | 2.20 | 13.52 |
| *ZMO0605* | flagellar hook-associated protein FlgK | 1.38 | 8.42 |
| *ZMO0606* | flagellar rod assembly protein/muramidase FlgJ | 2.46 | 12.27 |
| *ZMO0607* | flagellar P-ring protein | 2.40 | 13.04 |
| *ZMO0608* | flagellar L-ring protein | 2.17 | 10.86 |
| *ZMO0609* | flagellar basal-body rod protein FlgG | 2.40 | 12.41 |
| *ZMO0610* | flagellar basal-body rod protein FlgF | 2.71 | 13.16 |
| *ZMO0611* | flagellar hook protein FlgE | 3.15 | 14.30 |
| *ZMO0612* | flagellar hook capping protein | 3.26 | 12.54 |
| *ZMO0613* | flagellar basal-body rod protein FlgC | 2.95 | 12.21 |
| *ZMO0614* | flagellar basal-body rod protein FlgB | 4.11 | 12.38 |
| *ZMO0624* | flagellar biosynthesis protein FlhA | 1.41 | 10.15 |
| *ZMO0626* | RNA polymerase sigma 28 subunit FliA/WhiG | 1.57 | 7.75 |
| *ZMO0627* | GT2 family glycosyltransferase | 2.18 | 11.12 |
| *ZMO0631* | Fis family sigma54-specific transcriptional regulator | 3.35 | 11.66 |
| *ZMO0632* | flagellar hook-basal body complex subunit FliE | 2.46 | 11.20 |
| *ZMO0634* | flagellar M-ring protein FliF | 2.28 | 11.06 |
| *ZMO0635* | flagellar motor switch protein FliG | 3.24 | 10.81 |
| *ZMO0636* | negative regulator of FliI ATPase | 1.45 | 9.58 |
| *ZMO0637* | flagellum-specific ATP synthase FliI | 1.00 | 8.92 |
| *ZMO0641* | pseudo gene | 1.07 | 6.98 |
| *ZMO0642* | flagellar basal body-associated protein FliL | 1.69 | 10.67 |
| *ZMO0646* | flagellar biosynthetic protein FliO | 1.35 | 7.06 |
| *ZMO0647* | flagellar biosynthesis protein FliP | 1.25 | 6.86 |
| *ZMO0654* | polysaccharide deacetylase | 1.10 | 6.33 |
| *ZMO0655* | adenosine/AMP deaminase | 1.53 | 9.95 |
| *ZMO0662* | adenylosuccinate lyase | 1.57 | 10.58 |
| *ZMO0667* | ATP synthase F0F1 A subunit | 1.47 | 9.78 |
| *ZMO0668* | ATP synthase F0F1 C subunit | 1.65 | 10.15 |
| *ZMO0669* | ATP synthase F0F1 B/B' subunit | 2.32 | 10.51 |
| *ZMO0671* | ATP synthase F0F1 B/B' subunit | 1.18 | 7.02 |
| *ZMO0677* | 3-isopropylmalate dehydrogenase | 2.06 | 10.24 |
| *ZMO0686* | signal transduction protein | 1.13 | 3.64 |
| *ZMO0687* | acetolactate synthase large subunit | 3.17 | 9.27 |
| *ZMO0689* | glucose-fructose oxidoreductase | 1.99 | 9.43 |
| *ZMO0691* | tRNA (uracil-5-)-methyltransferase Gid | 1.05 | 9.41 |
| *ZMO0708* | phosphoribosylglycinamide formyltransferase | 1.04 | 7.42 |
| *ZMO0715* | aspartate--tRNA(Asp/Asn) ligase | 1.18 | 7.79 |
| *ZMO0735* | acetyl-CoA carboxylase complex biotin carboxylase subunit | 1.84 | 10.52 |
| *ZMO0736* | acetyl-CoA carboxylase complex biotin carboxyl carrier protein | 1.90 | 11.33 |
| *ZMO0738* | thiazole biosynthesis protein ThiG | 1.11 | 7.28 |
| *ZMO0743* | peptide chain release factor 3 | 1.88 | 10.15 |
| *ZMO0744* | uncharacterized protein | 1.45 | 7.27 |
| *ZMO0751* | Mov34/MPN/PAD-1 family protein | 1.57 | 9.74 |
| *ZMO0752* | histidine phosphotransferase | 1.58 | 10.27 |
| *ZMO0756* | TPR repeat-containing protein | 2.07 | 10.66 |
| *ZMO0757* | TPR repeat-containing protein | 1.86 | 8.92 |
| *ZMO0758* | isochorismatase hydrolase | 1.78 | 10.94 |
| *ZMO0759* | hydroxyacylglutathione hydrolase | 1.25 | 9.75 |
| *ZMO0765* | threonyl-tRNA synthetase | 1.93 | 11.22 |
| *ZMO0767* | uncharacterized protein | 2.08 | 12.03 |
| *ZMO0768* | hypothetical protein | 1.58 | 7.91 |
| *ZMO0769* | tRNA 2-thiouridine(34) synthase MnmA | 1.10 | 7.01 |
| *ZMO0770* | magnesium transporter | 1.88 | 12.63 |
| *ZMO0773* | peptidyl-prolyl cis-trans isomerase cyclophilin type | 2.37 | 9.70 |
| *ZMO0778* | inner membrane transporter of efflux system | 1.22 | 9.24 |
| *ZMO0779* | RND efflux pump membrane fusion protein | 1.63 | 9.28 |
| *ZMO0780* | AcrB-like multidrug efflux transporter | 2.66 | 11.64 |
| *ZMO0782* | glutamyl-tRNA(Gln) amidotransferase B subunit | 2.55 | 13.02 |
| *ZMO0783* | glutamyl-tRNA(Gln) amidotransferase A subunit | 3.18 | 13.06 |
| *ZMO0784* | glutamyl-tRNA(Gln) amidotransferase C subunit | 3.12 | 12.99 |
| *ZMO0785* | Holliday junction resolvase YqgF | 1.34 | 6.70 |
| *ZMO0791* | aspartate carbamoyltransferase | 1.10 | 7.89 |
| *ZMO0797* | short-chain dehydrogenase/reductase SDR | 1.14 | 6.22 |
| *ZMO0800* | ABC transporter related protein | 1.26 | 7.07 |
| *ZMO0801* | secretion protein HlyD family protein | 2.17 | 7.93 |
| *ZMO0803* | sporulation domain protein | 1.79 | 9.84 |
| *ZMO0804* | N-acetyl-gamma-glutamyl-phosphate reductase | 1.80 | 9.04 |
| *ZMO0806* | cytosol aminopeptidase | 2.22 | 11.35 |
| *ZMO0807* | ribosomal protein S12 methylthiotransferase RimO | 2.38 | 12.50 |
| *ZMO0820* | phosphoribosylformylglycinamidine synthase subunit PurL | 1.26 | 9.55 |
| *ZMO0822* | uncharacterized protein | 1.11 | 6.46 |
| *ZMO0825* | penicillin-binding protein PBP2 | 1.39 | 10.52 |
| *ZMO0826* | UDP-N-acetylmuramoyl-L-alanyl-D-glutamate-2, 6-diaminopimelate ligase | 2.08 | 13.09 |
| *ZMO0827* | UDP-N-acetylmuramoyl-tripeptide-D-alanyl-D- alanine ligase | 2.51 | 13.96 |
| *ZMO0828* | phospho-N-acetylmuramoyl-pentapeptide transferase | 3.34 | 13.49 |
| *ZMO0829* | UDP-N-acetylmuramoyl-L-alanine-D-glutamate ligase | 2.72 | 13.63 |
| *ZMO0830* | cell division protein FtsW | 2.16 | 11.89 |
| *ZMO0831* | undecaprenyldiphospho-muramoylpentapeptide beta-N-acetylglucosaminyltransferase | 2.17 | 12.50 |
| *ZMO0832* | UDP-N-acetylmuramate-L-alanine ligase | 2.01 | 10.69 |
| *ZMO0833* | UDP-N-acetylenolpyruvoylglucosamine reductase | 2.58 | 12.51 |
| *ZMO0834* | D-alanine-D-alanine ligase | 2.24 | 12.89 |
| *ZMO0835* | cell division protein FtsQ | 2.05 | 11.18 |
| *ZMO0840* | sporulation domain-containing protein | 1.68 | 9.53 |
| *ZMO0841* | chloride channel core | 2.11 | 9.33 |
| *ZMO0845* | alanine--tRNA ligase | 1.89 | 12.45 |
| *ZMO0847* | carbohydrate-selective porin OprB | 1.02 | 8.08 |
| *ZMO0852* | tRNA preQ1(34) S-adenosylmethionine ribosyltransferase-isomerase QueA | 1.63 | 9.89 |
| *ZMO0854* | pantetheine-phosphate adenylyltransferase | 1.50 | 11.26 |
| *ZMO0855* | geranylgeranyl pyrophosphate synthase | 2.53 | 13.13 |
| *ZMO0856* | exodeoxyribonuclease VII small subunit | 1.66 | 11.15 |
| *ZMO0857* | DUF1013 domain-containing protein | 2.72 | 10.07 |
| *ZMO0859* | aminotransferase class V | 2.05 | 9.97 |
| *ZMO0861* | DNA polymerase III subunits gamma and tau | 1.19 | 6.40 |
| *ZMO0864* | cytidine deaminase | 1.25 | 10.74 |
| *ZMO0866* | cation diffusion facilitator family transporter | 3.49 | 11.57 |
| *ZMO0867* | hopanoid-associated sugar epimerase HpnA | 2.29 | 13.10 |
| *ZMO0868* | hopene-associated glycosyltransferase HpnB | 1.95 | 12.71 |
| *ZMO0869* | squalene synthase HpnC | 1.86 | 12.48 |
| *ZMO0870* | squalene synthase HpnD | 1.42 | 9.87 |
| *ZMO0871* | squalene-associated FAD-dependent desaturase HpnE | 1.58 | 11.43 |
| *ZMO0872* | squalene-hopene cyclase | 1.97 | 11.96 |
| *ZMO0873* | hopanoid-associated phosphorylase | 1.22 | 7.81 |
| *ZMO0880* | putative CheA signal transduction histidine kinase | 1.08 | 8.11 |
| *ZMO0883* | ribosomal protein S9 | 1.92 | 10.00 |
| *ZMO0884* | ribosomal protein L13 | 2.44 | 11.45 |
| *ZMO0886* | DUF192 domain-containing protein | 1.64 | 7.54 |
| *ZMO0896* | uncharacterized protein | 1.41 | 11.04 |
| *ZMO0919* | diguanylate cyclase with beta propeller sensor | 1.33 | 8.11 |
| *ZMO0936* | EamA/RhaT family transporter | 1.41 | 8.25 |
| *ZMO0937* | aromatic-amino-acid transaminase | 1.25 | 8.94 |
| *ZMO0946* | trigger factor | 1.52 | 9.39 |
| *ZMO0947* | glycosyl transferase family 2 | 2.11 | 11.77 |
| *ZMO0956* | ubiquinol-cytochrome c reductase iron-sulfur subunit | 1.23 | 6.77 |
| *ZMO0957* | cytochrome b | 1.19 | 10.84 |
| *ZMO0969* | xanthine/uracil/vitamin C permease | 1.94 | 11.14 |
| *ZMO0970* | purine nucleoside permease | 1.52 | 9.70 |
| *ZMO0971* | adenosine deaminase | 1.17 | 9.55 |
| *ZMO0972* | hopanoid biosynthesis associated glycosyl transferase protein HpnI | 1.16 | 8.75 |
| *ZMO0975* | putative membrane spanning protein | 2.33 | 11.66 |
| *ZMO0979* | TonB-dependent receptor | 1.12 | 7.10 |
| *ZMO0982* | ABC transporter permease | 1.52 | 7.06 |
| *ZMO0983* | type 1 ABC transporter solute-binding subunit | 1.04 | 8.58 |
| *ZMO0984* | lipoprotein NlpD | 1.82 | 10.34 |
| *ZMO0985* | 5'-nucleotidase SurE | 3.03 | 13.75 |
| *ZMO0986* | seryl-tRNA synthetase | 2.54 | 14.29 |
| *ZMO0997* | 2-dehydro-3-deoxyphosphogluconate aldolase/4-hydroxy-2-oxoglutarate aldolase | 1.11 | 9.78 |
| *ZMO1012* | uncharacterized protein | 1.22 | 8.03 |
| *ZMO1013* | septum formation inhibitor Maf | 2.65 | 10.22 |
| *ZMO1014* | translation initiation factor IF-1 | 2.68 | 10.45 |
| *ZMO1015* | DUF330 domain-containing protein | 1.31 | 8.12 |
| *ZMO1016* | MCE family protein | 1.66 | 10.50 |
| *ZMO1017* | iron ABC transporter ATP-binding protein | 2.04 | 10.04 |
| *ZMO1018* | DUF140 domain-containing protein | 2.09 | 13.87 |
| *ZMO1019* | deoxyhypusine synthase | 1.50 | 9.33 |
| *ZMO1020* | diaminopimelate decarboxylase LysA | 1.41 | 9.34 |
| *ZMO1027* | pyruvate-formate lyase-activating enzyme RflA | 1.15 | 7.06 |
| *ZMO1036* | argininosuccinate synthase ArgG | 1.61 | 10.91 |
| *ZMO1039* | ribonucleoside-diphosphate reductase alpha subunit | 1.73 | 12.45 |
| *ZMO1044* | large conductance mechanosensitive channel protein | 1.13 | 8.97 |
| *ZMO1055* | bifunctional diguanylate cyclase (GGDEF)/phosphodiesterase | 1.00 | 6.18 |
| *ZMO1056* | polynucleotide adenylyltransferase | 1.44 | 8.05 |
| *ZMO1057* | GCN5-related N-acetyltransferase | 2.34 | 11.78 |
| *ZMO1059* | dihydroneopterin aldolase | 1.10 | 6.12 |
| *ZMO1070* | signal recognition particle-docking protein FtsY | 1.40 | 9.34 |
| *ZMO1071* | MiaB-like tRNA modifying enzyme | 1.46 | 8.61 |
| *ZMO1077* | 16S rRNA processing protein RimM | 1.05 | 5.41 |
| *ZMO1078* | tRNA (guanosine(37)-N1)-methyltransferase TrmD | 1.29 | 8.30 |
| *ZMO1079* | ribosomal protein L19 | 1.68 | 11.42 |
| *ZMO1081* | uncharacterized protein | 1.12 | 9.03 |
| *ZMO1083* | pseudo gene | 1.30 | 8.44 |
| *ZMO1086* | endo-1,4-beta-D-glucanase (Cellulase) | 1.21 | 10.22 |
| *ZMO1090* | dTMP kinase | 1.53 | 9.15 |
| *ZMO1096* | ribonuclease R | 1.32 | 9.57 |
| *ZMO1098* | double-strand break repair helicase AddA | 1.48 | 10.80 |
| *ZMO1099* | double-strand break repair protein AddB | 1.38 | 10.23 |
| *ZMO1100* | nucleotidyl transferase | 1.35 | 10.14 |
| *ZMO1105* | histidine kinase | 2.11 | 11.12 |
| *ZMO1107* | transcriptional regulator AsnC family | 1.67 | 10.00 |
| *ZMO1114* | uracil-DNA glycosylase | 1.45 | 6.57 |
| *ZMO1115* | undecaprenol kinase | 1.24 | 5.43 |
| *ZMO1117* | glutamate synthase large subunit | 1.44 | 7.99 |
| *ZMO1126* | Fis family transcriptional regulator | 1.29 | 5.65 |
| *ZMO1143* | chloramphenicol acetyltransferase | 1.14 | 6.97 |
| *ZMO1145* | ribosomal protein L31 | 1.62 | 8.31 |
| *ZMO1146* | beta-hydroxyacyl-(acyl-carrier-protein) dehydratase FabZ | 1.35 | 11.39 |
| *ZMO1148* | YaeT-like component of outer membrane protein assembly complex | 1.03 | 10.71 |
| *ZMO1149* | membrane-associated Zn-dependent metalloprotease | 1.46 | 11.44 |
| *ZMO1150* | 1-deoxy-D-xylulose 5-phosphate reductoisomerase | 1.86 | 11.46 |
| *ZMO1152* | undecaprenyl pyrophosphate synthase UppS | 1.22 | 10.22 |
| *ZMO1153* | ribosome recycling factor | 1.22 | 10.33 |
| *ZMO1154* | uridylate kinase | 1.88 | 11.79 |
| *ZMO1167* | putative CocE/NonD family hydrolase | 1.13 | 6.87 |
| *ZMO1174* | surface antigen (D15) | 1.05 | 8.90 |
| *ZMO1194* | DNA protecting protein DprA | 1.09 | 6.05 |
| *ZMO1201* | glycine hydroxymethyltransferase | 1.49 | 8.64 |
| *ZMO1202* | transcriptional regulator NrdR | 2.37 | 10.63 |
| *ZMO1203* | TrmH family RNA methyltransferase | 1.23 | 7.33 |
| *ZMO1209* | potassium transporter Kup | 1.96 | 7.84 |
| *ZMO1225* | ribosomal protein S6 | 1.70 | 9.90 |
| *ZMO1226* | ribosomal protein S18 | 2.19 | 8.56 |
| *ZMO1227* | ribosomal protein L9 | 2.14 | 8.88 |
| *ZMO1242* | peptidase M14 carboxypeptidase A | 1.46 | 8.70 |
| *ZMO1243* | DUF4136 domain-containing protein | 1.74 | 6.44 |
| *ZMO1246* | 50S ribosomal protein L36 | 2.80 | 9.60 |
| *ZMO1253* | cytochrome c-type biogenesis protein NrfF/CcmH | 1.65 | 7.66 |
| *ZMO1254* | thiol:disulfide oxidoreductase CcmG | 1.99 | 9.25 |
| *ZMO1255* | cytochrome c-type biogenesis protein CcmF/NfrE | 1.73 | 8.54 |
| *ZMO1257* | hypothetical protein | 1.51 | 5.67 |
| *ZMO1258* | cytochrome C assembly protein CcmC/CycZ | 1.25 | 8.49 |
| *ZMO1267* | GMP synthase large subunit | 1.63 | 7.92 |
| *ZMO1270* | 8-amino-7-oxononanoate synthase | 1.10 | 10.38 |
| *ZMO1276* | uncharacterized protein | 1.01 | 6.69 |
| *ZMO1277* | aminodeoxychorismate lyase | 2.10 | 10.80 |
| *ZMO1279* | acyl carrier protein | 1.97 | 8.91 |
| *ZMO1284* | sorbitol dehydrogenase cytochrome c subunit | 1.06 | 6.55 |
| *ZMO1285* | solbitol dehydrogenase large subunit | 1.73 | 10.53 |
| *ZMO1287* | putative UDP-glucose-glycogen glucosyltransferase | 1.13 | 7.09 |
| *ZMO1294* | N-acetylmuramic acid 6-phosphate etherase MurQ | 1.35 | 8.34 |
| *ZMO1299* | putative capsular polysaccharide biosynthesis protein BcbG | 2.09 | 10.47 |
| *ZMO1302* | lipoate-protein ligase B | 1.07 | 6.00 |
| *ZMO1320* | 16S rRNA C967 or C1407 C5-methylase RsmB/RsmF family | 3.53 | 12.19 |
| *ZMO1321* | inosine-5'-monophosphate dehydrogenase | 2.81 | 12.09 |
| *ZMO1324* | HPr kinase | 1.38 | 9.04 |
| *ZMO1329* | NAD(+) kinase | 1.36 | 6.77 |
| *ZMO1334* | polyisoprenoid-binding periplasmic YceI-like protein | 1.48 | 9.40 |
| *ZMO1345* | aminopeptidase N | 1.15 | 10.00 |
| *ZMO1346* | EamA/RhaT family transporter | 1.18 | 8.67 |
| *ZMO1347* | threonine aldolase | 1.56 | 11.14 |
| *ZMO1360* | pyruvate decarboxylase | 1.71 | 11.36 |
| *ZMO1384* | GTP-binding protein Era | 1.03 | 7.04 |
| *ZMO1389* | Thiol:disulfide interchange protein DsbD | 1.32 | 6.10 |
| *ZMO1393* | LPS export ABC transporter permease LptF | 1.46 | 11.01 |
| *ZMO1394* | LPS export ABC transporter permease LptG | 1.34 | 10.44 |
| *ZMO1403* | peptidase M23 | 3.96 | 14.42 |
| *ZMO1408* | S9 family peptidase | 1.44 | 8.77 |
| *ZMO1420* | 5-(carboxyamino)imidazole ribonucleotide mutase PurE | 1.82 | 9.88 |
| *ZMO1423* | peptidase M16 domain protein | 1.62 | 9.09 |
| *ZMO1430* | MFP subunit of RND efflux system transporter | 1.08 | 6.08 |
| *ZMO1431* | DUF1656 domain-containing protein | 2.21 | 8.88 |
| *ZMO1432* | membrane protein component of efflux system | 2.22 | 10.93 |
| *ZMO1433* | DNA polymerase III delta subunit | 1.77 | 9.78 |
| *ZMO1434* | uncharacterized protein | 2.13 | 10.59 |
| *ZMO1435* | leucyl-tRNA synthetase | 1.24 | 9.65 |
| *ZMO1441* | FAD dependent oxidoreductase | 2.03 | 11.89 |
| *ZMO1444* | glycine--tRNA ligase subunit beta | 1.95 | 11.14 |
| *ZMO1446* | glycine--tRNA ligase subunit alpha | 2.69 | 11.22 |
| *ZMO1451* | purine nucleoside permease | 1.47 | 8.90 |
| *ZMO1452* | MFS family predicted arabinose efflux permease araJ | 2.56 | 10.42 |
| *ZMO1457* | major facilitator superfamily transporter | 3.35 | 8.61 |
| *ZMO1464* | hypothetical protein | 1.79 | 6.37 |
| *ZMO1465* | putative amino acid permease | 2.01 | 7.73 |
| *ZMO1470* | transglycosylase domain-containing protein | 1.51 | 9.19 |
| *ZMO1490* | putative outer membrane associated protein | 1.53 | 10.00 |
| *ZMO1493* | putative membrane protein | 1.02 | 8.05 |
| *ZMO1494* | acetylglutamate kinase | 2.03 | 10.74 |
| *ZMO1496* | phosphoenolpyruvate carboxylase | 1.52 | 9.16 |
| *ZMO1498* | diadenosine tetraphosphate hydrolase | 2.21 | 11.59 |
| *ZMO1500* | imidazole glycerol phosphate synthase cyclase subunit | 1.64 | 9.63 |
| *ZMO1501* | 1-(5-phosphoribosyl)-5-[(5- phosphoribosylamino)methylideneamino] imidazole-4-carboxamide isomerase | 2.30 | 11.89 |
| *ZMO1502* | imidazole glycerol phosphate synthase subunit HisH | 1.79 | 11.21 |
| *ZMO1503* | Imidazoleglycerol-phosphate dehydratase | 1.41 | 7.47 |
| *ZMO1504* | DUF1321 domain-containing protein | 1.10 | 8.52 |
| *ZMO1507* | inorganic diphosphatase | 2.34 | 10.43 |
| *ZMO1508* | histidine--tRNA ligase | 1.55 | 9.17 |
| *ZMO1509* | peptide chain release factor 1 | 1.53 | 10.38 |
| *ZMO1510* | protein-(glutamine-N5) methyltransferase release factor-specific | 2.01 | 11.14 |
| *ZMO1513* | phenylalanine--tRNA ligase subunit beta | 1.28 | 9.84 |
| *ZMO1514* | phenylalanine--tRNA ligase subunit alpha | 1.54 | 9.95 |
| *ZMO1518* | inositol monophosphatase | 3.41 | 13.46 |
| *ZMO1519* | ribose-phosphate pyrophosphokinase | 2.77 | 14.61 |
| *ZMO1528* | multidrug efflux pump subunit AcrB | 1.26 | 7.67 |
| *ZMO1532* | phosphoribosylformylglycinamidine synthase subunit PurQ | 1.23 | 8.36 |
| *ZMO1552* | transcription antitermination factor NusB | 1.11 | 8.24 |
| *ZMO1553* | thiamine-monophosphate kinase | 1.45 | 10.23 |
| *ZMO1561* | short-chain dehydrogenase/reductase SDR | 1.65 | 9.32 |
| *ZMO1562* | RNA pyrophosphohydrolase | 1.67 | 11.42 |
| *ZMO1563* | DUF481 domain-containing protein | 1.19 | 7.32 |
| *ZMO1571* | cytochrome bd ubiquinol oxidase subunit I | 2.78 | 13.22 |
| *ZMO1572* | cytochrome d ubiquinol oxidase subunit II | 2.43 | 12.14 |
| *ZMO1599* | hopanoid biosynthesis associated RND transporter like protein HpnN | 1.93 | 10.57 |
| *ZMO1600* | homoserine kinase | 1.68 | 9.65 |
| *ZMO1601* | ribonuclease H | 2.81 | 9.02 |
| *ZMO1608* | enolase | 1.09 | 6.28 |
| *ZMO1625* | ribonuclease P protein component | 1.67 | 7.34 |
| *ZMO1626* | YidC translocase/secretase | 1.60 | 6.99 |
| *ZMO1633* | uncharacterized protein | 1.54 | 9.15 |
| *ZMO1640* | tryptophanyl-tRNA synthetase | 1.17 | 8.89 |
| *ZMO1649* | gluconolactonase | 1.98 | 11.27 |
| *ZMO1651* | signal transduction protein | 1.80 | 10.20 |
| *ZMO1652* | 2-nitropropane dioxygenase NPD | 1.19 | 6.97 |
| *ZMO1653* | aspartate kinase | 1.13 | 8.24 |
| *ZMO1672* | uncharacterized protein | 1.08 | 4.77 |
| *ZMO1673* | aldo/keto reductase | 1.56 | 10.42 |
| *ZMO1681* | aspartate-alanine antiporter | 1.31 | 6.34 |
| *ZMO1682* | aspartate/methionine/tyrosine aminotransferase | 1.21 | 7.26 |
| *ZMO1683* | type 2 L-asparaginase | 1.36 | 8.33 |
| *ZMO1684* | phosphoserine aminotransferase | 1.38 | 11.13 |
| *ZMO1685* | D-3-phosphoglycerate dehydrogenase | 1.48 | 10.40 |
| *ZMO1686* | ATP phosphoribosyltransferase regulatory subunit HisZ | 2.61 | 12.17 |
| *ZMO1687* | adenylosuccinate synthase | 1.61 | 9.99 |
| *ZMO1707* | orotate phosphoribosyltransferase | 2.60 | 11.23 |
| *ZMO1708* | pyridoxal phosphate biosynthetic protein PdxJ | 1.44 | 8.81 |
| *ZMO1709* | Holo-[acyl-carrier-protein] synthase | 2.44 | 12.76 |
| *ZMO1715* | biopolymer transport protein ExbD/TolR | 1.65 | 10.20 |
| *ZMO1716* | MotA/TolQ/ExbB proton channel | 1.08 | 7.57 |
| *ZMO1717* | TonB family protein | 1.38 | 8.83 |
| *ZMO1718* | uncharacterized protein | 2.03 | 11.92 |
| *ZMO1719* | fructokinase | 1.73 | 10.52 |
| *ZMO1728* | uncharacterized protein | 2.35 | 12.74 |
| *ZMO1740* | DUF3297 domain-containing protein | 1.88 | 12.51 |
| *ZMO1745* | methionine synthase MetH2 | 1.30 | 9.50 |
| *ZMO1746* | 5-methyltetrahydrofolate--homocysteine methyltransferase MetH1 | 1.84 | 10.68 |
| *ZMO1748* | ArsR family transcriptional regulator | 1.36 | 5.73 |
| *ZMO1749* | lipoprotein MlaA | 2.65 | 11.41 |
| *ZMO1755* | thymidylate synthase | 1.41 | 8.53 |
| *ZMO1756* | gluconate transporter GntP | 3.03 | 11.37 |
| *ZMO1757* | gluconate kinase GntK | 1.29 | 9.88 |
| *ZMO1762* | UPF0102 domain-containing protein | 1.00 | 8.79 |
| *ZMO1764* | murein L,D-transpeptidase YcbB/YkuD-like protein | 1.71 | 11.26 |
| *ZMO1768* | diaminopimelate decarboxylase | 1.34 | 10.71 |
| *ZMO1769* | uncharacterized protein | 1.68 | 9.15 |
| *ZMO1777* | uncharacterized protein | 1.17 | 7.17 |
| *ZMO1781* | mechanosensitive ion channel MscS | 1.60 | 7.51 |
| *ZMO1782* | hypothetical protein | 1.00 | 2.52 |
| *ZMO1784* | hypothetical protein | 1.55 | 7.68 |
| *ZMO1785* | mechanosensitive ion channel MscS | 1.80 | 8.40 |
| *ZMO1786* | uncharacterized protein | 1.17 | 7.61 |
| *ZMO1792* | dihydroxy-acid dehydratase | 2.31 | 10.61 |
| *ZMO1793* | LysR family transcriptional regulator | 1.40 | 7.10 |
| *ZMO1794* | uncharacterized protein | 2.21 | 9.60 |
| *ZMO1796* | 3-phosphoshikimate 1-carboxyvinyltransferase | 2.30 | 13.02 |
| *ZMO1802* | hypothetical protein | 4.44 | 13.99 |
| *ZMO1805* | HAD superfamily phosphoglycolate phosphatase | 1.34 | 10.52 |
| *ZMO1818* | 4Fe-4S ferredoxin iron-sulfur binding domain protein | 1.10 | 8.80 |
| *ZMO1845* | PhnA protein | 2.64 | 11.07 |
| *ZMO1860* | putative Ccc1 family vacuolar ion transporter | 1.17 | 8.18 |
| *ZMO1890* | mitochondrial processing peptidase-like protein | 1.10 | 8.28 |
| *ZMO1897* | protein-export membrane protein SecD | 1.03 | 5.69 |
| *ZMO1900* | fatty acid/phospholipid synthesis protein PlsX | 1.62 | 9.06 |
| *ZMO1910* | 50S ribosomal protein L25 | 1.63 | 9.01 |
| *ZMO1911* | peptidyl-tRNA hydrolase | 1.02 | 9.62 |
| *ZMO1925* | sterol-binding peptidase U32 family peptidase | 1.37 | 10.06 |
| *ZMO1932* | uncharacterized protein | 1.56 | 7.80 |
| *ZMO1933* | uncharacterized protein | 1.58 | 8.86 |
| *ZMO1955* | malate dehydrogenase | 1.16 | 9.55 |
| *ZMO1957* | glycosyl transferase family 1 | 1.29 | 10.45 |
| *ZMO1959* | uncharacterized protein | 2.16 | 11.11 |
| *ZMO1962* | histidine kinase | 1.16 | 7.25 |
| *ZMO1963* | citrate synthase I | 2.30 | 11.56 |
| *ZMO1964* | glutamate--tRNA ligase | 2.12 | 12.80 |
| *ZMO1966* | uncharacterized protein | 1.80 | 6.73 |
| *ZMO1976* | uncharacterized protein | 1.97 | 9.17 |
| *ZMO1978* | partition protein ParB | 1.23 | 7.66 |
| *ZMO1980* | 16S rRNA methyltransferase GidB | 1.45 | 8.84 |
| *ZMO1981* | glucose inhibited division protein A GidA | 1.16 | 8.88 |
| *ZMO1996* | transcription termination factor Rho | 1.62 | 6.99 |
| *ZMO2001* | hypothetical protein | 1.34 | 6.07 |
| *ZMO2003* | ribosomal protein S10 | 1.29 | 7.84 |
| *ZMO2012* | acyl carrier protein | 1.05 | 6.77 |
| *ZMO2013* | hypothetical protein | 2.23 | 9.47 |
| *ZMO2017* | capsule biosynthesis phosphatase | 1.73 | 7.09 |
| *ZMO2020* | hypothetical protein | 3.65 | 10.27 |
| *ZMO2021* | DUF2474 domain-containing protein | 1.54 | 6.82 |
| *ZMO2024* | hypothetical protein | 1.96 | 8.49 |
| *ZMO2027* | hypothetical protein | 4.39 | 12.56 |
| *ZMO2031* | ribosomal protein L32 | 1.79 | 10.48 |
| *ZMO2045* | pseudo gene | 1.34 | 3.85 |
| *ZMO2046* | hypothetical protein | 1.52 | 5.08 |
| *ZMO2048* | hypothetical protein | 1.39 | 4.32 |
| *ZMO2049* | hypothetical protein | 1.42 | 7.55 |
| *ZMO2055* | hypothetical protein | 1.76 | 6.33 |
| *ZMO2057* | signal recognition particle sRNA small type | 1.21 | 4.18 |
| *ZMO2066* | hypothetical protein | 3.27 | 11.60 |
| *ZMO2067* | hypothetical protein | 4.84 | 14.15 |
| *ZMO2071* | hypothetical protein | 2.48 | 6.35 |
| *ZMO2073* | hypothetical protein | 1.36 | 3.24 |
| *ZMOp32x028* | Type I restriction endonuclease HsdS | 1.44 | 7.77 |
| *ZMOp36x011* | tail sheath protein | 1.82 | 4.51 |
| *ZMOp36x012* | tail tube protein | 1.60 | 4.35 |
| *ZMOp36x013* | bacteriophage putative tail protein Gp41 | 1.20 | 2.96 |
| *ZMOp36x014* | Burkholderia phage phiE202 Gp27 family protein | 1.73 | 4.76 |
| *ZMOp36x031* | helix-turn-helix XRE super family protein | 1.00 | 4.14 |
| *ZMOp36x038* | uncharacterized protein | 1.12 | 3.03 |
| *ZMOp36x044* | bacteriophage P2 GpN major capsid | 1.26 | 4.18 |
| *ZMOp39x001* | hypothetical protein | 1.37 | 5.53 |
| *ZMOp39x034* | XRE family transcriptional regulator | 1.65 | 7.17 |
| **Downregulated gene in ZM4 cultured at 30℃ compared with 45℃** | | | |
| *ZMO0002* | histidinol-phosphate aminotransferase HisC | -2.94 | 8.23 |
| *ZMO0003* | adenylyl-sulfate kinase | -1.23 | 2.12 |
| *ZMO0010* | DUF45 domain-containing protein | -1.50 | 8.23 |
| *ZMO0015* | heat-inducible transcription repressor HrcA | -5.38 | 13.79 |
| *ZMO0016* | GrpE protein | -4.82 | 13.16 |
| *ZMO0017* | RsmB-like methyltransferase | -3.58 | 12.62 |
| *ZMO0018* | ribulose-phosphate 3-epimerase | -1.17 | 8.17 |
| *ZMO0036* | DUF1491 domain-containing protein | -2.16 | 9.30 |
| *ZMO0037* | PTS IIA-like nitrogen-regulatory protein PtsN/NagE | -2.88 | 10.78 |
| *ZMO0038* | ribosomal protein S30Ae/sigma 54 modulation protein | -3.54 | 10.58 |
| *ZMO0047* | lysine exporter protein (LYSE/YGGA) | -1.08 | 7.15 |
| *ZMO0050* | LysR family transcriptional regulator | -2.10 | 7.42 |
| *ZMO0052* | cyanate permease CynX | -2.12 | 8.91 |
| *ZMO0053* | alpha/beta hydrolase fold protein | -1.91 | 7.77 |
| *ZMO0057* | bacterial PH domain-containing protein | -2.24 | 9.85 |
| *ZMO0062* | aldo/keto reductase | -1.41 | 9.81 |
| *ZMO0070* | GrxB family glutaredoxin 2 | -1.16 | 7.93 |
| *ZMO0089* | hypothetical protein | -1.06 | 6.00 |
| *ZMO0091* | hypothetical protein | -1.86 | 2.61 |
| *ZMO0095* | hypothetical protein | -1.08 | 2.96 |
| *ZMO0101* | NAD-dependent epimerase/dehydratase | -1.31 | 5.40 |
| *ZMO0103* | beta-lactamase | -1.12 | 6.63 |
| *ZMO0105* | 3-isopropylmalate dehydratase large subunit | -2.50 | 12.08 |
| *ZMO0106* | 3-isopropylmalate dehydratase small subunit | -2.13 | 11.32 |
| *ZMO0110* | glycosyl transferase family 2 | -1.34 | 8.27 |
| *ZMO0112* | uncharacterized protein | -4.42 | 12.33 |
| *ZMO0114* | glutamine amidotransferase of anthranilate synthase | -1.09 | 4.99 |
| *ZMO0116* | BadM/Rrf2 family transcriptional regulator | -1.41 | 4.25 |
| *ZMO0117* | hydroxylamine reductase | -1.58 | 5.91 |
| *ZMO0122* | uncharacterized protein | -2.86 | 9.99 |
| *ZMO0125* | YbaK/EbsC-like prolyl-tRNA editing protein | -2.61 | 11.50 |
| *ZMO0127* | S1/P1 nuclease | -1.33 | 7.63 |
| *ZMO0134* | Sel1 domain protein repeat-containing protein | -2.07 | 5.29 |
| *ZMO0135* | Sel1 domain protein repeat-containing protein | -1.15 | 7.34 |
| *ZMO0137* | Sel1 domain protein repeat-containing protein | -1.13 | 8.02 |
| *ZMO0166* | peptidoglycan-associated lipoprotein | -1.11 | 8.34 |
| *ZMO0168* | pseudo gene | -1.22 | 7.93 |
| *ZMO0169* | short-chain dehydrogenase/reductase | -1.81 | 9.43 |
| *ZMO0171* | pseudo gene | -1.29 | 8.06 |
| *ZMO0173* | cell division protein ZapA | -2.27 | 10.31 |
| *ZMO0174* | putative SnoaL-like polynucleotide cyclase | -4.01 | 9.72 |
| *ZMO0179* | fructose-bisphosphate aldolase | -1.75 | 7.75 |
| *ZMO0180* | 1-hydroxy-2-methyl-2-(E)-butenyl 4-diphosphate synthase | -1.83 | 9.11 |
| *ZMO0199* | SOS-response transcriptional repressor LexA | -1.71 | 9.87 |
| *ZMO0203* | MerR family transcriptional regulator | -2.96 | 9.14 |
| *ZMO0204* | cation efflux protein | -1.44 | 10.01 |
| *ZMO0208* | GCN5-related N-acetyltransferase | -3.41 | 11.68 |
| *ZMO0209* | ribosomal protein L27 | -2.08 | 9.21 |
| *ZMO0215* | 5-formyltetrahydrofolate cyclo-ligase | -3.59 | 11.31 |
| *ZMO0216* | peptidase M23 | -1.18 | 8.54 |
| *ZMO0221* | CarD family transcriptional regulator | -3.27 | 10.93 |
| *ZMO0222* | DUF1330 domain-containing protein | -2.54 | 7.59 |
| *ZMO0223* | putative auto-transporter adhesin head GIN domain | -1.36 | 5.33 |
| *ZMO0224* | putative methionine biosynthesis methylase MetW | -2.23 | 9.94 |
| *ZMO0225* | homoserine O-acetyltransferase | -1.25 | 7.92 |
| *ZMO0231* | uncharacterized protein | -3.48 | 12.17 |
| *ZMO0234* | protease Do | -2.06 | 9.29 |
| *ZMO0236* | Mrp family Chromosome partitioning ATPase | -1.79 | 7.60 |
| *ZMO0246* | ATP-dependent protease subunit HslV | -2.06 | 11.16 |
| *ZMO0247* | heat shock protein ATPase subunit HslU | -2.60 | 14.34 |
| *ZMO0252* | major intrinsic protein | -1.15 | 9.06 |
| *ZMO0253* | TolC family type I secretion outer membrane protein | -1.16 | 5.28 |
| *ZMO0257* | winged helix family two component transcriptional regulator | -2.43 | 11.79 |
| *ZMO0263* | uncharacterized protein | -1.48 | 10.36 |
| *ZMO0274* | RNA polymerase factor sigma-54 | -1.06 | 6.65 |
| *ZMO0281* | TetR family transcriptional repressor of RND efflux transport system | -2.49 | 10.66 |
| *ZMO0282* | MFP subunit of RND efflux system transporter | -3.17 | 12.61 |
| *ZMO0283* | hydrophobe/amphiphile efflux-1 (HAE1) family RND efflux system transporter | -1.75 | 9.89 |
| *ZMO0285* | NodT family RND efflux system outer membrane lipoprotein | -1.53 | 9.00 |
| *ZMO0286* | DUF541 domain-containing protein | -2.56 | 9.78 |
| *ZMO0287* | MFP subunit of RND efflux system transporter | -1.70 | 11.14 |
| *ZMO0290* | DUF445 domain-containing protein | -2.82 | 12.97 |
| *ZMO0293* | sugar transporter | -3.51 | 12.48 |
| *ZMO0294* | ribosomal protein L28 | -2.75 | 11.65 |
| *ZMO0298* | uncharacterized protein | -2.62 | 9.63 |
| *ZMO0313* | uncharacterized protein | -1.27 | 6.34 |
| *ZMO0318* | short-chain dehydrogenase/reductase SDR | -1.80 | 8.98 |
| *ZMO0325* | uncharacterized protein | -1.97 | 12.04 |
| *ZMO0347* | RNA chaperone Hfq | -4.10 | 13.99 |
| *ZMO0348* | GTP-binding protein HflX | -1.88 | 10.90 |
| *ZMO0353* | 4-diphosphocytidyl-2C-methyl-D-erythritol synthase | -1.75 | 9.04 |
| *ZMO0354* | DNA mismatch repair protein MutL | -1.36 | 9.40 |
| *ZMO0355* | MreB/Mrl family cell shape determining protein | -2.42 | 13.66 |
| *ZMO0356* | rod shape-determining protein MreC | -1.73 | 10.11 |
| *ZMO0357* | hypothetical protein | -1.04 | 7.87 |
| *ZMO0359* | peptidoglycan D,D-transpeptidase MrdA | -2.04 | 9.28 |
| *ZMO0362* | excinuclease ABC B subunit | -2.32 | 9.94 |
| *ZMO0368* | 6-phosphogluconate dehydratase | -1.44 | 8.86 |
| *ZMO0376* | endopeptidase La | -3.58 | 12.16 |
| *ZMO0385* | hypothetical protein | -3.28 | 8.68 |
| *ZMO0400* | hypothetical protein | -1.17 | 5.94 |
| *ZMO0405* | ATP-dependent Clp protease ATP-binding subunit clpA | -6.51 | 14.17 |
| *ZMO0406* | ABC-2 type transporter | -2.18 | 12.10 |
| *ZMO0407* | GcrA cell cycle regulator | -2.45 | 12.51 |
| *ZMO0422* | BadM/Rrf2 family transcriptional regulator | -1.25 | 7.82 |
| *ZMO0423* | FeS assembly protein SufB | -1.02 | 8.47 |
| *ZMO0427* | SufS subfamily cysteine desulfurase | -1.10 | 9.00 |
| *ZMO0428* | FeS assembly SUF system protein | -3.64 | 10.94 |
| *ZMO0429* | iron-sulfur cluster assembly accessory protein | -1.57 | 9.31 |
| *ZMO0430* | pyrimidine 5'-nucleotidase | -1.46 | 10.33 |
| *ZMO0432* | ureohydrolase-like protein | -1.42 | 8.81 |
| *ZMO0435* | uncharacterized protein | -3.33 | 10.88 |
| *ZMO0443* | ribonucleotide-diphosphate reductase subunit beta | -1.46 | 10.05 |
| *ZMO0469* | hypothetical protein | -4.81 | 13.17 |
| *ZMO0510* | dihydrolipoyllysine-residue acetyltransferase | -1.12 | 7.89 |
| *ZMO0511* | thioesterase superfamily protein | -1.91 | 9.22 |
| *ZMO0552* | thymidine kinase | -1.22 | 8.78 |
| *ZMO0554* | translation initiation factor IF-2 | -1.51 | 9.92 |
| *ZMO0555* | DUF448 domain-containing protein | -1.71 | 11.59 |
| *ZMO0556* | transcription termination protein NusA | -2.97 | 12.69 |
| *ZMO0557* | DUF150/RimP N-terminal domain-containing protein | -3.85 | 13.48 |
| *ZMO0561* | TonB-dependent receptor plug domain | -2.87 | 10.75 |
| *ZMO0562* | histidinol-phosphate aminotransferase HisC | -1.07 | 5.39 |
| *ZMO0569* | succinate dehydrogenase (SQR) cytochrome b subunit | -1.69 | 10.87 |
| *ZMO0570* | ribosomal L11 methyltransferase | -1.73 | 10.78 |
| *ZMO0604* | flagellin domain protein | -1.18 | 6.06 |
| *ZMO0621* | flagellar biosynthesis anti-sigma factor FlgM | -1.81 | 8.72 |
| *ZMO0622* | uncharacterized protein | -1.64 | 7.03 |
| *ZMO0629* | flagellin domain-containing protein | -1.43 | 6.83 |
| *ZMO0651* | flagellar hook protein FliD | -1.08 | 7.22 |
| *ZMO0653* | AI-2E family transporter | -1.08 | 7.08 |
| *ZMO0660* | chaperone protein DnaK | -3.52 | 10.65 |
| *ZMO0661* | chaperone protein DnaJ | -2.41 | 11.47 |
| *ZMO0665* | YnbE family lipoprotein | -2.56 | 8.35 |
| *ZMO0666* | uncharacterized protein | -2.13 | 11.76 |
| *ZMO0672* | excinuclease ABC C subunit | -1.48 | 6.27 |
| *ZMO0693* | OsmC family protein | -1.57 | 8.50 |
| *ZMO0694* | uncharacterized protein | -1.17 | 3.86 |
| *ZMO0698* | putative helix-turn-helix XRE-family like protein | -1.06 | 7.46 |
| *ZMO0699* | hypothetical protein | -2.15 | 8.64 |
| *ZMO0719* | lytic transglycosylase catalytic | -1.24 | 10.59 |
| *ZMO0721* | SsrA-binding protein | -1.67 | 11.75 |
| *ZMO0724* | transcription termination/antitermination factor NusG | -2.31 | 7.96 |
| *ZMO0731* | DNA-directed RNA polymerase beta subunit | -4.12 | 13.06 |
| *ZMO0732* | DNA-directed RNA polymerase beta' subunit | -1.97 | 10.42 |
| *ZMO0733* | GCN5-related N-acetyltransferase | -1.73 | 5.31 |
| *ZMO0740* | general stress protein CsbD | -3.06 | 8.03 |
| *ZMO0748* | cysteine synthase | -3.30 | 9.68 |
| *ZMO0749* | RNA polymerase sigma 32 subunit RpoH | -5.54 | 15.39 |
| *ZMO0750* | 23S rRNA pseudouridine synthase D RluD | -1.82 | 7.74 |
| *ZMO0753* | glutaredoxin 3 | -2.06 | 6.42 |
| *ZMO0754* | sterol-binding domain protein | -1.93 | 7.04 |
| *ZMO0789* | TonB-dependent receptor | -1.17 | 9.89 |
| *ZMO0812* | recombination protein RecR | -1.04 | 7.92 |
| *ZMO0819* | UDP-glucose 6-dehydrogenase | -2.49 | 13.17 |
| *ZMO0885* | hypothetical protein | -3.78 | 9.22 |
| *ZMO0895* | hypothetical protein | -1.53 | 5.05 |
| *ZMO0900* | glutamyl-tRNA synthetase | -1.56 | 12.53 |
| *ZMO0901* | hypothetical protein | -1.13 | 8.69 |
| *ZMO0902* | TonB-dependent receptor | -1.16 | 6.16 |
| *ZMO0903* | 2-isopropylmalate synthase | -3.13 | 11.81 |
| *ZMO0910* | ATPase component of polysaccharide export system | -1.83 | 10.72 |
| *ZMO0915* | copper-translocating P-type ATPase | -3.75 | 12.28 |
| *ZMO0916* | heavy metal transport/detoxification protein | -5.09 | 13.20 |
| *ZMO0917* | 2-nitropropane dioxygenase NPD | -1.67 | 11.37 |
| *ZMO0920* | uncharacterized protein | -2.08 | 7.98 |
| *ZMO0921* | uncharacterized protein | -2.84 | 10.06 |
| *ZMO0922* | helix-turn-helix domain-containing protein | -1.85 | 7.84 |
| *ZMO0935* | glutathione S-transferase domain protein | -1.07 | 6.71 |
| *ZMO0941* | NAD-dependent epimerase/dehydratase | -1.61 | 9.61 |
| *ZMO0948* | ATP-dependent Clp protease proteolytic subunit ClpP | -3.52 | 13.01 |
| *ZMO0949* | ATP-dependent Clp protease ATP-binding subunit ClpX | -3.63 | 16.02 |
| *ZMO0964* | NodT family RND efflux system outer membrane lipoprotein | -3.22 | 12.45 |
| *ZMO0965* | efflux pump membrane protein | -1.89 | 10.58 |
| *ZMO0966* | EmrB/QacA subfamily drug resistance transporter | -1.06 | 6.91 |
| *ZMO0976* | xylose reductase | -1.36 | 9.98 |
| *ZMO0980* | DNA polymerase III beta subunit | -1.20 | 9.99 |
| *ZMO0988* | DUF465 domain-containing protein | -2.33 | 9.55 |
| *ZMO0989* | heat-shock protein IbpA | -8.63 | 15.44 |
| *ZMO0994* | uncharacterized protein | -1.92 | 7.53 |
| *ZMO0996* | mechanosensitive ion channel MscS | -2.02 | 9.05 |
| *ZMO1011* | YacG/DUF329 family protein | -2.02 | 7.06 |
| *ZMO1021* | alpha/beta fold family hydrolase-like protein | -1.38 | 6.90 |
| *ZMO1033* | cyclopropane fatty-acyl-phospholipid synthase Cfa | -1.41 | 8.85 |
| *ZMO1034* | calcium-binding EF-hand-containing protein | -2.44 | 12.22 |
| *ZMO1040* | TonB-dependent receptor | -1.13 | 9.33 |
| *ZMO1041* | NUDIX-like hydrolase | -2.07 | 11.50 |
| *ZMO1043* | LemA family protein | -1.06 | 8.71 |
| *ZMO1045* | phosphate-selective porin O and P | -1.98 | 9.69 |
| *ZMO1054* | DNA topoisomerase IV A subunit | -1.01 | 9.54 |
| *ZMO1060* | superoxide dismutase | -2.12 | 10.68 |
| *ZMO1061* | Fis family sigma54-specific transcriptional acivator | -2.82 | 11.23 |
| *ZMO1062* | putative peripheral inner membrane phage shock protein pspD | -2.33 | 8.10 |
| *ZMO1063* | phage shock protein A PspA | -2.90 | 10.94 |
| *ZMO1064* | phage shock protein B PspB | -2.24 | 9.09 |
| *ZMO1065* | phage shock protein C PspC | -1.70 | 7.23 |
| *ZMO1069* | heat shock protein DnaJ domain protein | -1.45 | 10.47 |
| *ZMO1095* | nucleoside triphosphate pyrophosphohydrolase MazG | -2.77 | 8.61 |
| *ZMO1097* | thioredoxin-domain containing protein | -2.82 | 10.05 |
| *ZMO1113* | FAD-dependent pyridine nucleotide-disulfide oxidoreductase | -1.62 | 8.34 |
| *ZMO1129* | uncharacterized protein | -1.50 | 10.08 |
| *ZMO1130* | CinA domain-containing protein | -1.49 | 9.23 |
| *ZMO1133* | carbonate dehydratase | -2.38 | 10.71 |
| *ZMO1134* | hypothetical protein | -1.11 | 7.74 |
| *ZMO1139* | acetolactate synthase large subunit | -1.26 | 6.82 |
| *ZMO1140* | acetolactate synthase small subunit | -1.36 | 8.04 |
| *ZMO1156* | ribosomal protein S2 | -1.78 | 12.27 |
| *ZMO1158* | aspartate racemase | -2.95 | 10.45 |
| *ZMO1165* | amidophosphoribosyltransferase | -1.10 | 7.94 |
| *ZMO1166* | DNA recombination/repair protein RecA | -3.91 | 12.27 |
| *ZMO1169* | BadF/BadG/BcrA/BcrD type ATPase | -1.17 | 8.97 |
| *ZMO1170* | antibiotic biosynthesis monooxygenase | -2.11 | 11.63 |
| *ZMO1177* | winged helix family two component transcriptional regulator | -1.35 | 11.45 |
| *ZMO1178* | phosphoribosyl-AMP cyclohydrolase | -2.16 | 11.08 |
| *ZMO1187* | formamidopyrimidine-DNA glycosylase | -2.38 | 8.44 |
| *ZMO1205* | hypothetical protein | -2.23 | 9.88 |
| *ZMO1213* | uncharacterized protein | -2.47 | 10.09 |
| *ZMO1218* | twin-arginine translocase subunit TatC | -1.05 | 7.67 |
| *ZMO1219* | twin-arginine translocase subunit TatB | -1.34 | 9.25 |
| *ZMO1220* | twin-arginine translocase subunit TatA | -1.39 | 6.87 |
| *ZMO1221* | uncharacterized protein | -1.75 | 9.49 |
| *ZMO1232* | glycosyl transferase family 1 | -1.42 | 7.70 |
| *ZMO1234* | deoxyxylulose-5-phosphate synthase | -1.30 | 9.22 |
| *ZMO1235* | Fur family ferric uptake regulator | -3.15 | 11.75 |
| *ZMO1236* | Zn-dependent alcohol dehydrogenase | -2.71 | 10.80 |
| *ZMO1237* | lactate dehydrogenase | -2.82 | 9.39 |
| *ZMO1271* | siroheme synthase CysG | -4.29 | 11.34 |
| *ZMO1298* | TonB-dependent receptor plug domain | -1.33 | 8.51 |
| *ZMO1303* | pyrroline-5-carboxylate reductase | -1.71 | 9.58 |
| *ZMO1304* | DUF1790 domain-containing protein | -1.15 | 8.75 |
| *ZMO1312* | PpiC-type peptidyl-prolyl cis-trans isomerase | -1.64 | 8.60 |
| *ZMO1335* | flavoprotein WrbA | -2.49 | 11.19 |
| *ZMO1337* | hydroquinone detoxification protein | -1.07 | 6.41 |
| *ZMO1341* | Zn-dependent metalloprotease | -3.18 | 11.14 |
| *ZMO1344* | 2,5-diketo-D-gluconate reductase A | -1.03 | 8.03 |
| *ZMO1352* | intergral membrane protein | -3.24 | 11.50 |
| *ZMO1366* | 30S ribosomal protein S4 | -1.18 | 9.10 |
| *ZMO1372* | short-chain dehydrogenase/reductase SDR | -2.22 | 11.09 |
| *ZMO1373* | uncharacterized protein | -3.17 | 9.87 |
| *ZMO1374* | NAD-dependent epimerase/dehydratase | -1.35 | 9.42 |
| *ZMO1375* | ribonuclease III | -2.08 | 11.19 |
| *ZMO1381* | aminoglycoside phosphotransferase | -1.23 | 3.87 |
| *ZMO1385* | toxic anion resistance family protein | -1.76 | 9.37 |
| *ZMO1386* | uncharacterized protein | -1.63 | 9.72 |
| *ZMO1387* | winged helix family two component transcriptional regulator | -2.40 | 11.50 |
| *ZMO1390* | uncharacterized protein | -1.82 | 8.21 |
| *ZMO1401* | exodeoxyribonuclease III XthA | -1.64 | 10.13 |
| *ZMO1402* | iron-sulfur cluster assembly accessory protein | -1.55 | 7.74 |
| *ZMO1406* | alpha/beta hydrolase fold protein | -1.47 | 9.73 |
| *ZMO1410* | bacterioferritin | -4.98 | 12.84 |
| *ZMO1414* | tRNA(adenosine(37)-N6)- threonylcarbamoyltransferase complex dimerization subunit type 1 TsaB | -1.15 | 8.52 |
| *ZMO1415* | Nfu/NifU-like scaffold protein | -2.08 | 10.46 |
| *ZMO1424* | ATP-dependent chaperone ClpB | -1.82 | 11.22 |
| *ZMO1426* | DNA repair protein RadC | -1.22 | 6.63 |
| *ZMO1428* | diacylglycerol kinase catalytic region | -1.36 | 8.34 |
| *ZMO1439* | carbon-nitrogen hydrolase | -1.89 | 11.13 |
| *ZMO1440* | uncharacterized protein | -1.85 | 9.60 |
| *ZMO1448* | hypothetical protein | -2.49 | 9.86 |
| *ZMO1449* | mannitol-1-phosphate dehydrogenase | -6.21 | 14.74 |
| *ZMO1459* | nicotinamide mononucleotide transporter PnuC | -1.80 | 5.65 |
| *ZMO1460* | 3-mercaptopyruvate sulfurtransferase | -1.56 | 8.72 |
| *ZMO1463* | TonB-dependent receptor | -1.41 | 4.44 |
| *ZMO1485* | deoxyguanosinetriphosphate triphosphohydrolase | -2.08 | 8.73 |
| *ZMO1489* | 3-deoxy-D-manno-octulosonate cytidylyltransferase | -1.11 | 8.65 |
| *ZMO1506* | uncharacterized protein | -1.45 | 8.99 |
| *ZMO1511* | uncharacterized protein | -1.18 | 9.26 |
| *ZMO1522* | TonB-dependent receptor | -1.01 | 3.88 |
| *ZMO1524* | EamA domain-containing protein | -1.03 | 8.45 |
| *ZMO1533* | hypothetical protein | -3.01 | 12.07 |
| *ZMO1535* | pseudo gene | -1.15 | 7.60 |
| *ZMO1540* | FeoA family protein | -1.96 | 11.53 |
| *ZMO1541* | ferrous iron transport protein B | -1.19 | 8.49 |
| *ZMO1543* | cobalt chelatase subunit CobT | -1.80 | 5.78 |
| *ZMO1544* | cobalt chelatase subunit CobS | -2.46 | 7.00 |
| *ZMO1545* | heat shock protein DnaJ domain protein | -3.57 | 8.05 |
| *ZMO1549* | BolA family protein | -1.54 | 7.79 |
| *ZMO1557* | amidophosphoribosyltransferase | -1.24 | 9.18 |
| *ZMO1566* | UPF0060 domain-containing protein | -1.11 | 5.80 |
| *ZMO1581* | antibiotic biosynthesis monooxygenase | -1.98 | 8.62 |
| *ZMO1582* | uracil-DNA glycosylase-like protein | -2.87 | 11.89 |
| *ZMO1583* | DNA gyrase B subunit | -1.18 | 9.62 |
| *ZMO1584* | DNA replication and repair protein RecF | -1.14 | 5.89 |
| *ZMO1586* | bacterioferritin | -1.46 | 5.72 |
| *ZMO1587* | hypothetical protein | -1.22 | 7.12 |
| *ZMO1588* | excinuclease ABC A subunit | -5.58 | 18.30 |
| *ZMO1589* | toll/interleukin-1 receptor (TIR) domain-containing protein | -2.39 | 9.55 |
| *ZMO1596* | iron-containing alcohol dehydrogenase | -2.94 | 10.16 |
| *ZMO1602* | peptidase | -1.51 | 11.55 |
| *ZMO1603* | putative protein tyrosine phosphatase | -1.08 | 10.03 |
| *ZMO1609* | uncharacterized protein | -1.74 | 9.56 |
| *ZMO1612* | toluene tolerance family protein | -1.51 | 11.48 |
| *ZMO1615* | transcription elongation factor GreA | -1.74 | 10.41 |
| *ZMO1618* | carbamoyl-phosphate synthase small subunit | -1.56 | 8.16 |
| *ZMO1622* | DNA primase | -1.94 | 11.92 |
| *ZMO1623* | RNA polymerase sigma 70 subunit RpoD | -2.41 | 9.71 |
| *ZMO1631* | TonB-dependent siderophore receptor | -1.97 | 8.79 |
| *ZMO1632* | succinyl-diaminopimelate desuccinylase | -1.97 | 11.64 |
| *ZMO1635* | hypothetical protein | -1.54 | 9.55 |
| *ZMO1636* | import inner membrane translocase subunit Tim44 | -2.47 | 12.71 |
| *ZMO1641* | uncharacterized protein | -1.75 | 8.71 |
| *ZMO1659* | ATP-dependent metalloprotease FtsH | -3.13 | 13.37 |
| *ZMO1660* | uncharacterized protein | -2.04 | 12.79 |
| *ZMO1661* | gamma-glutamyl phosphate reductase | -1.28 | 9.04 |
| *ZMO1668* | disulfide bond formation protein DsbB | -1.90 | 11.30 |
| *ZMO1670* | uncharacterized protein | -1.12 | 10.34 |
| *ZMO1671* | uncharacterized protein | -2.47 | 10.95 |
| *ZMO1679* | YgdH family protein | -1.61 | 8.05 |
| *ZMO1690* | chaperone DnaJ domain protein | -4.73 | 10.71 |
| *ZMO1696* | putative NADPH:quinone reductase-related Zn-dependent oxidoreductase | -2.35 | 8.99 |
| *ZMO1697* | HxlR family transcriptional regulator | -4.93 | 8.18 |
| *ZMO1698* | GTP cyclohydrolase II | -2.05 | 8.65 |
| *ZMO1700* | uncharacterized protein | -1.53 | 6.48 |
| *ZMO1703* | ubiquinone biosynthesis hydroxylase UbiH/UbiF/VisC/COQ6 family | -1.06 | 7.17 |
| *ZMO1704* | ATP-dependent protease La (LON) substrate-binding domain | -2.35 | 9.21 |
| *ZMO1705* | thioredoxin domain-containing protein | -3.20 | 10.09 |
| *ZMO1720* | DNA-directed RNA polymerase omega subunit | -3.53 | 12.96 |
| *ZMO1721* | glyoxalase/bleomycin resistance protein dioxygenase | -4.59 | 13.11 |
| *ZMO1732* | alkyl hydroperoxide reductase | -1.53 | 6.53 |
| *ZMO1738* | LytTR family two component transcriptional regulator | -1.78 | 12.53 |
| *ZMO1743* | bis(5'nucleosyl)-tetraphosphatase ApaH | -1.24 | 8.13 |
| *ZMO1753* | ferredoxin--NADP(+) reductase Fpr | -1.05 | 6.02 |
| *ZMO1754* | succinate-semialdehyde dehydrogenase SSADH | -3.95 | 11.33 |
| *ZMO1767* | UTP-glucose-1-phosphate uridylyltransferase | -2.22 | 12.92 |
| *ZMO1776* | aminopeptidase N | -1.97 | 9.64 |
| *ZMO1812* | electron transport complex RnfABCDGE type C subunit | -1.44 | 8.20 |
| *ZMO1813* | electron transport complex RnfABCDGE type B subunit | -2.14 | 9.71 |
| *ZMO1814* | electron transport complex RnfABCDGE type A subunit | -2.76 | 10.23 |
| *ZMO1815* | TonB-dependent siderophore receptor | -1.52 | 6.91 |
| *ZMO1822* | TonB-dependent siderophore receptor | -4.05 | 10.99 |
| *ZMO1823* | nitrogenase reductase iron protein | -1.60 | 6.24 |
| *ZMO1824* | nitrogenase molybdenum-iron protein alpha chain | -1.08 | 4.12 |
| *ZMO1837* | modD protein | -2.92 | 8.47 |
| *ZMO1838* | TOBE domain protein | -3.84 | 9.20 |
| *ZMO1840* | isochorismatase hydrolase | -1.72 | 10.13 |
| *ZMO1841* | positive regulator of sigma E RseC/MucC | -1.07 | 7.48 |
| *ZMO1842* | ApbE family lipoprotein | -1.80 | 8.43 |
| *ZMO1849* | uncharacterized protein | -3.55 | 8.15 |
| *ZMO1850* | hypothetical protein | -3.75 | 10.33 |
| *ZMO1851* | flavodoxin | -4.81 | 11.02 |
| *ZMO1855* | glutamine amidotransferase class-I | -1.63 | 5.45 |
| *ZMO1861* | 2-nitropropane dioxygenase NPD | -3.25 | 11.46 |
| *ZMO1866* | beta-lactamase domain protein | -1.85 | 11.83 |
| *ZMO1867* | Baf family transcriptional acitvator | -2.31 | 12.72 |
| *ZMO1868* | biotin--[acetyl-CoA-carboxylase] ligase | -2.53 | 13.01 |
| *ZMO1871* | quinolinate synthetase complex A subunit | -1.02 | 7.91 |
| *ZMO1872* | uncharacterized protein | -2.83 | 12.79 |
| *ZMO1873* | glutaredoxin-like protein | -4.01 | 12.93 |
| *ZMO1874* | BolA family transcriptional regulator | -2.00 | 10.81 |
| *ZMO1875* | DUF1476 domain-containing protein | -1.89 | 10.29 |
| *ZMO1876* | uncharacterized protein | -1.13 | 6.99 |
| *ZMO1902* | uroporphyrinogen III synthase | -1.54 | 8.75 |
| *ZMO1903* | porphobilinogen deaminase | -3.00 | 9.85 |
| *ZMO1907* | DNA mismatch repair protein MutS | -1.22 | 7.99 |
| *ZMO1909* | DUF177 domain-containing protein | -1.44 | 7.64 |
| *ZMO1918* | adenosylmethionine--8-amino-7-oxononanoate aminotransferase BioA | -1.46 | 8.80 |
| *ZMO1919* | thiol-disulfide isomerase | -1.26 | 9.75 |
| *ZMO1920* | uncharacterized protein | -8.28 | 16.47 |
| *ZMO1921* | primosomal protein N' | -4.31 | 17.37 |
| *ZMO1928* | chaperonin Cpn10 | -6.22 | 13.21 |
| *ZMO1929* | chaperonin GroEL | -6.03 | 12.81 |
| *ZMO1930* | integrase family protein | -2.92 | 9.23 |
| *ZMO1931* | DUF1016 domain-containing protein | -3.88 | 10.09 |
| *ZMO1937* | DNA-binding protein | -1.05 | 2.84 |
| *ZMO1940* | hypothetical protein | -4.87 | 7.45 |
| *ZMO1941* | type IV secretory pathway protease TraF-like protein | -4.79 | 5.57 |
| *ZMO1943* | VirD2 and DUF3363 domain-containing protein | -4.71 | 11.84 |
| *ZMO1944* | GntR transcriptional regulator with aminotransferase domain | -5.19 | 12.46 |
| *ZMO1945* | PhzF-like isomerase | -2.53 | 7.35 |
| *ZMO1946* | short-chain dehydrogenase/reductase SDR | -1.38 | 5.19 |
| *ZMO1947* | RidA (reactive intermediate/imine deaminase A) family protein | -1.45 | 6.96 |
| *ZMO1985* | pseudo gene | -1.39 | 6.08 |
| *ZMO1986* | TonB-dependent receptor plug domain | -1.20 | 7.14 |
| *ZMO1992* | carboxymethylenebutenolidase | -1.10 | 8.58 |
| *ZMO1997* | CopD family protein | -1.00 | 7.82 |
| *ZMO2000* | AcrB/AcrD/AcrF family protein | -4.65 | 13.45 |
| *ZMO2002* | ribosomal protein S12 | -1.36 | 11.10 |
| *ZMO2006* | preprotein translocase SecE subunit | -1.80 | 7.14 |
| *ZMO2010* | pseudo gene | -1.07 | 4.93 |
| *ZMO2011* | entericidin A/B family protein | -3.01 | 9.29 |
| *ZMO2016* | hypothetical protein | -3.18 | 6.29 |
| *ZMO2019* | BFD domain protein (2Fe-2S)-binding domain protein | -6.68 | 11.56 |
| *ZMO2029* | hypothetical protein | -2.60 | 7.27 |
| *ZMO2032* | DNA repair protein RadC | -1.01 | 5.00 |
| *ZMO2033* | XRE family transcriptional regulator | -1.79 | 7.49 |
| *ZMO2034* | DUF2958 domain-containing protein | -2.46 | 6.77 |
| *ZMO2035* | RepA replication protein | -2.61 | 6.30 |
| *ZMO2036* | pseudo gene | -4.59 | 7.38 |
| *ZMO2037* | hypothetical protein | -3.65 | 7.70 |
| *ZMO2041* | Sel1 domain protein repeat-containing protein | -5.13 | 13.31 |
| *ZMO2042* | hypothetical protein | -2.48 | 4.54 |
| *ZMO2044* | redox-sensitive transcriptional activator SoxR | -1.81 | 7.17 |
| *ZMO2053* | uncharacterized protein | -3.75 | 13.08 |
| *ZMO2056* | uncharacterized protein | -4.38 | 12.03 |
| *ZMO2059* | RNase P RNA component class A | -1.89 | 7.82 |
| *ZMO2060* | hypothetical protein | -3.96 | 10.12 |
| *ZMO2062* | hypothetical protein | -1.63 | 6.06 |
| *ZMO2064* | hypothetical protein | -2.18 | 4.27 |
| *ZMO2069* | pseudo gene | -1.05 | 5.14 |
| *ZMO2070* | hypothetical protein | -1.18 | 3.00 |
| *ZMO2074* | hypothetical protein | -1.15 | 2.42 |
| *ZMO2076* | hypothetical protein | -2.02 | 5.91 |
| *ZMO2077* | hypothetical protein | -1.28 | 5.47 |
| *ZMOp32x002* | RelB or DinJ-like antitoxin | -2.49 | 12.69 |
| *ZMOp32x003* | pseudo gene | -2.71 | 10.95 |
| *ZMOp32x004* | P-loop containing nucleoside triphosphate hydrolase domain protein | -2.80 | 11.85 |
| *ZMOp32x005* | uncharacterized protein | -1.85 | 9.54 |
| *ZMOp32x006* | TonB-dependent receptor plug domain protein | -1.67 | 11.27 |
| *ZMOp32x007* | Ca2+-dependent phosphoinositide phospholipase C | -1.42 | 9.12 |
| *ZMOp32x008* | PIN domain protein | -4.51 | 13.44 |
| *ZMOp32x009* | SinI-like DNA-binding domain protein | -5.25 | 13.30 |
| *ZMOp32x010* | Macro or A1pp domain protein | -1.56 | 9.22 |
| *ZMOp32x011* | uncharacterized protein | -2.30 | 8.46 |
| *ZMOp32x012* | hypothetical protein | -3.19 | 8.97 |
| *ZMOp32x013* | hypothetical protein | -2.61 | 9.86 |
| *ZMOp32x014* | hypothetical protein | -2.75 | 10.32 |
| *ZMOp32x015* | Azospirillum phage Cd Gp10 family protein | -2.19 | 6.86 |
| *ZMOp32x019* | hypothetical protein | -1.32 | 3.79 |
| *ZMOp32x020* | MazE antitoxin | -3.70 | 12.60 |
| *ZMOp32x021* | MazF toxin | -3.23 | 14.34 |
| *ZMOp32x022* | SIR2-like protein | -2.89 | 12.36 |
| *ZMOp32x023* | MTH538 TIR-like domain | -1.62 | 5.68 |
| *ZMOp32x024* | phage integrase | -1.89 | 8.17 |
| *ZMOp32x030* | hypothetical protein | -2.66 | 11.42 |
| *ZMOp32x031* | hypothetical protein | -3.02 | 9.73 |
| *ZMOp32x032* | hypothetical protein | -1.29 | 7.87 |
| *ZMOp32x033* | hypothetical protein | -2.02 | 8.83 |
| *ZMOp33x001* | hypothetical protein | -1.54 | 8.53 |
| *ZMOp33x002* | putative Zeta toxin-like kinase | -1.39 | 9.87 |
| *ZMOp33x005* | Vibrio phage ICP1 Orf50 family protein | -1.09 | 5.96 |
| *ZMOp33x006* | acyl-CoA N-acyltransferase | -1.28 | 6.71 |
| *ZMOp33x012* | 2-aminoethylphosphonate--pyruvate transaminase | -1.69 | 9.79 |
| *ZMOp33x013* | DUF3225 family protein with NTF2-like domain | -2.50 | 11.14 |
| *ZMOp33x014* | protein of unknown function DUF4089 | -2.18 | 10.40 |
| *ZMOp33x015* | AtzE type amidohydrolase | -1.13 | 7.60 |
| *ZMOp33x016* | short-chain dehydrogenase/reductase SDR | -1.50 | 8.28 |
| *ZMOp33x018* | bacterial bifunctional deaminase-reductase C-terminal domain-containing protein | -1.24 | 8.73 |
| *ZMOp33x019* | LysR family transcription regulator | -1.31 | 8.22 |
| *ZMOp33x022* | NADP-dependent oxidoreductase domain protein | -1.43 | 8.31 |
| *ZMOp33x023* | NADP-dependent oxidoreductase domain protein | -1.11 | 7.15 |
| *ZMOp33x024* | pseudo gene | -3.58 | 13.06 |
| *ZMOp33x025* | pseudo gene | -1.89 | 10.17 |
| *ZMOp33x026* | toxin RelE-like domain protein | -2.26 | 9.12 |
| *ZMOp33x027* | relaxosome protein TraY | -2.91 | 8.45 |
| *ZMOp33x029* | Azospirillum phage Cd Gp10 family protein | -2.40 | 7.07 |
| *ZMOp33x030* | hypothetical protein | -2.45 | 8.57 |
| *ZMOp33x031* | uncharacterized protein | -3.89 | 10.93 |
| *ZMOp33x032* | hypothetical protein | -4.71 | 9.14 |
| *ZMOp33x033* | hypothetical protein | -2.26 | 7.55 |
| *ZMOp33x036* | ribonuclease toxin BrnT of type II toxin-antitoxin system | -4.68 | 11.71 |
| *ZMOp33x037* | cro/C1-type transcriptional repressor | -4.90 | 14.80 |
| *ZMOp33x038* | Lambda repressor-like transcriptional regulator | -4.30 | 9.90 |
| *ZMOp33x039* | hypothetical protein | -2.39 | 9.57 |
| *ZMOp33x040* | death on curing (Doc) protein | -3.16 | 12.23 |
| *ZMOp36x009* | uncharacterized protein | -1.23 | 4.78 |
| *ZMOp36x016* | phage P2 GpU family protein | -1.13 | 3.25 |
| *ZMOp36x022* | uncharacterized protein | -2.73 | 8.92 |
| *ZMOp36x023* | YafQ-like toxin | -2.83 | 10.55 |
| *ZMOp36x024* | antitoxin DinJ | -3.32 | 10.15 |
| *ZMOp36x028* | uncharacterized protein | -1.33 | 6.33 |
| *ZMOp36x029* | Lambda repressor-like DNA-binding domain protein | -1.94 | 9.39 |
| *ZMOp36x030* | toxin HigB-like | -2.36 | 8.86 |
| *ZMOp36x033* | zinc finger Ogr/Delta-type transcriptional activator | -2.31 | 7.65 |
| *ZMOp36x034* | Azospirillum phage Cd Gp10 family protein | -2.14 | 5.23 |
| *ZMOp36x035* | Azospirillum phage Cd Gp10 family protein | -2.49 | 7.81 |
| *ZMOp36x036* | winged helix-turn-helix DNA-binding domain protein | -1.01 | 4.64 |
| *ZMOp36x041* | PBSX family phage portal protein | -1.58 | 9.37 |
| *ZMOp36x042* | putative ATPase terminase subunit protein | -1.16 | 4.63 |
| *ZMOp36x051* | uncharacterized protein | -1.08 | 5.18 |
| *ZMOp36x052* | hypothetical protein | -1.95 | 9.57 |
| *ZMOp39x004* | uncharacterized protein | -1.27 | 6.39 |
| *ZMOp39x005* | RelB antitoxin/Antitoxin DinJ family toxin | -2.42 | 9.99 |
| *ZMOp39x006* | YafQ-like toxin | -1.92 | 7.54 |
| *ZMOp39x007* | TonB-dependent receptor beta-barrel protein | -1.94 | 10.56 |
| *ZMOp39x009* | putative partitioning protein ParA ATPase | -1.39 | 8.45 |
| *ZMOp39x010* | putative partitioning protein ParB | -1.23 | 6.27 |
| *ZMOp39x019* | pseudo gene | -2.80 | 7.81 |
| *ZMOp39x020* | hypothetical protein | -1.04 | 6.22 |
| *ZMOp39x021* | death on curing (Doc) protein | -1.29 | 7.63 |
| *ZMOp39x022* | helix-turn-helix DUF1870 domain-containing protein | -1.46 | 8.03 |
| *ZMOp39x023* | P-loop containing nucleoside triphosphate hydrolase domain protein | -2.23 | 10.16 |
| *ZMOp39x029* | pseudo gene | -2.01 | 7.18 |
| *ZMOp39x030* | uncharacterized protein | -1.65 | 8.00 |
| *ZMOp39x031* | Alpha/Beta hydrolase fold | -2.51 | 9.80 |
| *ZMOp39x032* | winged helix-turn-helix DNA-binding domain protein | -1.87 | 7.47 |
| *ZMOp39x033* | hypothetical protein | -3.57 | 8.81 |
| *ZMOp39x035* | pseudo gene | -2.94 | 9.35 |
| *ZMOp39x036* | hypothetical protein | -3.79 | 12.36 |
| *ZMOp39x037* | hypothetical protein | -4.21 | 11.34 |

**Table S2_b.** List of significantly differentially expressed genes between ZM4 cultured at 30℃ and 40℃. Ratio is the log_2_-based expression difference between ZM4 cultured at 30℃ and 40℃ (30/40). Gene name with red and blue color font indicates up-regulated and down-regulated at 30℃ compared with 40℃, respectively.

| **Name** | | **Product** | **Ratio** | **-log_10_ (p-value)** |
| --- | --- | --- | --- | --- |
|  | **Upregulated gene in ZM4 cultured at 30℃ compared with 40℃** | | | |
| *ZMO0246* | | ATP-dependent protease subunit HslV | 1.17 | 8.28 |
| *ZMO0374* | | levansucrase | 4.23 | 10.00 |
| *ZMO0375* | | levansucrase/invertase | 2.22 | 6.81 |
| *ZMO0378* | | helix-turn-helix domain-containing protein | 1.05 | 5.22 |
| *ZMO0379* | | PBSX family phage terminase large subunit | 2.61 | 7.67 |
| *ZMO0380* | | hypothetical protein | 1.88 | 7.84 |
| *ZMO0381* | | hypothetical protein | 2.58 | 8.94 |
| *ZMO0383* | | hypothetical protein | 3.49 | 9.29 |
| *ZMO0384* | | hypothetical protein | 3.17 | 6.64 |
| *ZMO0387* | | uncharacterized protein | 2.88 | 8.12 |
| *ZMO0388* | | hypothetical protein | 2.73 | 7.46 |
| *ZMO0389* | | hypothetical protein | 2.21 | 7.02 |
| *ZMO0390* | | hypothetical protein | 2.18 | 7.21 |
| *ZMO0391* | | hypothetical protein | 2.82 | 6.91 |
| *ZMO0392* | | hypothetical protein | 2.43 | 8.51 |
| *ZMO0393* | | hypothetical protein | 1.60 | 5.28 |
| *ZMO0394* | | hypothetical protein | 1.07 | 4.67 |
| *ZMO0395* | | hypothetical protein | 3.52 | 7.73 |
| *ZMO0397* | | hypothetical protein | 3.25 | 7.41 |
| *ZMO0398* | | hypothetical protein | 2.72 | 8.09 |
| *ZMO0399* | | peptidase S74 domain-containing protein | 2.24 | 7.31 |
| *ZMO0400* | | hypothetical protein | 1.09 | 5.59 |
| *ZMO0422* | | BadM/Rrf2 family transcriptional regulator | 1.21 | 7.67 |
| *ZMO0454* | | formate--tetrahydrofolate ligase | 1.13 | 6.98 |
| *ZMO0492* | | nitrogen regulatory protein PII | 1.11 | 6.55 |
| *ZMO0660* | | chaperone protein DnaK | 1.05 | 4.81 |
| *ZMO0693* | | OsmC family protein | 1.13 | 6.89 |
| *ZMO0740* | | general stress protein CsbD | 1.57 | 4.91 |
| *ZMO0930* | | hypothetical protein | 2.49 | 8.44 |
| *ZMO0931* | | hypothetical protein | 3.09 | 9.96 |
| *ZMO0932* | | DUF847/glycoside hydrolase family 108 domain-containing protein | 3.48 | 7.44 |
| *ZMO0934* | | secretion-related protein | 3.05 | 7.38 |
| *ZMO0989* | | heat-shock protein IbpA | 2.77 | 9.58 |
| *ZMO1062* | | putative peripheral inner membrane phage shock protein pspD | 1.82 | 6.88 |
| *ZMO1063* | | phage shock protein A PspA | 1.08 | 6.07 |
| *ZMO1116* | | glutamate synthase small subunit | 1.35 | 7.00 |
| *ZMO1117* | | glutamate synthase large subunit | 1.39 | 7.82 |
| *ZMO1237* | | lactate dehydrogenase | 1.06 | 4.75 |
| *ZMO1283* | | LacI family transcriptional regulator | 1.00 | 6.65 |
| *ZMO1424* | | ATP-dependent chaperone ClpB | 2.09 | 11.93 |
| *ZMO1425* | | thiamine monophosphate synthase | 1.25 | 9.52 |
| *ZMO1426* | | DNA repair protein RadC | 1.04 | 5.91 |
| *ZMO1586* | | bacterioferritin | 1.53 | 5.91 |
| *ZMO1721* | | glyoxalase/bleomycin resistance protein dioxygenase | 1.15 | 6.18 |
| *ZMO1928* | | chaperonin Cpn10 | 2.48 | 8.52 |
| *ZMO1929* | | chaperonin GroEL | 2.32 | 7.96 |
| *ZMOp33x004* | | CobQ/CobB/MinD/ParA nucleotide binding domain protein | 1.06 | 6.22 |
| *ZMOp33x029* | | Azospirillum phage Cd Gp10 family protein | 1.84 | 5.82 |
| *ZMOp33x030* | | hypothetical protein | 1.65 | 6.65 |
| *ZMOp39x009* | | putative partitioning protein ParA ATPase | 1.39 | 8.46 |
| *ZMOp39x010* | | putative partitioning protein ParB | 1.13 | 5.86 |
| *ZMOp39x024* | | hypothetical protein | 1.25 | 4.82 |
| *ZMOp39x025* | | ParG family protein | 1.17 | 6.15 |
| *ZMOp39x026* | | pseudo gene | 1.40 | 7.59 |
|  | **Downregulated gene in ZM4 cultured at 30℃ compared with 40℃** | | | |
| *ZMO0001* | | TonB-dependent receptor | -1.57 | 6.23 |
| *ZMO0003* | | adenylyl-sulfate kinase | -1.50 | 2.68 |
| *ZMO0004* | | sulfate adenylyltransferase large subunit | -2.75 | 4.46 |
| *ZMO0005* | | sulfate adenylyltransferase small subunit | -3.53 | 7.38 |
| *ZMO0006* | | siroheme synthase CysG | -4.04 | 11.94 |
| *ZMO0008* | | sulfite reductase (NADPH) hemoprotein beta-component | -1.34 | 2.65 |
| *ZMO0009* | | sulfite reductase (NADPH) flavoprotein alpha chain | -2.17 | 6.01 |
| *ZMO0055* | | anion permerase | -5.21 | 10.67 |
| *ZMO0747* | | uncharacterized protein | -1.08 | 6.35 |
| *ZMO0748* | | cysteine synthase | -3.51 | 10.01 |
| *ZMO1000* | | 5- methyltetrahydropteroyltriglutamate--homocysteine S-methyltransferase | -1.78 | 8.83 |
| *ZMO1261* | | ABC transporter substrate-binding protein aliphatic sulfonates family SsuA | -2.68 | 6.42 |
| *ZMO1262* | | alkanesulfonate transporter permease subunit SsuC | -2.03 | 4.30 |
| *ZMO1263* | | aliphatic sulfonates transport ATP-binding subunit SsuB | -1.32 | 3.03 |
| *ZMO1271* | | siroheme synthase CysG | -1.08 | 4.66 |
| *ZMO1456* | | LysE family translocator | -1.67 | 6.18 |
| *ZMO1463* | | TonB-dependent receptor | -1.40 | 4.39 |
| *ZMO1475* | | TonB-dependent receptor plug domain | -3.58 | 8.57 |
| *ZMO1719* | | fructokinase | -1.12 | 8.32 |
| *ZMO1747* | | 5,10-methylenetetrahydrofolate reductase | -1.20 | 7.08 |
| *ZMO1748* | | ArsR family transcriptional regulator | -1.54 | 6.31 |
| *ZMO1849* | | uncharacterized protein | -3.16 | 7.58 |
| *ZMO1853* | | dihydrodipicolinate synthetase | -1.23 | 9.30 |
| *ZMO1940* | | hypothetical protein | -1.37 | 2.33 |
| *ZMOp36x043* | | capsid scaffolding protein GpO | -1.40 | 5.14 |
| *ZMOp39x036* | | hypothetical protein | -1.07 | 6.03 |
| *ZMOp39x037* | | hypothetical protein | -1.16 | 5.07 |

**Table S2_c.** List of significantly differentially expressed genes between ZM4 cultured at 30℃ and 36℃. Ratio is the log_2_-based expression difference between ZM4 cultured at 30℃ and 36℃ (30/36). Gene name with red and blue color font indicates up-regulated and down-regulated at 30℃ compared with 36℃, respectively.

| **Name** | **Product** | **Ratio** | **-log_10_(p-value)** |
| --- | --- | --- | --- |
| **Upregulated gene in ZM4 cultured at 30℃ compared with 36℃** | | | |
| *ZMO0384* | hypothetical protein | 1.15 | 2.57 |
| *ZMO0693* | OsmC family protein | 1.33 | 7.69 |
| *ZMO0740* | general stress protein CsbD | 1.74 | 5.37 |
| *ZMO0934* | secretion-related protein | 1.07 | 2.96 |
| *ZMO0989* | heat-shock protein IbpA | 2.06 | 8.10 |
| *ZMO1424* | ATP-dependent chaperone ClpB | 1.72 | 10.92 |
| *ZMO1576* | short-chain dehydrogenase/reductase SDR | 1.02 | 2.69 |
| *ZMO1586* | bacterioferritin | 1.13 | 4.59 |
| *ZMO1721* | glyoxalase/bleomycin resistance protein dioxygenase | 1.02 | 5.64 |
| *ZMO1928* | chaperonin Cpn10 | 1.69 | 6.63 |
| *ZMO1929* | chaperonin GroEL | 1.71 | 6.50 |
| *ZMOp36x001* | N-acetylmuramoyl-L-alanine amidase domain protein | 1.11 | 3.66 |
| *ZMOp36x006* | baseplate assembly protein J | 1.04 | 3.94 |
| *ZMOp36x007* | tail protein I | 1.46 | 4.74 |
| *ZMOp36x010* | phage baseplate assembly protein V/Gp45 | 1.35 | 3.93 |
| *ZMOp36x011* | tail sheath protein | 1.54 | 3.86 |
| *ZMOp36x012* | tail tube protein | 1.39 | 3.79 |
| *ZMOp39x024* | hypothetical protein | 1.04 | 4.09 |
| *ZMOp39x025* | ParG family protein | 1.07 | 5.73 |
| *ZMOp39x026* | pseudo gene | 1.22 | 6.94 |
| **Downregulated gene in ZM4 cultured at 30℃ compared with 36℃** | |  |  |
| *ZMO1000* | 5- methyltetrahydropteroyltriglutamate--homocysteine S-methyltransferase | -1.32 | 7.35 |

**Table S2_d.** List of significantly differentially expressed genes between ZM4 cultured at 24℃ and 30℃. Ratio is the log_2_-based expression difference between ZM4 cultured at 24℃ and 30℃ (24/30). Gene name with red and blue color font indicates up-regulated and down-regulated at 24℃ compared with 30℃, respectively.

| **Name** | **Product** | **Ratio** | **-log10(p-value)** |
| --- | --- | --- | --- |
| **Upregulated gene in ZM4 cultured at 24℃ compared with 30℃** | | | |
| ZMO0122 | uncharacterized protein | 1.16 | 5.60 |
| ZMO0286 | DUF541 domain-containing protein | 1.23 | 6.17 |
| ZMO0693 | OsmC family protein | 1.62 | 8.66 |
| ZMO0740 | general stress protein CsbD | 1.89 | 5.74 |
| ZMO0918 | catalase | 1.21 | 7.63 |
| ZMO1113 | FAD-dependent pyridine nucleotide-disulfide oxidoreductase | 1.07 | 6.34 |
| ZMO1237 | lactate dehydrogenase | 1.60 | 6.60 |
| ZMO1522 | TonB-dependent receptor | 1.30 | 4.89 |
| ZMO1533 | hypothetical protein | 1.10 | 7.01 |
| ZMO1754 | succinate-semialdehyde dehydrogenase SSADH | 1.27 | 5.75 |
| ZMO1776 | aminopeptidase N | 1.14 | 6.90 |
| ZMO1940 | hypothetical protein | 1.87 | 3.32 |

**Table S2_e.** List of significantly differentially expressed genes between ZM4 cultured at 40℃ and 45℃. Ratio is the log_2_-based expression difference between ZM4 cultured at 40℃ and 45℃ (40/45). Gene name with red and blue color font indicates up-regulated and down-regulated at 40℃ compared with 45℃, respectively.

| **Name** | **Product** | **Ratio** | **-log_10_ (p-value)** |
| --- | --- | --- | --- |
| **Upregulated gene in ZM4 cultured at 40℃ compared with 45℃** | | | |
| *ZMO0001* | TonB-dependent receptor | 1.63 | 6.42 |
| *ZMO0004* | sulfate adenylyltransferase large subunit | 3.34 | 5.29 |
| *ZMO0005* | sulfate adenylyltransferase small subunit | 4.88 | 8.98 |
| *ZMO0006* | siroheme synthase CysG | 3.96 | 11.84 |
| *ZMO0007* | phosphoadenosine phosphosulfate reductase | 1.90 | 4.08 |
| *ZMO0008* | sulfite reductase (NADPH) hemoprotein beta-component | 2.65 | 5.24 |
| *ZMO0009* | sulfite reductase (NADPH) flavoprotein alpha chain | 2.89 | 7.38 |
| *ZMO0013* | rdgB/HAM1 family non-canonical purine NTP pyrophosphatase | 1.46 | 8.49 |
| *ZMO0020* | LUD domain-containing protein | 2.12 | 8.89 |
| *ZMO0021* | iron-sulfur cluster-binding protein YkgF | 2.77 | 10.20 |
| *ZMO0022* | Fe-S oxidoreductase | 2.61 | 10.64 |
| *ZMO0023* | AsnC family transcriptional regulator | 1.20 | 8.53 |
| *ZMO0026* | Sel1 domain protein repeat-containing protein | 1.38 | 6.62 |
| *ZMO0027* | bifunctional phosphoribosyl aminoimidazole carboxamide formyltransferase/IMP cyclohydrolase | 2.08 | 11.84 |
| *ZMO0028* | restriction endonuclease | 1.79 | 11.88 |
| *ZMO0030* | glyoxalase/bleomycin resistance protein/dioxygenase | 1.56 | 10.04 |
| *ZMO0040* | dephospho-CoA kinase | 1.15 | 8.36 |
| *ZMO0043* | Maf family protein | 1.22 | 7.20 |
| *ZMO0055* | anion permerase | 4.35 | 9.74 |
| *ZMO0074* | endoribonuclease YbeY | 1.12 | 8.21 |
| *ZMO0080* | chemoreceptor glutamine deamidase CheD | 1.75 | 10.02 |
| *ZMO0081* | chemotaxis signal transduction response regulator CheB | 2.14 | 9.60 |
| *ZMO0082* | CheR-type MCP methyltransferase | 2.07 | 9.48 |
| *ZMO0099* | major facilitator superfamily transporter | 1.10 | 8.02 |
| *ZMO0100* | HxlR family transcriptional regulator | 1.97 | 8.04 |
| *ZMO0130* | acid phosphatase | 1.84 | 10.34 |
| *ZMO0142* | YggS family pyridoxal phosphate enzyme | 1.44 | 8.90 |
| *ZMO0143* | ABC transporter related protein | 1.36 | 8.91 |
| *ZMO0144* | L-aspartate oxidase | 1.02 | 7.77 |
| *ZMO0149* | tRNA (guanine-N(7)-)-methyltransferase | 1.82 | 10.18 |
| *ZMO0152* | pyruvate kinase | 1.40 | 10.42 |
| *ZMO0164* | Tol-Pal cell envelope complex membrane achoring protein TolA | 1.02 | 6.71 |
| *ZMO0175* | uncharacterized protein | 1.23 | 5.04 |
| *ZMO0184* | hypothetical protein | 2.44 | 8.43 |
| *ZMO0190* | RpiR family transcriptional regulator | 2.08 | 11.81 |
| *ZMO0195* | N-acetylmuramoyl-L-alanine amidase | 1.42 | 10.11 |
| *ZMO0200* | anthranilate phosphoribosyltransferase | 1.06 | 8.61 |
| *ZMO0202* | methyl-accepting chemotaxis sensory transducer | 2.20 | 11.65 |
| *ZMO0220* | ferredoxin | 1.26 | 8.03 |
| *ZMO0227* | DNA polymerase I | 1.03 | 8.12 |
| *ZMO0229* | ABC-type transport system permease | 1.16 | 7.18 |
| *ZMO0232* | SpoU type tRNA/rRNA methyltransferase | 1.05 | 8.41 |
| *ZMO0238* | ATP synthase F1 delta subunit | 1.33 | 7.49 |
| *ZMO0239* | ATP synthase F0F1 alpha subunit | 1.75 | 8.96 |
| *ZMO0240* | ATP synthase F0F1 gamma subunit | 1.88 | 8.83 |
| *ZMO0241* | ATP synthase F0F1 beta subunit | 1.32 | 8.08 |
| *ZMO0242* | ATP synthase F0F1 epsilon subunit | 1.33 | 8.29 |
| *ZMO0243* | uncharacterized protein | 1.00 | 9.13 |
| *ZMO0244* | HU family DNA-binding protein | 1.84 | 8.99 |
| *ZMO0265* | uncharacterized protein | 1.15 | 8.61 |
| *ZMO0270* | uncharacterized protein | 1.60 | 6.94 |
| *ZMO0271* | apolipoprotein N-acyltransferase | 1.29 | 9.68 |
| *ZMO0276* | OstA family protein | 2.14 | 11.18 |
| *ZMO0278* | ribonuclease D | 2.45 | 13.09 |
| *ZMO0296* | tRNA-specific adenosine deaminase | 1.22 | 5.14 |
| *ZMO0305* | stress responsive alpha-beta barrel domain protein | 1.13 | 8.33 |
| *ZMO0306* | glycosyl transferase group 1 | 1.08 | 5.90 |
| *ZMO0307* | capsule polysaccharide biosynthesis protein | 1.29 | 7.13 |
| *ZMO0321* | dihydrofolate reductase region | 1.45 | 9.80 |
| *ZMO0322* | riboflavin biosynthesis protein RibF | 1.37 | 6.07 |
| *ZMO0329* | inositol monophosphatase | 1.35 | 9.34 |
| *ZMO0331* | DUF882 domain-containing protein | 1.86 | 10.37 |
| *ZMO0345* | endonuclease/exonuclease/phosphatase | 1.33 | 9.93 |
| *ZMO0366* | glucose facilitated diffusion protein | 2.43 | 13.89 |
| *ZMO0372* | AsnC family transcriptional regulator | 1.29 | 5.00 |
| *ZMO0412* | multiple antibiotic resistance (MarC)-related protein | 1.14 | 8.80 |
| *ZMO0413* | YggT family protein | 1.05 | 6.93 |
| *ZMO0442* | HAD (haloacid dehalogenase) family phosphatase | 1.07 | 8.25 |
| *ZMO0445* | GDSL lipase/esterase | 1.53 | 10.11 |
| *ZMO0446* | membrane bound O-acyl transferase MBOAT family protein | 2.31 | 11.96 |
| *ZMO0447* | hypothetical protein | 1.15 | 7.05 |
| *ZMO0454* | formate--tetrahydrofolate ligase | 1.26 | 7.49 |
| *ZMO0456* | ferredoxin | 1.81 | 11.73 |
| *ZMO0457* | glutamate uptake regulatory protein | 1.12 | 9.19 |
| *ZMO0458* | lipoprotein-releasing system ATP-binding protein LolD | 1.56 | 9.38 |
| *ZMO0459* | lipoprotein releasing system transmembrane protein LolC/E family | 1.61 | 11.85 |
| *ZMO0460* | proline--tRNA ligase | 1.38 | 10.26 |
| *ZMO0462* | CTP synthase | 1.61 | 10.13 |
| *ZMO0464* | preprotein translocase SecG subunit | 1.34 | 8.17 |
| *ZMO0467* | PpiC-type peptidyl-prolyl cis-trans isomerase | 1.29 | 8.24 |
| *ZMO0476* | riboflavin biosynthesis protein RibD | 1.34 | 8.58 |
| *ZMO0477* | uncharacterized protein | 1.31 | 8.12 |
| *ZMO0480* | histidine kinase | 1.06 | 9.75 |
| *ZMO0481* | uncharacterized protein | 1.79 | 10.54 |
| *ZMO0483* | homoserine dehydrogenase | 1.68 | 12.47 |
| *ZMO0492* | nitrogen regulatory protein PII | 2.05 | 9.57 |
| *ZMO0493* | type I glutamine synthetase | 1.83 | 9.65 |
| *ZMO0497* | phosphoglycolate phosphatase | 1.27 | 7.37 |
| *ZMO0500* | uncharacterized protein | 1.60 | 9.88 |
| *ZMO0501* | polysaccharide synthesis protein GtrA | 1.14 | 7.60 |
| *ZMO0502* | uncharacterized protein | 1.37 | 10.82 |
| *ZMO0503* | glycosyl transferase family 2 | 2.30 | 11.45 |
| *ZMO0505* | pseudouridine synthase | 1.22 | 7.96 |
| *ZMO0506* | peptide chain release factor 2 | 1.17 | 4.60 |
| *ZMO0509* | uncharacterized protein | 1.06 | 7.29 |
| *ZMO0525* | ribosomal protein S17 | 1.09 | 7.92 |
| *ZMO0526* | ribosomal protein L14 | 1.13 | 8.40 |
| *ZMO0527* | ribosomal protein L24 | 1.05 | 7.59 |
| *ZMO0528* | ribosomal protein L5 | 1.05 | 8.42 |
| *ZMO0529* | ribosomal protein S14 | 1.12 | 9.35 |
| *ZMO0530* | ribosomal protein S8 | 1.25 | 8.82 |
| *ZMO0531* | ribosomal protein L6 signature 1 | 1.56 | 9.59 |
| *ZMO0532* | ribosomal protein L18 | 1.37 | 7.59 |
| *ZMO0533* | ribosomal protein S5 | 1.33 | 7.91 |
| *ZMO0534* | ribosomal protein L30 | 1.68 | 8.21 |
| *ZMO0537* | preprotein translocase SecY subunit | 1.81 | 11.65 |
| *ZMO0543* | aconitate hydratase | 1.40 | 8.33 |
| *ZMO0545* | bifunctional N-(5-phosphoribosyl)anthranilate isomerase/indole-3-glycerolphosphate synthase | 1.49 | 7.69 |
| *ZMO0548* | signal transduction histidine kinase | 2.64 | 10.19 |
| *ZMO0549* | polyribonucleotide nucleotidyltransferase | 2.09 | 11.88 |
| *ZMO0568* | succinate dehydrogenase (SQR) hydrophobic subunit | 1.06 | 8.38 |
| *ZMO0573* | glutaredoxin 2 GrxB family | 1.23 | 6.77 |
| *ZMO0575* | RsmD family protein | 1.51 | 9.17 |
| *ZMO0576* | pseudouridine synthase | 1.34 | 9.57 |
| *ZMO0578* | sodium:dicarboxylate symporter | 1.33 | 5.35 |
| *ZMO0581* | major facilitator superfamily transporter | 1.13 | 8.57 |
| *ZMO0582* | bifunctional folylpolyglutamate synthase/dihydrofolate synthase FolC | 1.02 | 9.25 |
| *ZMO0583* | acetyl-CoA carboxylase beta subunit | 1.26 | 10.27 |
| *ZMO0584* | tryptophan synthase alpha subunit | 2.32 | 12.15 |
| *ZMO0585* | tryptophan synthase beta subunit | 1.51 | 9.84 |
| *ZMO0586* | phosphoribosylanthranilate isomerase | 1.25 | 7.12 |
| *ZMO0587* | orotidine 5'-phosphate decarboxylase | 1.19 | 5.91 |
| *ZMO0602* | flagellar motor protein MotB | 2.32 | 10.87 |
| *ZMO0603* | flagellar motor protein MotA | 2.55 | 14.29 |
| *ZMO0605* | flagellar hook-associated protein FlgK | 1.67 | 9.38 |
| *ZMO0606* | flagellar rod assembly protein/muramidase FlgJ | 2.84 | 13.00 |
| *ZMO0607* | flagellar P-ring protein | 2.87 | 13.97 |
| *ZMO0608* | flagellar L-ring protein | 2.52 | 11.65 |
| *ZMO0609* | flagellar basal-body rod protein FlgG | 2.86 | 13.31 |
| *ZMO0610* | flagellar basal-body rod protein FlgF | 3.20 | 14.03 |
| *ZMO0611* | flagellar hook protein FlgE | 3.63 | 15.04 |
| *ZMO0612* | flagellar hook capping protein | 3.70 | 13.20 |
| *ZMO0613* | flagellar basal-body rod protein FlgC | 3.28 | 12.75 |
| *ZMO0614* | flagellar basal-body rod protein FlgB | 4.38 | 12.70 |
| *ZMO0624* | flagellar biosynthesis protein FlhA | 1.75 | 11.28 |
| *ZMO0625* | MinD-like ATPase FlhG | 1.17 | 7.87 |
| *ZMO0626* | RNA polymerase sigma 28 subunit FliA/WhiG | 1.95 | 8.81 |
| *ZMO0627* | GT2 family glycosyltransferase | 2.47 | 11.76 |
| *ZMO0631* | Fis family sigma54-specific transcriptional regulator | 3.63 | 12.08 |
| *ZMO0632* | flagellar hook-basal body complex subunit FliE | 3.12 | 12.42 |
| *ZMO0634* | flagellar M-ring protein FliF | 2.99 | 12.46 |
| *ZMO0635* | flagellar motor switch protein FliG | 3.90 | 11.76 |
| *ZMO0636* | negative regulator of FliI ATPase | 2.13 | 11.55 |
| *ZMO0637* | flagellum-specific ATP synthase FliI | 1.67 | 11.53 |
| *ZMO0641* | pseudo gene | 1.63 | 9.02 |
| *ZMO0642* | flagellar basal body-associated protein FliL | 1.96 | 11.43 |
| *ZMO0643* | flagellar motor switch protein FliM | 1.16 | 6.01 |
| *ZMO0644* | flagellar motor switch protein FliN | 1.09 | 6.22 |
| *ZMO0646* | flagellar biosynthetic protein FliO | 1.56 | 7.74 |
| *ZMO0647* | flagellar biosynthesis protein FliP | 1.40 | 7.40 |
| *ZMO0654* | polysaccharide deacetylase | 1.09 | 6.29 |
| *ZMO0655* | adenosine/AMP deaminase | 1.19 | 8.68 |
| *ZMO0662* | adenylosuccinate lyase | 1.53 | 10.47 |
| *ZMO0663* | DNA repair protein RadC | 1.00 | 5.53 |
| *ZMO0667* | ATP synthase F0F1 A subunit | 1.20 | 8.75 |
| *ZMO0668* | ATP synthase F0F1 C subunit | 1.28 | 8.88 |
| *ZMO0669* | ATP synthase F0F1 B/B' subunit | 2.03 | 9.82 |
| *ZMO0677* | 3-isopropylmalate dehydrogenase | 1.40 | 8.28 |
| *ZMO0686* | signal transduction protein | 1.26 | 4.05 |
| *ZMO0687* | acetolactate synthase large subunit | 2.47 | 8.01 |
| *ZMO0689* | glucose-fructose oxidoreductase | 1.61 | 8.37 |
| *ZMO0691* | tRNA (uracil-5-)-methyltransferase Gid | 1.20 | 10.07 |
| *ZMO0704* | AMP-dependent synthetase and ligase | 1.34 | 10.34 |
| *ZMO0707* | dihydrodipicolinate reductase | 1.02 | 7.40 |
| *ZMO0708* | phosphoribosylglycinamide formyltransferase | 1.22 | 8.22 |
| *ZMO0715* | aspartate--tRNA(Asp/Asn) ligase | 1.33 | 8.41 |
| *ZMO0716* | DUF344/polyphosphate kinase 2 domain-containing protein | 1.18 | 8.26 |
| *ZMO0735* | acetyl-CoA carboxylase complex biotin carboxylase subunit | 2.05 | 11.07 |
| *ZMO0736* | acetyl-CoA carboxylase complex biotin carboxyl carrier protein | 2.14 | 11.95 |
| *ZMO0738* | thiazole biosynthesis protein ThiG | 1.11 | 7.28 |
| *ZMO0743* | peptide chain release factor 3 | 2.07 | 10.66 |
| *ZMO0744* | uncharacterized protein | 1.74 | 8.18 |
| *ZMO0747* | uncharacterized protein | 1.11 | 6.48 |
| *ZMO0751* | Mov34/MPN/PAD-1 family protein | 1.65 | 10.02 |
| *ZMO0752* | histidine phosphotransferase | 1.58 | 10.26 |
| *ZMO0756* | TPR repeat-containing protein | 2.18 | 10.93 |
| *ZMO0757* | TPR repeat-containing protein | 1.81 | 8.79 |
| *ZMO0758* | isochorismatase hydrolase | 1.98 | 11.48 |
| *ZMO0759* | hydroxyacylglutathione hydrolase | 1.35 | 10.14 |
| *ZMO0765* | threonyl-tRNA synthetase | 1.92 | 11.18 |
| *ZMO0766* | bifunctional uridylyltransferase/uridylyl-removing protein GlnD | 1.08 | 8.82 |
| *ZMO0767* | uncharacterized protein | 1.65 | 10.83 |
| *ZMO0768* | hypothetical protein | 1.33 | 7.08 |
| *ZMO0769* | tRNA 2-thiouridine(34) synthase MnmA | 1.32 | 7.92 |
| *ZMO0770* | magnesium transporter | 1.70 | 12.11 |
| *ZMO0773* | peptidyl-prolyl cis-trans isomerase cyclophilin type | 1.93 | 8.67 |
| *ZMO0778* | inner membrane transporter of efflux system | 1.29 | 9.51 |
| *ZMO0779* | RND efflux pump membrane fusion protein | 1.83 | 9.87 |
| *ZMO0780* | AcrB-like multidrug efflux transporter | 2.79 | 11.88 |
| *ZMO0782* | glutamyl-tRNA(Gln) amidotransferase B subunit | 2.77 | 13.44 |
| *ZMO0783* | glutamyl-tRNA(Gln) amidotransferase A subunit | 3.36 | 13.36 |
| *ZMO0784* | glutamyl-tRNA(Gln) amidotransferase C subunit | 3.34 | 13.36 |
| *ZMO0785* | Holliday junction resolvase YqgF | 1.42 | 6.98 |
| *ZMO0791* | aspartate carbamoyltransferase | 1.52 | 9.49 |
| *ZMO0793* | uncharacterized protein | 1.18 | 4.91 |
| *ZMO0797* | short-chain dehydrogenase/reductase SDR | 1.77 | 8.35 |
| *ZMO0799* | ABC-2 type transporter | 1.05 | 5.53 |
| *ZMO0800* | ABC transporter related protein | 1.27 | 7.11 |
| *ZMO0801* | secretion protein HlyD family protein | 2.15 | 7.88 |
| *ZMO0803* | sporulation domain protein | 1.94 | 10.25 |
| *ZMO0804* | N-acetyl-gamma-glutamyl-phosphate reductase | 1.74 | 8.87 |
| *ZMO0806* | cytosol aminopeptidase | 2.33 | 11.58 |
| *ZMO0807* | ribosomal protein S12 methylthiotransferase RimO | 2.85 | 13.43 |
| *ZMO0820* | phosphoribosylformylglycinamidine synthase subunit PurL | 1.52 | 10.49 |
| *ZMO0822* | uncharacterized protein | 1.13 | 6.56 |
| *ZMO0825* | penicillin-binding protein PBP2 | 1.74 | 11.65 |
| *ZMO0826* | UDP-N-acetylmuramoyl-L-alanyl-D-glutamate-2, 6-diaminopimelate ligase | 2.33 | 13.68 |
| *ZMO0827* | UDP-N-acetylmuramoyl-tripeptide-D-alanyl-D- alanine ligase | 2.80 | 14.52 |
| *ZMO0828* | phospho-N-acetylmuramoyl-pentapeptide transferase | 3.55 | 13.79 |
| *ZMO0829* | UDP-N-acetylmuramoyl-L-alanine-D-glutamate ligase | 3.06 | 14.24 |
| *ZMO0830* | cell division protein FtsW | 2.55 | 12.76 |
| *ZMO0831* | undecaprenyldiphospho-muramoylpentapeptide beta-N-acetylglucosaminyltransferase | 2.52 | 13.28 |
| *ZMO0832* | UDP-N-acetylmuramate-L-alanine ligase | 2.42 | 11.63 |
| *ZMO0833* | UDP-N-acetylenolpyruvoylglucosamine reductase | 3.03 | 13.33 |
| *ZMO0834* | D-alanine-D-alanine ligase | 2.76 | 13.99 |
| *ZMO0835* | cell division protein FtsQ | 2.56 | 12.33 |
| *ZMO0840* | sporulation domain-containing protein | 1.57 | 9.19 |
| *ZMO0841* | chloride channel core | 2.29 | 9.72 |
| *ZMO0845* | alanine--tRNA ligase | 1.80 | 12.19 |
| *ZMO0847* | carbohydrate-selective porin OprB | 1.50 | 10.00 |
| *ZMO0850* | Fis family sigma54-specific transcriptional regulator | 1.07 | 8.91 |
| *ZMO0852* | tRNA preQ1(34) S-adenosylmethionine ribosyltransferase-isomerase QueA | 1.80 | 10.38 |
| *ZMO0853* | peptidyl-prolyl cis-trans isomerase cyclophilin type | 1.08 | 8.64 |
| *ZMO0854* | pantetheine-phosphate adenylyltransferase | 1.86 | 12.37 |
| *ZMO0855* | geranylgeranyl pyrophosphate synthase | 2.65 | 13.37 |
| *ZMO0856* | exodeoxyribonuclease VII small subunit | 1.85 | 11.73 |
| *ZMO0857* | DUF1013 domain-containing protein | 2.86 | 10.32 |
| *ZMO0859* | aminotransferase class V | 2.26 | 10.47 |
| *ZMO0861* | DNA polymerase III subunits gamma and tau | 1.24 | 6.59 |
| *ZMO0864* | cytidine deaminase | 1.24 | 10.70 |
| *ZMO0866* | cation diffusion facilitator family transporter | 4.12 | 12.43 |
| *ZMO0867* | hopanoid-associated sugar epimerase HpnA | 2.41 | 13.36 |
| *ZMO0868* | hopene-associated glycosyltransferase HpnB | 2.14 | 13.20 |
| *ZMO0869* | squalene synthase HpnC | 2.06 | 13.01 |
| *ZMO0870* | squalene synthase HpnD | 1.68 | 10.75 |
| *ZMO0871* | squalene-associated FAD-dependent desaturase HpnE | 1.96 | 12.57 |
| *ZMO0872* | squalene-hopene cyclase | 2.27 | 12.69 |
| *ZMO0873* | hopanoid-associated phosphorylase | 1.62 | 9.23 |
| *ZMO0874* | hopanoid biosynthesis associated radical SAM protein HpnH | 1.20 | 9.49 |
| *ZMO0883* | ribosomal protein S9 | 2.15 | 10.57 |
| *ZMO0884* | ribosomal protein L13 | 2.73 | 12.02 |
| *ZMO0886* | DUF192 domain-containing protein | 1.30 | 6.42 |
| *ZMO0893* | beta-lactamase | 1.14 | 7.17 |
| *ZMO0896* | uncharacterized protein | 1.89 | 12.53 |
| *ZMO0919* | diguanylate cyclase with beta propeller sensor | 1.03 | 6.86 |
| *ZMO0936* | EamA/RhaT family transporter | 1.73 | 9.28 |
| *ZMO0937* | aromatic-amino-acid transaminase | 1.18 | 8.64 |
| *ZMO0946* | trigger factor | 1.45 | 9.18 |
| *ZMO0947* | glycosyl transferase family 2 | 2.16 | 11.88 |
| *ZMO0951* | coproporphyrinogen oxidase | 1.20 | 6.36 |
| *ZMO0952* | SpoU type tRNA/rRNA methyltransferase | 1.45 | 7.07 |
| *ZMO0956* | ubiquinol-cytochrome c reductase iron-sulfur subunit | 1.07 | 6.13 |
| *ZMO0957* | cytochrome b | 1.19 | 10.87 |
| *ZMO0962* | N-acetylglucosamine-6-phosphate deacetylase | 1.14 | 5.76 |
| *ZMO0969* | xanthine/uracil/vitamin C permease | 1.93 | 11.10 |
| *ZMO0970* | purine nucleoside permease | 1.54 | 9.78 |
| *ZMO0971* | adenosine deaminase | 1.15 | 9.49 |
| *ZMO0972* | hopanoid biosynthesis associated glycosyl transferase protein HpnI | 1.36 | 9.57 |
| *ZMO0973* | hopanoid biosynthesis associated radical SAM protein HpnJ | 1.17 | 10.27 |
| *ZMO0975* | putative membrane spanning protein | 2.38 | 11.78 |
| *ZMO0979* | TonB-dependent receptor | 1.15 | 7.20 |
| *ZMO0981* | ABC transporter ATP-binding protein | 1.44 | 8.52 |
| *ZMO0982* | ABC transporter permease | 1.72 | 7.67 |
| *ZMO0983* | type 1 ABC transporter solute-binding subunit | 1.01 | 8.47 |
| *ZMO0984* | lipoprotein NlpD | 2.16 | 11.21 |
| *ZMO0985* | 5'-nucleotidase SurE | 3.31 | 14.21 |
| *ZMO0986* | seryl-tRNA synthetase | 2.79 | 14.77 |
| *ZMO0999* | L-threonylcarbamoyladenylate synthase | 1.15 | 8.02 |
| *ZMO1000* | 5- methyltetrahydropteroyltriglutamate--homocysteine S-methyltransferase | 1.48 | 7.90 |
| *ZMO1001* | amino acid/peptide transporter | 1.01 | 6.36 |
| *ZMO1003* | bifunctional hydroxymethylpyrimidine/phosphomethylpyrimidine kinase | 1.26 | 10.08 |
| *ZMO1012* | uncharacterized protein | 1.61 | 9.39 |
| *ZMO1013* | septum formation inhibitor Maf | 2.93 | 10.72 |
| *ZMO1014* | translation initiation factor IF-1 | 3.04 | 11.10 |
| *ZMO1015* | DUF330 domain-containing protein | 1.29 | 8.05 |
| *ZMO1016* | MCE family protein | 1.66 | 10.51 |
| *ZMO1017* | iron ABC transporter ATP-binding protein | 1.98 | 9.89 |
| *ZMO1018* | DUF140 domain-containing protein | 2.03 | 13.73 |
| *ZMO1019* | deoxyhypusine synthase | 1.72 | 10.02 |
| *ZMO1020* | diaminopimelate decarboxylase LysA | 1.54 | 9.80 |
| *ZMO1025* | anaerobic ribonucleoside-triphosphate reductase | 1.13 | 6.11 |
| *ZMO1026* | uncharacterized protein | 1.02 | 6.46 |
| *ZMO1027* | pyruvate-formate lyase-activating enzyme RflA | 1.64 | 8.80 |
| *ZMO1036* | argininosuccinate synthase ArgG | 1.36 | 10.05 |
| *ZMO1039* | ribonucleoside-diphosphate reductase alpha subunit | 1.36 | 11.21 |
| *ZMO1055* | bifunctional diguanylate cyclase (GGDEF)/phosphodiesterase | 1.15 | 6.86 |
| *ZMO1056* | polynucleotide adenylyltransferase | 1.76 | 9.04 |
| *ZMO1057* | GCN5-related N-acetyltransferase | 2.52 | 12.16 |
| *ZMO1059* | dihydroneopterin aldolase | 1.36 | 7.14 |
| *ZMO1070* | signal recognition particle-docking protein FtsY | 1.69 | 10.31 |
| *ZMO1071* | MiaB-like tRNA modifying enzyme | 1.71 | 9.38 |
| *ZMO1072* | diaminopimelate epimerase | 1.18 | 7.05 |
| *ZMO1077* | 16S rRNA processing protein RimM | 1.14 | 5.77 |
| *ZMO1078* | tRNA (guanosine(37)-N1)-methyltransferase TrmD | 1.49 | 9.02 |
| *ZMO1079* | ribosomal protein L19 | 1.75 | 11.64 |
| *ZMO1081* | uncharacterized protein | 1.12 | 9.00 |
| *ZMO1083* | pseudo gene | 1.44 | 8.97 |
| *ZMO1090* | dTMP kinase | 1.76 | 9.86 |
| *ZMO1091* | DNA polymerase III subunit delta' | 1.12 | 10.29 |
| *ZMO1092* | methionine--tRNA ligase | 1.14 | 10.92 |
| *ZMO1094* | metallo-beta-lactamase-like protein | 1.05 | 8.08 |
| *ZMO1096* | ribonuclease R | 1.25 | 9.26 |
| *ZMO1098* | double-strand break repair helicase AddA | 1.76 | 11.70 |
| *ZMO1099* | double-strand break repair protein AddB | 1.54 | 10.81 |
| *ZMO1100* | nucleotidyl transferase | 1.54 | 10.81 |
| *ZMO1105* | histidine kinase | 2.51 | 12.02 |
| *ZMO1107* | transcriptional regulator AsnC family | 1.62 | 9.86 |
| *ZMO1114* | uracil-DNA glycosylase | 1.78 | 7.55 |
| *ZMO1115* | undecaprenol kinase | 1.10 | 4.92 |
| *ZMO1126* | Fis family transcriptional regulator | 1.68 | 6.88 |
| *ZMO1143* | chloramphenicol acetyltransferase | 1.30 | 7.63 |
| *ZMO1144* | teicoplanin resistance protein VanZ | 1.16 | 7.82 |
| *ZMO1145* | ribosomal protein L31 | 1.25 | 7.05 |
| *ZMO1146* | beta-hydroxyacyl-(acyl-carrier-protein) dehydratase FabZ | 1.47 | 11.82 |
| *ZMO1147* | chaperone protein Skp (OmpH) | 1.11 | 8.90 |
| *ZMO1148* | YaeT-like component of outer membrane protein assembly complex | 1.00 | 10.57 |
| *ZMO1149* | membrane-associated Zn-dependent metalloprotease | 1.63 | 12.02 |
| *ZMO1150* | 1-deoxy-D-xylulose 5-phosphate reductoisomerase | 2.07 | 12.01 |
| *ZMO1151* | phosphatidate cytidylyltransferase | 1.01 | 6.09 |
| *ZMO1152* | undecaprenyl pyrophosphate synthase UppS | 1.40 | 10.93 |
| *ZMO1153* | ribosome recycling factor | 1.39 | 10.98 |
| *ZMO1154* | uridylate kinase | 2.05 | 12.24 |
| *ZMO1159* | CDP-diacylglycerol--serine O-phosphatidyltransferase | 1.05 | 6.17 |
| *ZMO1167* | putative CocE/NonD family hydrolase | 1.13 | 6.84 |
| *ZMO1174* | surface antigen (D15) | 1.16 | 9.42 |
| *ZMO1190* | bifunctional phosphopantothenoylcysteine decarboxylase/phosphopantothenate--cysteine ligase | 1.08 | 6.86 |
| *ZMO1194* | DNA protecting protein DprA | 1.32 | 6.92 |
| *ZMO1201* | glycine hydroxymethyltransferase | 1.26 | 7.79 |
| *ZMO1202* | transcriptional regulator NrdR | 2.40 | 10.70 |
| *ZMO1203* | TrmH family RNA methyltransferase | 1.30 | 7.61 |
| *ZMO1209* | potassium transporter Kup | 1.62 | 6.92 |
| *ZMO1225* | ribosomal protein S6 | 1.78 | 10.14 |
| *ZMO1226* | ribosomal protein S18 | 2.26 | 8.72 |
| *ZMO1227* | ribosomal protein L9 | 2.15 | 8.91 |
| *ZMO1242* | peptidase M14 carboxypeptidase A | 1.34 | 8.28 |
| *ZMO1243* | DUF4136 domain-containing protein | 1.82 | 6.65 |
| *ZMO1246* | 50S ribosomal protein L36 | 2.40 | 8.83 |
| *ZMO1249* | hypothetical protein | 1.05 | 4.38 |
| *ZMO1253* | cytochrome c-type biogenesis protein NrfF/CcmH | 1.83 | 8.16 |
| *ZMO1254* | thiol:disulfide oxidoreductase CcmG | 2.12 | 9.58 |
| *ZMO1255* | cytochrome c-type biogenesis protein CcmF/NfrE | 1.68 | 8.39 |
| *ZMO1257* | hypothetical protein | 1.38 | 5.27 |
| *ZMO1261* | ABC transporter substrate-binding protein aliphatic sulfonates family SsuA | 3.21 | 7.28 |
| *ZMO1262* | alkanesulfonate transporter permease subunit SsuC | 1.59 | 3.37 |
| *ZMO1267* | GMP synthase large subunit | 1.92 | 8.73 |
| *ZMO1270* | 8-amino-7-oxononanoate synthase | 1.18 | 10.73 |
| *ZMO1277* | aminodeoxychorismate lyase | 2.14 | 10.88 |
| *ZMO1279* | acyl carrier protein | 1.71 | 8.21 |
| *ZMO1285* | solbitol dehydrogenase large subunit | 1.65 | 10.28 |
| *ZMO1294* | N-acetylmuramic acid 6-phosphate etherase MurQ | 1.38 | 8.44 |
| *ZMO1296* | uncharacterized protein | 1.10 | 7.41 |
| *ZMO1299* | putative capsular polysaccharide biosynthesis protein BcbG | 1.96 | 10.13 |
| *ZMO1302* | lipoate-protein ligase B | 1.57 | 7.83 |
| *ZMO1307* | iron-dependent fumarate hydratase | 1.14 | 10.26 |
| *ZMO1316* | polysaccharide biosynthesis protein | 1.13 | 5.98 |
| *ZMO1320* | 16S rRNA C967 or C1407 C5-methylase RsmB/RsmF family | 3.66 | 12.38 |
| *ZMO1321* | inosine-5'-monophosphate dehydrogenase | 3.00 | 12.42 |
| *ZMO1324* | HPr kinase | 1.90 | 10.67 |
| *ZMO1329* | NAD(+) kinase | 1.36 | 6.79 |
| *ZMO1334* | polyisoprenoid-binding periplasmic YceI-like protein | 1.70 | 10.11 |
| *ZMO1345* | aminopeptidase N | 1.13 | 9.89 |
| *ZMO1346* | EamA/RhaT family transporter | 1.17 | 8.63 |
| *ZMO1347* | threonine aldolase | 1.43 | 10.69 |
| *ZMO1355* | ABC transporter related protein | 1.12 | 6.44 |
| *ZMO1360* | pyruvate decarboxylase | 1.45 | 10.54 |
| *ZMO1369* | agmatine deiminase | 1.07 | 6.53 |
| *ZMO1384* | GTP-binding protein Era | 1.46 | 8.74 |
| *ZMO1389* | Thiol:disulfide interchange protein DsbD | 1.72 | 7.38 |
| *ZMO1393* | LPS export ABC transporter permease LptF | 1.46 | 10.99 |
| *ZMO1394* | LPS export ABC transporter permease LptG | 1.41 | 10.71 |
| *ZMO1395* | N-formylglutamate amidohydrolase | 1.13 | 8.12 |
| *ZMO1403* | peptidase M23 | 3.46 | 13.71 |
| *ZMO1408* | S9 family peptidase | 1.71 | 9.61 |
| *ZMO1417* | putative DEAD/DEAH box helicase | 1.21 | 8.18 |
| *ZMO1420* | 5-(carboxyamino)imidazole ribonucleotide mutase PurE | 1.87 | 10.00 |
| *ZMO1423* | peptidase M16 domain protein | 1.25 | 7.79 |
| *ZMO1429* | NodT family RND efflux system outer membrane lipoprotein | 1.14 | 6.79 |
| *ZMO1430* | MFP subunit of RND efflux system transporter | 1.38 | 7.23 |
| *ZMO1431* | DUF1656 domain-containing protein | 2.41 | 9.33 |
| *ZMO1432* | membrane protein component of efflux system | 2.38 | 11.28 |
| *ZMO1433* | DNA polymerase III delta subunit | 1.94 | 10.23 |
| *ZMO1434* | uncharacterized protein | 2.36 | 11.10 |
| *ZMO1435* | leucyl-tRNA synthetase | 1.30 | 9.88 |
| *ZMO1441* | FAD dependent oxidoreductase | 1.93 | 11.64 |
| *ZMO1444* | glycine--tRNA ligase subunit beta | 2.10 | 11.54 |
| *ZMO1446* | glycine--tRNA ligase subunit alpha | 2.82 | 11.45 |
| *ZMO1451* | purine nucleoside permease | 1.40 | 8.66 |
| *ZMO1452* | MFS family predicted arabinose efflux permease araJ | 2.67 | 10.64 |
| *ZMO1457* | major facilitator superfamily transporter | 3.69 | 9.09 |
| *ZMO1464* | hypothetical protein | 1.34 | 5.06 |
| *ZMO1465* | putative amino acid permease | 1.54 | 6.43 |
| *ZMO1470* | transglycosylase domain-containing protein | 2.22 | 11.15 |
| *ZMO1471* | GTP-binding protein TypA/BipA | 1.17 | 7.09 |
| *ZMO1475* | TonB-dependent receptor plug domain | 2.93 | 7.58 |
| *ZMO1490* | putative outer membrane associated protein | 1.57 | 10.16 |
| *ZMO1494* | acetylglutamate kinase | 1.87 | 10.32 |
| *ZMO1495* | ATP/cobalamin adenosyltransferase | 1.09 | 6.11 |
| *ZMO1496* | phosphoenolpyruvate carboxylase | 1.55 | 9.27 |
| *ZMO1498* | diadenosine tetraphosphate hydrolase | 1.72 | 10.29 |
| *ZMO1500* | imidazole glycerol phosphate synthase cyclase subunit | 1.60 | 9.50 |
| *ZMO1501* | 1-(5-phosphoribosyl)-5-[(5- phosphoribosylamino)methylideneamino] imidazole-4-carboxamide isomerase | 2.26 | 11.80 |
| *ZMO1502* | imidazole glycerol phosphate synthase subunit HisH | 1.78 | 11.18 |
| *ZMO1503* | Imidazoleglycerol-phosphate dehydratase | 1.44 | 7.56 |
| *ZMO1504* | DUF1321 domain-containing protein | 1.40 | 9.75 |
| *ZMO1507* | inorganic diphosphatase | 2.25 | 10.23 |
| *ZMO1508* | histidine--tRNA ligase | 1.45 | 8.83 |
| *ZMO1509* | peptide chain release factor 1 | 1.47 | 10.19 |
| *ZMO1510* | protein-(glutamine-N5) methyltransferase release factor-specific | 2.22 | 11.66 |
| *ZMO1513* | phenylalanine--tRNA ligase subunit beta | 1.30 | 9.91 |
| *ZMO1514* | phenylalanine--tRNA ligase subunit alpha | 1.52 | 9.89 |
| *ZMO1518* | inositol monophosphatase | 3.10 | 12.96 |
| *ZMO1519* | ribose-phosphate pyrophosphokinase | 2.63 | 14.35 |
| *ZMO1528* | multidrug efflux pump subunit AcrB | 1.21 | 7.50 |
| *ZMO1532* | phosphoribosylformylglycinamidine synthase subunit PurQ | 1.32 | 8.72 |
| *ZMO1552* | transcription antitermination factor NusB | 1.28 | 8.96 |
| *ZMO1553* | thiamine-monophosphate kinase | 1.74 | 11.14 |
| *ZMO1561* | short-chain dehydrogenase/reductase SDR | 1.57 | 9.06 |
| *ZMO1562* | RNA pyrophosphohydrolase | 1.54 | 11.01 |
| *ZMO1563* | DUF481 domain-containing protein | 1.26 | 7.59 |
| *ZMO1571* | cytochrome bd ubiquinol oxidase subunit I | 2.21 | 12.04 |
| *ZMO1572* | cytochrome d ubiquinol oxidase subunit II | 1.85 | 10.74 |
| *ZMO1599* | hopanoid biosynthesis associated RND transporter like protein HpnN | 2.02 | 10.79 |
| *ZMO1600* | homoserine kinase | 1.37 | 8.63 |
| *ZMO1601* | ribonuclease H | 2.60 | 8.63 |
| *ZMO1608* | enolase | 1.03 | 5.98 |
| *ZMO1625* | ribonuclease P protein component | 1.70 | 7.42 |
| *ZMO1626* | YidC translocase/secretase | 1.69 | 7.26 |
| *ZMO1633* | uncharacterized protein | 1.28 | 8.24 |
| *ZMO1640* | tryptophanyl-tRNA synthetase | 1.07 | 8.48 |
| *ZMO1649* | gluconolactonase | 2.73 | 12.92 |
| *ZMO1651* | signal transduction protein | 1.78 | 10.13 |
| *ZMO1652* | 2-nitropropane dioxygenase NPD | 1.27 | 7.25 |
| *ZMO1653* | aspartate kinase | 1.16 | 8.38 |
| *ZMO1664* | rRNA large subunit methyltransferase H RlmH | 1.19 | 6.78 |
| *ZMO1673* | aldo/keto reductase | 1.43 | 9.97 |
| *ZMO1676* | DUF721 domain-containing protein | 1.04 | 7.19 |
| *ZMO1681* | aspartate-alanine antiporter | 1.47 | 6.88 |
| *ZMO1682* | aspartate/methionine/tyrosine aminotransferase | 1.32 | 7.69 |
| *ZMO1683* | type 2 L-asparaginase | 1.32 | 8.15 |
| *ZMO1684* | phosphoserine aminotransferase | 1.04 | 9.67 |
| *ZMO1686* | ATP phosphoribosyltransferase regulatory subunit HisZ | 2.71 | 12.37 |
| *ZMO1687* | adenylosuccinate synthase | 1.69 | 10.24 |
| *ZMO1707* | orotate phosphoribosyltransferase | 2.53 | 11.09 |
| *ZMO1708* | pyridoxal phosphate biosynthetic protein PdxJ | 1.56 | 9.21 |
| *ZMO1709* | Holo-[acyl-carrier-protein] synthase | 2.71 | 13.29 |
| *ZMO1712* | peptidylprolyl isomerase FKBP-type | 1.03 | 7.31 |
| *ZMO1715* | biopolymer transport protein ExbD/TolR | 1.26 | 8.85 |
| *ZMO1717* | TonB family protein | 1.07 | 7.56 |
| *ZMO1718* | uncharacterized protein | 1.67 | 10.91 |
| *ZMO1719* | fructokinase | 2.84 | 13.09 |
| *ZMO1728* | uncharacterized protein | 2.02 | 11.96 |
| *ZMO1740* | DUF3297 domain-containing protein | 1.89 | 12.53 |
| *ZMO1745* | methionine synthase MetH2 | 1.74 | 10.97 |
| *ZMO1746* | 5-methyltetrahydrofolate--homocysteine methyltransferase MetH1 | 2.20 | 11.58 |
| *ZMO1747* | 5,10-methylenetetrahydrofolate reductase | 2.07 | 9.81 |
| *ZMO1748* | ArsR family transcriptional regulator | 2.90 | 9.40 |
| *ZMO1749* | lipoprotein MlaA | 2.65 | 11.40 |
| *ZMO1755* | thymidylate synthase | 1.52 | 8.93 |
| *ZMO1756* | gluconate transporter GntP | 3.07 | 11.45 |
| *ZMO1757* | gluconate kinase GntK | 1.20 | 9.49 |
| *ZMO1762* | UPF0102 domain-containing protein | 1.39 | 10.43 |
| *ZMO1764* | murein L,D-transpeptidase YcbB/YkuD-like protein | 1.62 | 11.00 |
| *ZMO1768* | diaminopimelate decarboxylase | 1.44 | 11.08 |
| *ZMO1769* | uncharacterized protein | 1.76 | 9.38 |
| *ZMO1781* | mechanosensitive ion channel MscS | 1.46 | 7.06 |
| *ZMO1782* | hypothetical protein | 1.05 | 2.65 |
| *ZMO1784* | hypothetical protein | 1.18 | 6.38 |
| *ZMO1785* | mechanosensitive ion channel MscS | 1.51 | 7.54 |
| *ZMO1792* | dihydroxy-acid dehydratase | 1.50 | 8.44 |
| *ZMO1793* | LysR family transcriptional regulator | 1.01 | 5.60 |
| *ZMO1794* | uncharacterized protein | 1.95 | 8.98 |
| *ZMO1796* | 3-phosphoshikimate 1-carboxyvinyltransferase | 2.34 | 13.10 |
| *ZMO1800* | PhzF family phenazine biosynthesis protein | 1.09 | 5.04 |
| *ZMO1802* | hypothetical protein | 4.48 | 14.03 |
| *ZMO1805* | HAD superfamily phosphoglycolate phosphatase | 1.28 | 10.29 |
| *ZMO1810* | electron transport complex RnfABCDGE type G subunit | 1.05 | 6.76 |
| *ZMO1845* | PhnA protein | 2.47 | 10.74 |
| *ZMO1853* | dihydrodipicolinate synthetase | 1.17 | 9.05 |
| *ZMO1856* | major facilitator superfamily transporter | 1.18 | 6.91 |
| *ZMO1859* | carbohydrate porin | 1.28 | 7.98 |
| *ZMO1890* | mitochondrial processing peptidase-like protein | 1.14 | 8.45 |
| *ZMO1899* | 3-oxoacyl-(acyl-carrier-protein) synthase III | 1.11 | 8.71 |
| *ZMO1900* | fatty acid/phospholipid synthesis protein PlsX | 1.87 | 9.77 |
| *ZMO1910* | 50S ribosomal protein L25 | 1.58 | 8.86 |
| *ZMO1911* | peptidyl-tRNA hydrolase | 1.43 | 11.36 |
| *ZMO1924* | peptidase U32 | 1.07 | 9.00 |
| *ZMO1925* | sterol-binding peptidase U32 family peptidase | 1.36 | 10.02 |
| *ZMO1932* | uncharacterized protein | 1.93 | 8.86 |
| *ZMO1933* | uncharacterized protein | 1.95 | 9.90 |
| *ZMO1955* | malate dehydrogenase | 1.32 | 10.22 |
| *ZMO1956* | DNA repair protein RecN | 1.20 | 9.08 |
| *ZMO1957* | glycosyl transferase family 1 | 1.40 | 10.84 |
| *ZMO1959* | uncharacterized protein | 2.10 | 10.96 |
| *ZMO1962* | histidine kinase | 1.25 | 7.62 |
| *ZMO1963* | citrate synthase I | 1.99 | 10.82 |
| *ZMO1964* | glutamate--tRNA ligase | 2.09 | 12.74 |
| *ZMO1966* | uncharacterized protein | 1.53 | 5.99 |
| *ZMO1976* | uncharacterized protein | 1.72 | 8.47 |
| *ZMO1978* | partition protein ParB | 1.56 | 8.82 |
| *ZMO1980* | 16S rRNA methyltransferase GidB | 1.67 | 9.55 |
| *ZMO1981* | glucose inhibited division protein A GidA | 1.39 | 9.81 |
| *ZMO1996* | transcription termination factor Rho | 1.57 | 6.85 |
| *ZMO2003* | ribosomal protein S10 | 1.62 | 8.96 |
| *ZMO2012* | acyl carrier protein | 1.16 | 7.22 |
| *ZMO2013* | hypothetical protein | 1.84 | 8.50 |
| *ZMO2017* | capsule biosynthesis phosphatase | 1.57 | 6.63 |
| *ZMO2020* | hypothetical protein | 3.29 | 9.74 |
| *ZMO2024* | hypothetical protein | 2.04 | 8.69 |
| *ZMO2027* | hypothetical protein | 4.36 | 12.52 |
| *ZMO2031* | ribosomal protein L32 | 1.94 | 10.89 |
| *ZMO2045* | pseudo gene | 1.39 | 4.00 |
| *ZMO2046* | hypothetical protein | 1.35 | 4.56 |
| *ZMO2048* | hypothetical protein | 1.64 | 5.02 |
| *ZMO2049* | hypothetical protein | 1.43 | 7.60 |
| *ZMO2055* | hypothetical protein | 1.99 | 6.91 |
| *ZMO2063* | pseudo gene | 1.07 | 3.78 |
| *ZMO2066* | hypothetical protein | 2.83 | 10.87 |
| *ZMO2067* | hypothetical protein | 4.34 | 13.58 |
| *ZMO2071* | hypothetical protein | 2.59 | 6.56 |
| *ZMOp32x028* | Type I restriction endonuclease HsdS | 1.90 | 9.16 |
| *ZMOp32x029* | pseudo gene | 1.45 | 6.45 |
| *ZMOp32x034* | SAM-dependent methyltransferase | 1.21 | 8.08 |
| *ZMOp36x014* | Burkholderia phage phiE202 Gp27 family protein | 1.01 | 2.77 |
| *ZMOp36x043* | capsid scaffolding protein GpO | 2.19 | 7.20 |
| *ZMOp36x044* | bacteriophage P2 GpN major capsid | 2.13 | 6.48 |
| *ZMOp39x001* | hypothetical protein | 1.16 | 4.80 |
| *ZMOp39x034* | XRE family transcriptional regulator | 1.37 | 6.28 |
| **Downregulated gene in ZM4 cultured at 40℃ compared with 45℃** | | | |
| *ZMO0002* | histidinol-phosphate aminotransferase HisC | 2.42 | 7.28 |
| *ZMO0010* | DUF45 domain-containing protein | 1.18 | 7.05 |
| *ZMO0015* | heat-inducible transcription repressor HrcA | 5.75 | 14.14 |
| *ZMO0016* | GrpE protein | 4.93 | 13.28 |
| *ZMO0017* | RsmB-like methyltransferase | 3.63 | 12.69 |
| *ZMO0018* | ribulose-phosphate 3-epimerase | -1.01 | 7.45 |
| *ZMO0036* | DUF1491 domain-containing protein | -2.28 | 9.58 |
| *ZMO0037* | PTS IIA-like nitrogen-regulatory protein PtsN/NagE | -3.27 | 11.43 |
| *ZMO0038* | ribosomal protein S30Ae/sigma 54 modulation protein | -3.97 | 11.17 |
| *ZMO0050* | LysR family transcriptional regulator | -2.20 | 7.65 |
| *ZMO0052* | cyanate permease CynX | -2.00 | 8.63 |
| *ZMO0053* | alpha/beta hydrolase fold protein | -1.94 | 7.84 |
| *ZMO0057* | bacterial PH domain-containing protein | -2.23 | 9.82 |
| *ZMO0062* | aldo/keto reductase | -1.28 | 9.33 |
| *ZMO0089* | hypothetical protein | -1.28 | 6.86 |
| *ZMO0091* | hypothetical protein | -1.54 | 2.08 |
| *ZMO0095* | hypothetical protein | -1.65 | 4.52 |
| *ZMO0101* | NAD-dependent epimerase/dehydratase | -1.90 | 7.11 |
| *ZMO0103* | beta-lactamase | -1.46 | 7.89 |
| *ZMO0105* | 3-isopropylmalate dehydratase large subunit | -3.30 | 13.52 |
| *ZMO0106* | 3-isopropylmalate dehydratase small subunit | -2.92 | 12.95 |
| *ZMO0110* | glycosyl transferase family 2 | -1.54 | 8.97 |
| *ZMO0112* | uncharacterized protein | -4.43 | 12.35 |
| *ZMO0113* | aminodeoxychorismate synthase component 1 PabB | -1.52 | 6.48 |
| *ZMO0114* | glutamine amidotransferase of anthranilate synthase | -1.44 | 6.26 |
| *ZMO0116* | BadM/Rrf2 family transcriptional regulator | -1.82 | 5.33 |
| *ZMO0117* | hydroxylamine reductase | -2.11 | 7.26 |
| *ZMO0122* | uncharacterized protein | -3.73 | 11.34 |
| *ZMO0125* | YbaK/EbsC-like prolyl-tRNA editing protein | -2.89 | 12.03 |
| *ZMO0127* | S1/P1 nuclease | -1.18 | 7.08 |
| *ZMO0134* | Sel1 domain protein repeat-containing protein | -2.26 | 5.69 |
| *ZMO0137* | Sel1 domain protein repeat-containing protein | -1.07 | 7.76 |
| *ZMO0166* | peptidoglycan-associated lipoprotein | -1.64 | 10.29 |
| *ZMO0168* | pseudo gene | -1.05 | 7.21 |
| *ZMO0169* | short-chain dehydrogenase/reductase | -1.87 | 9.60 |
| *ZMO0171* | pseudo gene | -1.17 | 7.55 |
| *ZMO0173* | cell division protein ZapA | -2.37 | 10.51 |
| *ZMO0174* | putative SnoaL-like polynucleotide cyclase | -4.82 | 10.66 |
| *ZMO0179* | fructose-bisphosphate aldolase | -1.99 | 8.39 |
| *ZMO0180* | 1-hydroxy-2-methyl-2-(E)-butenyl 4-diphosphate synthase | -1.41 | 7.79 |
| *ZMO0199* | SOS-response transcriptional repressor LexA | -1.65 | 9.67 |
| *ZMO0203* | MerR family transcriptional regulator | -3.16 | 9.47 |
| *ZMO0208* | GCN5-related N-acetyltransferase | -3.49 | 11.79 |
| *ZMO0209* | ribosomal protein L27 | -1.90 | 8.75 |
| *ZMO0215* | 5-formyltetrahydrofolate cyclo-ligase | -4.40 | 12.36 |
| *ZMO0221* | CarD family transcriptional regulator | -3.14 | 10.72 |
| *ZMO0222* | DUF1330 domain-containing protein | -2.25 | 7.02 |
| *ZMO0223* | putative auto-transporter adhesin head GIN domain | -1.63 | 6.16 |
| *ZMO0224* | putative methionine biosynthesis methylase MetW | -2.05 | 9.52 |
| *ZMO0231* | uncharacterized protein | -3.71 | 12.50 |
| *ZMO0234* | protease Do | -2.25 | 9.73 |
| *ZMO0236* | Mrp family Chromosome partitioning ATPase | -1.97 | 8.06 |
| *ZMO0246* | ATP-dependent protease subunit HslV | -3.23 | 13.48 |
| *ZMO0247* | heat shock protein ATPase subunit HslU | -3.34 | 15.64 |
| *ZMO0252* | major intrinsic protein | -1.53 | 10.47 |
| *ZMO0253* | TolC family type I secretion outer membrane protein | -1.71 | 7.10 |
| *ZMO0257* | winged helix family two component transcriptional regulator | -2.78 | 12.48 |
| *ZMO0258* | histidine kinase | -1.18 | 8.68 |
| *ZMO0263* | uncharacterized protein | -1.35 | 9.88 |
| *ZMO0274* | RNA polymerase factor sigma-54 | -1.08 | 6.77 |
| *ZMO0281* | TetR family transcriptional repressor of RND efflux transport system | -2.65 | 10.97 |
| *ZMO0282* | MFP subunit of RND efflux system transporter | -3.69 | 13.39 |
| *ZMO0283* | hydrophobe/amphiphile efflux-1 (HAE1) family RND efflux system transporter | -2.00 | 10.59 |
| *ZMO0285* | NodT family RND efflux system outer membrane lipoprotein | -1.78 | 9.75 |
| *ZMO0286* | DUF541 domain-containing protein | -3.31 | 11.09 |
| *ZMO0287* | MFP subunit of RND efflux system transporter | -1.68 | 11.10 |
| *ZMO0290* | DUF445 domain-containing protein | -3.15 | 13.54 |
| *ZMO0293* | sugar transporter | -4.13 | 13.33 |
| *ZMO0294* | ribosomal protein L28 | -2.46 | 11.08 |
| *ZMO0298* | uncharacterized protein | -2.74 | 9.85 |
| *ZMO0318* | short-chain dehydrogenase/reductase SDR | -1.99 | 9.49 |
| *ZMO0319* | WGR domain protein | -1.63 | 8.03 |
| *ZMO0325* | uncharacterized protein | -1.74 | 11.40 |
| *ZMO0347* | RNA chaperone Hfq | -4.53 | 14.51 |
| *ZMO0348* | GTP-binding protein HflX | -2.26 | 11.84 |
| *ZMO0349* | DUF179 domain-containing protein | -1.07 | 6.99 |
| *ZMO0353* | 4-diphosphocytidyl-2C-methyl-D-erythritol synthase | -1.67 | 8.79 |
| *ZMO0354* | DNA mismatch repair protein MutL | -1.42 | 9.63 |
| *ZMO0355* | MreB/Mrl family cell shape determining protein | -2.40 | 13.61 |
| *ZMO0356* | rod shape-determining protein MreC | -1.69 | 9.99 |
| *ZMO0359* | peptidoglycan D,D-transpeptidase MrdA | -2.36 | 10.03 |
| *ZMO0362* | excinuclease ABC B subunit | -2.83 | 10.95 |
| *ZMO0368* | 6-phosphogluconate dehydratase | -1.43 | 8.85 |
| *ZMO0374* | levansucrase | -2.40 | 7.17 |
| *ZMO0376* | endopeptidase La | -3.70 | 12.33 |
| *ZMO0379* | PBSX family phage terminase large subunit | -1.08 | 3.74 |
| *ZMO0380* | hypothetical protein | -1.36 | 6.29 |
| *ZMO0381* | hypothetical protein | -1.58 | 6.55 |
| *ZMO0383* | hypothetical protein | -2.99 | 8.52 |
| *ZMO0384* | hypothetical protein | -3.08 | 6.50 |
| *ZMO0385* | hypothetical protein | -3.99 | 9.65 |
| *ZMO0387* | uncharacterized protein | -2.92 | 8.18 |
| *ZMO0388* | hypothetical protein | -2.16 | 6.35 |
| *ZMO0389* | hypothetical protein | -1.58 | 5.46 |
| *ZMO0390* | hypothetical protein | -1.25 | 4.68 |
| *ZMO0391* | hypothetical protein | -1.95 | 5.22 |
| *ZMO0392* | hypothetical protein | -2.71 | 9.04 |
| *ZMO0393* | hypothetical protein | -1.09 | 3.71 |
| *ZMO0395* | hypothetical protein | -1.31 | 3.43 |
| *ZMO0397* | hypothetical protein | -1.41 | 3.74 |
| *ZMO0398* | hypothetical protein | -1.98 | 6.55 |
| *ZMO0399* | peptidase S74 domain-containing protein | -2.21 | 7.25 |
| *ZMO0400* | hypothetical protein | -2.26 | 9.12 |
| *ZMO0405* | ATP-dependent Clp protease ATP-binding subunit clpA | -6.85 | 14.44 |
| *ZMO0406* | ABC-2 type transporter | -2.04 | 11.76 |
| *ZMO0407* | GcrA cell cycle regulator | -2.30 | 12.17 |
| *ZMO0422* | BadM/Rrf2 family transcriptional regulator | -2.47 | 11.24 |
| *ZMO0423* | FeS assembly protein SufB | -1.88 | 11.57 |
| *ZMO0425* | FeS assembly ATPase SufC | -1.08 | 7.74 |
| *ZMO0427* | SufS subfamily cysteine desulfurase | -1.68 | 11.13 |
| *ZMO0428* | FeS assembly SUF system protein | -4.16 | 11.61 |
| *ZMO0429* | iron-sulfur cluster assembly accessory protein | -1.82 | 10.07 |
| *ZMO0430* | pyrimidine 5'-nucleotidase | -1.63 | 10.92 |
| *ZMO0432* | ureohydrolase-like protein | -1.83 | 10.11 |
| *ZMO0435* | uncharacterized protein | -3.92 | 11.72 |
| *ZMO0436* | MAPEG (Membrane Associated Proteins in Eicosanoid and Glutathione metabolism) family protein | -1.13 | 5.38 |
| *ZMO0443* | ribonucleotide-diphosphate reductase subunit beta | -1.82 | 11.19 |
| *ZMO0469* | hypothetical protein | -4.93 | 13.30 |
| *ZMO0489* | hypothetical protein | -1.28 | 3.83 |
| *ZMO0490* | oligopeptidase B PtrB | -1.20 | 9.92 |
| *ZMO0510* | dihydrolipoyllysine-residue acetyltransferase | -1.22 | 8.31 |
| *ZMO0511* | thioesterase superfamily protein | -2.03 | 9.52 |
| *ZMO0552* | thymidine kinase | -1.10 | 8.28 |
| *ZMO0554* | translation initiation factor IF-2 | -1.37 | 9.40 |
| *ZMO0555* | DUF448 domain-containing protein | -1.54 | 11.06 |
| *ZMO0556* | transcription termination protein NusA | -2.68 | 12.15 |
| *ZMO0557* | DUF150/RimP N-terminal domain-containing protein | -3.50 | 12.99 |
| *ZMO0561* | TonB-dependent receptor plug domain | -2.46 | 9.95 |
| *ZMO0569* | succinate dehydrogenase (SQR) cytochrome b subunit | -1.79 | 11.17 |
| *ZMO0570* | ribosomal L11 methyltransferase | -1.49 | 10.02 |
| *ZMO0590* | uncharacterized protein | -1.01 | 6.14 |
| *ZMO0621* | flagellar biosynthesis anti-sigma factor FlgM | -1.73 | 8.51 |
| *ZMO0622* | uncharacterized protein | -1.51 | 6.63 |
| *ZMO0629* | flagellin domain-containing protein | -1.41 | 6.77 |
| *ZMO0653* | AI-2E family transporter | -1.12 | 7.26 |
| *ZMO0660* | chaperone protein DnaK | -4.57 | 11.99 |
| *ZMO0661* | chaperone protein DnaJ | -3.38 | 13.21 |
| *ZMO0665* | YnbE family lipoprotein | -2.69 | 8.61 |
| *ZMO0666* | uncharacterized protein | -2.19 | 11.90 |
| *ZMO0672* | excinuclease ABC C subunit | -1.60 | 6.65 |
| *ZMO0693* | OsmC family protein | -2.70 | 11.25 |
| *ZMO0694* | uncharacterized protein | -1.97 | 6.10 |
| *ZMO0699* | hypothetical protein | -2.55 | 9.49 |
| *ZMO0721* | SsrA-binding protein | -1.42 | 10.91 |
| *ZMO0724* | transcription termination/antitermination factor NusG | -2.21 | 7.73 |
| *ZMO0731* | DNA-directed RNA polymerase beta subunit | -4.22 | 13.19 |
| *ZMO0732* | DNA-directed RNA polymerase beta' subunit | -2.15 | 10.88 |
| *ZMO0733* | GCN5-related N-acetyltransferase | -1.63 | 5.05 |
| *ZMO0740* | general stress protein CsbD | -4.63 | 10.11 |
| *ZMO0749* | RNA polymerase sigma 32 subunit RpoH | -5.55 | 15.40 |
| *ZMO0750* | 23S rRNA pseudouridine synthase D RluD | -1.83 | 7.77 |
| *ZMO0753* | glutaredoxin 3 | -2.41 | 7.17 |
| *ZMO0754* | sterol-binding domain protein | -2.31 | 7.93 |
| *ZMO0786* | uncharacterized protein | -1.05 | 6.18 |
| *ZMO0819* | UDP-glucose 6-dehydrogenase | -2.46 | 13.10 |
| *ZMO0885* | hypothetical protein | -3.96 | 9.45 |
| *ZMO0895* | hypothetical protein | -1.27 | 4.27 |
| *ZMO0900* | glutamyl-tRNA synthetase | -1.51 | 12.38 |
| *ZMO0901* | hypothetical protein | -1.13 | 8.68 |
| *ZMO0903* | 2-isopropylmalate synthase | -3.04 | 11.65 |
| *ZMO0910* | ATPase component of polysaccharide export system | -1.83 | 10.72 |
| *ZMO0915* | copper-translocating P-type ATPase | -3.97 | 12.57 |
| *ZMO0916* | heavy metal transport/detoxification protein | -5.20 | 13.31 |
| *ZMO0917* | 2-nitropropane dioxygenase NPD | -1.21 | 9.74 |
| *ZMO0920* | uncharacterized protein | -2.24 | 8.33 |
| *ZMO0921* | uncharacterized protein | -3.01 | 10.36 |
| *ZMO0922* | helix-turn-helix domain-containing protein | -1.68 | 7.39 |
| *ZMO0929* | hypothetical protein | -1.19 | 5.26 |
| *ZMO0930* | hypothetical protein | -1.75 | 6.72 |
| *ZMO0931* | hypothetical protein | -2.70 | 9.28 |
| *ZMO0932* | DUF847/glycoside hydrolase family 108 domain-containing protein | -3.33 | 7.22 |
| *ZMO0934* | secretion-related protein | -3.31 | 7.79 |
| *ZMO0935* | glutathione S-transferase domain protein | -1.30 | 7.63 |
| *ZMO0941* | NAD-dependent epimerase/dehydratase | -1.52 | 9.31 |
| *ZMO0948* | ATP-dependent Clp protease proteolytic subunit ClpP | -3.44 | 12.89 |
| *ZMO0949* | ATP-dependent Clp protease ATP-binding subunit ClpX | -3.52 | 15.84 |
| *ZMO0964* | NodT family RND efflux system outer membrane lipoprotein | -3.16 | 12.36 |
| *ZMO0965* | efflux pump membrane protein | -1.88 | 10.54 |
| *ZMO0976* | xylose reductase | -1.56 | 10.69 |
| *ZMO0988* | DUF465 domain-containing protein | -2.57 | 10.05 |
| *ZMO0989* | heat-shock protein IbpA | -11.39 | 16.89 |
| *ZMO0994* | uncharacterized protein | -2.73 | 9.26 |
| *ZMO0996* | mechanosensitive ion channel MscS | -1.98 | 8.96 |
| *ZMO1007* | UPF0187 domain-containing protein | -1.04 | 8.00 |
| *ZMO1011* | YacG/DUF329 family protein | -1.83 | 6.58 |
| *ZMO1021* | alpha/beta fold family hydrolase-like protein | -1.23 | 6.33 |
| *ZMO1034* | calcium-binding EF-hand-containing protein | -3.34 | 13.85 |
| *ZMO1041* | NUDIX-like hydrolase | -2.19 | 11.78 |
| *ZMO1043* | LemA family protein | -1.23 | 9.43 |
| *ZMO1045* | phosphate-selective porin O and P | -2.05 | 9.87 |
| *ZMO1060* | superoxide dismutase | -2.78 | 12.07 |
| *ZMO1061* | Fis family sigma54-specific transcriptional acivator | -3.55 | 12.42 |
| *ZMO1062* | putative peripheral inner membrane phage shock protein pspD | -4.15 | 11.01 |
| *ZMO1063* | phage shock protein A PspA | -3.98 | 12.57 |
| *ZMO1064* | phage shock protein B PspB | -2.99 | 10.56 |
| *ZMO1065* | phage shock protein C PspC | -2.48 | 9.10 |
| *ZMO1069* | heat shock protein DnaJ domain protein | -1.06 | 8.89 |
| *ZMO1095* | nucleoside triphosphate pyrophosphohydrolase MazG | -2.76 | 8.58 |
| *ZMO1097* | thioredoxin-domain containing protein | -3.13 | 10.59 |
| *ZMO1113* | FAD-dependent pyridine nucleotide-disulfide oxidoreductase | -2.54 | 10.61 |
| *ZMO1129* | uncharacterized protein | -1.18 | 8.83 |
| *ZMO1130* | CinA domain-containing protein | -1.11 | 7.78 |
| *ZMO1133* | carbonate dehydratase | -2.40 | 10.75 |
| *ZMO1136* | MauG-like cytochrome-c peroxidase | -1.25 | 9.05 |
| *ZMO1139* | acetolactate synthase large subunit | -1.87 | 8.76 |
| *ZMO1140* | acetolactate synthase small subunit | -1.91 | 9.73 |
| *ZMO1156* | ribosomal protein S2 | -1.56 | 11.58 |
| *ZMO1158* | aspartate racemase | -2.97 | 10.48 |
| *ZMO1166* | DNA recombination/repair protein RecA | -3.79 | 12.11 |
| *ZMO1169* | BadF/BadG/BcrA/BcrD type ATPase | -1.06 | 8.44 |
| *ZMO1170* | antibiotic biosynthesis monooxygenase | -1.87 | 11.00 |
| *ZMO1177* | winged helix family two component transcriptional regulator | -1.39 | 11.62 |
| *ZMO1178* | phosphoribosyl-AMP cyclohydrolase | -2.20 | 11.17 |
| *ZMO1187* | formamidopyrimidine-DNA glycosylase | -2.82 | 9.28 |
| *ZMO1205* | hypothetical protein | -2.38 | 10.20 |
| *ZMO1213* | uncharacterized protein | -2.72 | 10.57 |
| *ZMO1216* | signal transduction histidine kinase | -1.14 | 6.97 |
| *ZMO1219* | twin-arginine translocase subunit TatB | -1.21 | 8.78 |
| *ZMO1220* | twin-arginine translocase subunit TatA | -1.22 | 6.27 |
| *ZMO1221* | uncharacterized protein | -1.59 | 9.00 |
| *ZMO1232* | glycosyl transferase family 1 | -1.48 | 7.90 |
| *ZMO1234* | deoxyxylulose-5-phosphate synthase | -1.10 | 8.38 |
| *ZMO1235* | Fur family ferric uptake regulator | -2.68 | 10.93 |
| *ZMO1236* | Zn-dependent alcohol dehydrogenase | -3.00 | 11.32 |
| *ZMO1237* | lactate dehydrogenase | -3.88 | 11.01 |
| *ZMO1271* | siroheme synthase CysG | -3.21 | 9.87 |
| *ZMO1289* | putative transglycosylase-associated protein | -1.37 | 6.89 |
| *ZMO1298* | TonB-dependent receptor plug domain | -1.39 | 8.76 |
| *ZMO1303* | pyrroline-5-carboxylate reductase | -1.62 | 9.31 |
| *ZMO1304* | DUF1790 domain-containing protein | -1.20 | 8.95 |
| *ZMO1305* | DUF526 domain-containing protein | -1.30 | 4.41 |
| *ZMO1312* | PpiC-type peptidyl-prolyl cis-trans isomerase | -1.47 | 8.06 |
| *ZMO1335* | flavoprotein WrbA | -2.29 | 10.76 |
| *ZMO1337* | hydroquinone detoxification protein | -1.33 | 7.46 |
| *ZMO1341* | Zn-dependent metalloprotease | -3.00 | 10.84 |
| *ZMO1352* | intergral membrane protein | -3.74 | 12.25 |
| *ZMO1366* | 30S ribosomal protein S4 | -1.23 | 9.33 |
| *ZMO1372* | short-chain dehydrogenase/reductase SDR | -2.07 | 10.71 |
| *ZMO1373* | uncharacterized protein | -3.66 | 10.60 |
| *ZMO1374* | NAD-dependent epimerase/dehydratase | -1.10 | 8.41 |
| *ZMO1375* | ribonuclease III | -1.97 | 10.90 |
| *ZMO1385* | toxic anion resistance family protein | -1.65 | 9.03 |
| *ZMO1386* | uncharacterized protein | -1.62 | 9.70 |
| *ZMO1387* | winged helix family two component transcriptional regulator | -2.24 | 11.13 |
| *ZMO1390* | uncharacterized protein | -1.64 | 7.69 |
| *ZMO1401* | exodeoxyribonuclease III XthA | -1.60 | 9.98 |
| *ZMO1402* | iron-sulfur cluster assembly accessory protein | -1.56 | 7.78 |
| *ZMO1406* | alpha/beta hydrolase fold protein | -1.59 | 10.13 |
| *ZMO1410* | bacterioferritin | -5.67 | 13.52 |
| *ZMO1414* | tRNA(adenosine(37)-N6)- threonylcarbamoyltransferase complex dimerization subunit type 1 TsaB | -1.29 | 9.08 |
| *ZMO1415* | Nfu/NifU-like scaffold protein | -2.25 | 10.88 |
| *ZMO1424* | ATP-dependent chaperone ClpB | -3.91 | 15.18 |
| *ZMO1425* | thiamine monophosphate synthase | -1.49 | 10.43 |
| *ZMO1426* | DNA repair protein RadC | -2.27 | 9.68 |
| *ZMO1428* | diacylglycerol kinase catalytic region | -1.54 | 8.96 |
| *ZMO1439* | carbon-nitrogen hydrolase | -1.54 | 10.08 |
| *ZMO1440* | uncharacterized protein | -1.96 | 9.89 |
| *ZMO1447* | conjugative transfer protein GumN | -1.18 | 7.87 |
| *ZMO1448* | hypothetical protein | -2.53 | 9.93 |
| *ZMO1449* | mannitol-1-phosphate dehydrogenase | -6.60 | 15.05 |
| *ZMO1459* | nicotinamide mononucleotide transporter PnuC | -1.42 | 4.62 |
| *ZMO1460* | 3-mercaptopyruvate sulfurtransferase | -1.35 | 7.98 |
| *ZMO1485* | deoxyguanosinetriphosphate triphosphohydrolase | -2.48 | 9.63 |
| *ZMO1489* | 3-deoxy-D-manno-octulosonate cytidylyltransferase | -1.16 | 8.90 |
| *ZMO1506* | uncharacterized protein | -1.27 | 8.32 |
| *ZMO1511* | uncharacterized protein | -1.31 | 9.78 |
| *ZMO1522* | TonB-dependent receptor | -1.43 | 5.33 |
| *ZMO1524* | EamA domain-containing protein | -1.43 | 10.09 |
| *ZMO1533* | hypothetical protein | -3.49 | 12.84 |
| *ZMO1535* | pseudo gene | -1.03 | 7.07 |
| *ZMO1540* | FeoA family protein | -2.02 | 11.70 |
| *ZMO1541* | ferrous iron transport protein B | -1.19 | 8.52 |
| *ZMO1543* | cobalt chelatase subunit CobT | -1.56 | 5.13 |
| *ZMO1544* | cobalt chelatase subunit CobS | -2.28 | 6.65 |
| *ZMO1545* | heat shock protein DnaJ domain protein | -3.42 | 7.84 |
| *ZMO1549* | BolA family protein | -1.80 | 8.56 |
| *ZMO1581* | antibiotic biosynthesis monooxygenase | -2.44 | 9.67 |
| *ZMO1582* | uracil-DNA glycosylase-like protein | -2.64 | 11.46 |
| *ZMO1584* | DNA replication and repair protein RecF | -1.10 | 5.74 |
| *ZMO1586* | bacterioferritin | -2.99 | 9.17 |
| *ZMO1587* | hypothetical protein | -1.86 | 9.21 |
| *ZMO1588* | excinuclease ABC A subunit | -6.48 | 19.08 |
| *ZMO1589* | toll/interleukin-1 receptor (TIR) domain-containing protein | -3.02 | 10.75 |
| *ZMO1596* | iron-containing alcohol dehydrogenase | -3.29 | 10.74 |
| *ZMO1602* | peptidase | -1.41 | 11.21 |
| *ZMO1609* | uncharacterized protein | -1.85 | 9.88 |
| *ZMO1612* | toluene tolerance family protein | -1.69 | 12.05 |
| *ZMO1615* | transcription elongation factor GreA | -1.87 | 10.78 |
| *ZMO1618* | carbamoyl-phosphate synthase small subunit | -1.58 | 8.21 |
| *ZMO1622* | DNA primase | -2.16 | 12.47 |
| *ZMO1623* | RNA polymerase sigma 70 subunit RpoD | -2.41 | 9.71 |
| *ZMO1631* | TonB-dependent siderophore receptor | -1.77 | 8.27 |
| *ZMO1632* | succinyl-diaminopimelate desuccinylase | -1.65 | 10.74 |
| *ZMO1635* | hypothetical protein | -1.57 | 9.66 |
| *ZMO1636* | import inner membrane translocase subunit Tim44 | -2.50 | 12.78 |
| *ZMO1641* | uncharacterized protein | -1.82 | 8.90 |
| *ZMO1659* | ATP-dependent metalloprotease FtsH | -3.57 | 14.04 |
| *ZMO1660* | uncharacterized protein | -1.88 | 12.38 |
| *ZMO1661* | gamma-glutamyl phosphate reductase | -1.18 | 8.64 |
| *ZMO1668* | disulfide bond formation protein DsbB | -1.73 | 10.81 |
| *ZMO1670* | uncharacterized protein | -1.00 | 9.77 |
| *ZMO1671* | uncharacterized protein | -2.37 | 10.75 |
| *ZMO1679* | YgdH family protein | -1.76 | 8.49 |
| *ZMO1690* | chaperone DnaJ domain protein | -5.54 | 11.52 |
| *ZMO1696* | putative NADPH:quinone reductase-related Zn-dependent oxidoreductase | -2.53 | 9.35 |
| *ZMO1697* | HxlR family transcriptional regulator | -5.54 | 8.77 |
| *ZMO1698* | GTP cyclohydrolase II | -2.38 | 9.41 |
| *ZMO1700* | uncharacterized protein | -1.63 | 6.80 |
| *ZMO1704* | ATP-dependent protease La (LON) substrate-binding domain | -2.37 | 9.23 |
| *ZMO1705* | thioredoxin domain-containing protein | -4.19 | 11.48 |
| *ZMO1720* | DNA-directed RNA polymerase omega subunit | -3.98 | 13.58 |
| *ZMO1721* | glyoxalase/bleomycin resistance protein dioxygenase | -5.74 | 14.27 |
| *ZMO1732* | alkyl hydroperoxide reductase | -2.37 | 8.66 |
| *ZMO1738* | LytTR family two component transcriptional regulator | -2.08 | 13.32 |
| *ZMO1743* | bis(5'nucleosyl)-tetraphosphatase ApaH | -1.32 | 8.44 |
| *ZMO1753* | ferredoxin--NADP(+) reductase Fpr | -1.83 | 8.73 |
| *ZMO1754* | succinate-semialdehyde dehydrogenase SSADH | -4.37 | 11.85 |
| *ZMO1767* | UTP-glucose-1-phosphate uridylyltransferase | -2.31 | 13.12 |
| *ZMO1776* | aminopeptidase N | -2.95 | 11.70 |
| *ZMO1812* | electron transport complex RnfABCDGE type C subunit | -1.37 | 7.94 |
| *ZMO1813* | electron transport complex RnfABCDGE type B subunit | -2.15 | 9.74 |
| *ZMO1814* | electron transport complex RnfABCDGE type A subunit | -2.66 | 10.05 |
| *ZMO1815* | TonB-dependent siderophore receptor | -1.24 | 5.97 |
| *ZMO1822* | TonB-dependent siderophore receptor | -3.70 | 10.52 |
| *ZMO1823* | nitrogenase reductase iron protein | -1.71 | 6.53 |
| *ZMO1824* | nitrogenase molybdenum-iron protein alpha chain | -1.31 | 4.92 |
| *ZMO1825* | nitrogenase molybdenum-iron protein beta chain | -1.08 | 5.66 |
| *ZMO1837* | modD protein | -2.62 | 7.94 |
| *ZMO1838* | TOBE domain protein | -4.33 | 9.80 |
| *ZMO1840* | isochorismatase hydrolase | -1.51 | 9.49 |
| *ZMO1842* | ApbE family lipoprotein | -1.34 | 7.01 |
| *ZMO1850* | hypothetical protein | -3.24 | 9.59 |
| *ZMO1851* | flavodoxin | -4.33 | 10.49 |
| *ZMO1861* | 2-nitropropane dioxygenase NPD | -3.68 | 12.11 |
| *ZMO1866* | beta-lactamase domain protein | -1.89 | 11.92 |
| *ZMO1867* | Baf family transcriptional acitvator | -2.30 | 12.72 |
| *ZMO1868* | biotin--[acetyl-CoA-carboxylase] ligase | -2.46 | 12.88 |
| *ZMO1871* | quinolinate synthetase complex A subunit | -1.09 | 8.24 |
| *ZMO1872* | uncharacterized protein | -3.24 | 13.49 |
| *ZMO1873* | glutaredoxin-like protein | -4.78 | 13.84 |
| *ZMO1874* | BolA family transcriptional regulator | -2.75 | 12.45 |
| *ZMO1875* | DUF1476 domain-containing protein | -2.70 | 12.13 |
| *ZMO1885* | NADH:flavin oxidoreductase | -1.08 | 7.54 |
| *ZMO1902* | uroporphyrinogen III synthase | -1.62 | 9.00 |
| *ZMO1903* | porphobilinogen deaminase | -2.96 | 9.78 |
| *ZMO1907* | DNA mismatch repair protein MutS | -1.11 | 7.54 |
| *ZMO1909* | DUF177 domain-containing protein | -1.73 | 8.53 |
| *ZMO1918* | adenosylmethionine--8-amino-7-oxononanoate aminotransferase BioA | -1.53 | 9.05 |
| *ZMO1919* | thiol-disulfide isomerase | -1.59 | 10.93 |
| *ZMO1920* | uncharacterized protein | -8.52 | 16.62 |
| *ZMO1921* | primosomal protein N' | -4.14 | 17.15 |
| *ZMO1928* | chaperonin Cpn10 | -8.71 | 14.96 |
| *ZMO1929* | chaperonin GroEL | -8.35 | 14.50 |
| *ZMO1930* | integrase family protein | -2.61 | 8.67 |
| *ZMO1931* | DUF1016 domain-containing protein | -3.68 | 9.82 |
| *ZMO1940* | hypothetical protein | -3.50 | 5.89 |
| *ZMO1941* | type IV secretory pathway protease TraF-like protein | -3.71 | 4.46 |
| *ZMO1943* | VirD2 and DUF3363 domain-containing protein | -4.41 | 11.51 |
| *ZMO1944* | GntR transcriptional regulator with aminotransferase domain | -6.07 | 13.27 |
| *ZMO1945* | PhzF-like isomerase | -2.47 | 7.23 |
| *ZMO1946* | short-chain dehydrogenase/reductase SDR | -1.17 | 4.49 |
| *ZMO1947* | RidA (reactive intermediate/imine deaminase A) family protein | -1.51 | 7.18 |
| *ZMO1985* | pseudo gene | -1.37 | 6.00 |
| *ZMO1992* | carboxymethylenebutenolidase | -1.24 | 9.18 |
| *ZMO1997* | CopD family protein | -1.01 | 7.84 |
| *ZMO2000* | AcrB/AcrD/AcrF family protein | -4.48 | 13.26 |
| *ZMO2002* | ribosomal protein S12 | -1.16 | 10.31 |
| *ZMO2006* | preprotein translocase SecE subunit | -1.83 | 7.21 |
| *ZMO2010* | pseudo gene | -1.79 | 7.30 |
| *ZMO2011* | entericidin A/B family protein | -4.01 | 10.74 |
| *ZMO2016* | hypothetical protein | -3.32 | 6.49 |
| *ZMO2019* | BFD domain protein (2Fe-2S)-binding domain protein | -6.44 | 11.38 |
| *ZMO2028* | nif-specific ferredoxin III | -1.28 | 3.92 |
| *ZMO2029* | hypothetical protein | -2.64 | 7.35 |
| *ZMO2033* | XRE family transcriptional regulator | -1.42 | 6.37 |
| *ZMO2034* | DUF2958 domain-containing protein | -1.63 | 4.90 |
| *ZMO2035* | RepA replication protein | -1.87 | 4.81 |
| *ZMO2036* | pseudo gene | -3.92 | 6.62 |
| *ZMO2037* | hypothetical protein | -3.19 | 7.04 |
| *ZMO2041* | Sel1 domain protein repeat-containing protein | -5.19 | 13.37 |
| *ZMO2042* | hypothetical protein | -2.40 | 4.40 |
| *ZMO2044* | redox-sensitive transcriptional activator SoxR | -2.11 | 7.92 |
| *ZMO2053* | uncharacterized protein | -3.67 | 12.97 |
| *ZMO2056* | uncharacterized protein | -4.39 | 12.05 |
| *ZMO2059* | RNase P RNA component class A | -1.81 | 7.62 |
| *ZMO2060* | hypothetical protein | -4.63 | 10.91 |
| *ZMO2062* | hypothetical protein | -1.68 | 6.21 |
| *ZMO2064* | hypothetical protein | -1.66 | 3.25 |
| *ZMO2070* | hypothetical protein | -1.41 | 3.63 |
| *ZMO2074* | hypothetical protein | -1.26 | 2.68 |
| *ZMO2076* | hypothetical protein | -1.50 | 4.62 |
| *ZMO2077* | hypothetical protein | -1.35 | 5.73 |
| *ZMOp32x002* | RelB or DinJ-like antitoxin | -2.36 | 12.41 |
| *ZMOp32x003* | pseudo gene | -2.49 | 10.51 |
| *ZMOp32x004* | P-loop containing nucleoside triphosphate hydrolase domain protein | -2.73 | 11.72 |
| *ZMOp32x005* | uncharacterized protein | -1.64 | 8.93 |
| *ZMOp32x006* | TonB-dependent receptor plug domain protein | -1.27 | 9.88 |
| *ZMOp32x007* | Ca2+-dependent phosphoinositide phospholipase C | -1.32 | 8.76 |
| *ZMOp32x008* | PIN domain protein | -4.25 | 13.12 |
| *ZMOp32x009* | SinI-like DNA-binding domain protein | -5.04 | 13.09 |
| *ZMOp32x010* | Macro or A1pp domain protein | -1.62 | 9.41 |
| *ZMOp32x011* | uncharacterized protein | -2.22 | 8.29 |
| *ZMOp32x012* | hypothetical protein | -2.31 | 7.38 |
| *ZMOp32x013* | hypothetical protein | -1.92 | 8.32 |
| *ZMOp32x014* | hypothetical protein | -1.93 | 8.55 |
| *ZMOp32x015* | Azospirillum phage Cd Gp10 family protein | -1.92 | 6.26 |
| *ZMOp32x019* | hypothetical protein | -1.63 | 4.63 |
| *ZMOp32x020* | MazE antitoxin | -3.45 | 12.23 |
| *ZMOp32x021* | MazF toxin | -3.02 | 13.99 |
| *ZMOp32x022* | SIR2-like protein | -2.56 | 11.75 |
| *ZMOp32x023* | MTH538 TIR-like domain | -1.19 | 4.36 |
| *ZMOp32x024* | phage integrase | -1.39 | 6.70 |
| *ZMOp32x030* | hypothetical protein | -2.40 | 10.90 |
| *ZMOp32x031* | hypothetical protein | -2.16 | 8.04 |
| *ZMOp32x033* | hypothetical protein | -1.57 | 7.59 |
| *ZMOp33x001* | hypothetical protein | -1.51 | 8.43 |
| *ZMOp33x002* | putative Zeta toxin-like kinase | -1.18 | 9.06 |
| *ZMOp33x005* | Vibrio phage ICP1 Orf50 family protein | -1.53 | 7.58 |
| *ZMOp33x006* | acyl-CoA N-acyltransferase | -1.44 | 7.27 |
| *ZMOp33x012* | 2-aminoethylphosphonate--pyruvate transaminase | -1.55 | 9.33 |
| *ZMOp33x013* | DUF3225 family protein with NTF2-like domain | -2.37 | 10.87 |
| *ZMOp33x014* | protein of unknown function DUF4089 | -2.14 | 10.29 |
| *ZMOp33x015* | AtzE type amidohydrolase | -1.13 | 7.61 |
| *ZMOp33x016* | short-chain dehydrogenase/reductase SDR | -1.39 | 7.89 |
| *ZMOp33x018* | bacterial bifunctional deaminase-reductase C-terminal domain-containing protein | -1.04 | 7.87 |
| *ZMOp33x019* | LysR family transcription regulator | -1.39 | 8.51 |
| *ZMOp33x022* | NADP-dependent oxidoreductase domain protein | -1.37 | 8.09 |
| *ZMOp33x023* | NADP-dependent oxidoreductase domain protein | -1.05 | 6.88 |
| *ZMOp33x024* | pseudo gene | -3.42 | 12.82 |
| *ZMOp33x025* | pseudo gene | -1.81 | 9.96 |
| *ZMOp33x026* | toxin RelE-like domain protein | -1.71 | 7.72 |
| *ZMOp33x027* | relaxosome protein TraY | -2.36 | 7.42 |
| *ZMOp33x028* | winged helix-turn-helix DNA-binding domain protein | -1.59 | 6.25 |
| *ZMOp33x029* | Azospirillum phage Cd Gp10 family protein | -4.24 | 9.90 |
| *ZMOp33x030* | hypothetical protein | -4.10 | 11.19 |
| *ZMOp33x031* | uncharacterized protein | -4.15 | 11.26 |
| *ZMOp33x032* | hypothetical protein | -4.87 | 9.31 |
| *ZMOp33x033* | hypothetical protein | -2.34 | 7.72 |
| *ZMOp33x036* | ribonuclease toxin BrnT of type II toxin-antitoxin system | -4.73 | 11.76 |
| *ZMOp33x037* | cro/C1-type transcriptional repressor | -4.80 | 14.69 |
| *ZMOp33x038* | Lambda repressor-like transcriptional regulator | -4.33 | 9.94 |
| *ZMOp33x039* | hypothetical protein | -2.99 | 10.71 |
| *ZMOp33x040* | death on curing (Doc) protein | -3.06 | 12.07 |
| *ZMOp36x016* | phage P2 GpU family protein | -1.20 | 3.46 |
| *ZMOp36x020* | hypothetical protein | -1.11 | 6.04 |
| *ZMOp36x022* | uncharacterized protein | -3.09 | 9.54 |
| *ZMOp36x023* | YafQ-like toxin | -3.44 | 11.55 |
| *ZMOp36x024* | antitoxin DinJ | -3.75 | 10.78 |
| *ZMOp36x028* | uncharacterized protein | -1.96 | 8.19 |
| *ZMOp36x029* | Lambda repressor-like DNA-binding domain protein | -2.50 | 10.69 |
| *ZMOp36x030* | toxin HigB-like | -2.85 | 9.83 |
| *ZMOp36x032* | uncharacterized protein | -1.06 | 2.74 |
| *ZMOp36x033* | zinc finger Ogr/Delta-type transcriptional activator | -1.70 | 6.19 |
| *ZMOp36x034* | Azospirillum phage Cd Gp10 family protein | -1.66 | 4.17 |
| *ZMOp36x035* | Azospirillum phage Cd Gp10 family protein | -2.03 | 6.80 |
| *ZMOp36x036* | winged helix-turn-helix DNA-binding domain protein | -1.09 | 4.97 |
| *ZMOp36x039* | CobQ/CobB/MinD/ParA nucleotide binding domain protein | -1.41 | 8.66 |
| *ZMOp36x040* | Arc-type ribbon-helix-helix | -1.80 | 7.57 |
| *ZMOp36x041* | PBSX family phage portal protein | -1.98 | 10.51 |
| *ZMOp36x049* | uncharacterized protein | -1.26 | 5.37 |
| *ZMOp36x051* | uncharacterized protein | -1.52 | 6.73 |
| *ZMOp36x052* | hypothetical protein | -2.21 | 10.19 |
| *ZMOp39x004* | uncharacterized protein | -1.44 | 7.01 |
| *ZMOp39x005* | RelB antitoxin/Antitoxin DinJ family toxin | -2.58 | 10.32 |
| *ZMOp39x006* | YafQ-like toxin | -1.97 | 7.65 |
| *ZMOp39x007* | TonB-dependent receptor beta-barrel protein | -1.97 | 10.63 |
| *ZMOp39x009* | putative partitioning protein ParA ATPase | -2.78 | 11.98 |
| *ZMOp39x010* | putative partitioning protein ParB | -2.36 | 9.45 |
| *ZMOp39x011* | AraC-type helix-turn-helix transcriptional regulator | -1.36 | 6.81 |
| *ZMOp39x013* | type I ABC transporter ATPase | -1.06 | 4.45 |
| *ZMOp39x017* | hypothetical protein | -1.29 | 5.94 |
| *ZMOp39x019* | pseudo gene | -2.81 | 7.83 |
| *ZMOp39x020* | hypothetical protein | -1.23 | 7.03 |
| *ZMOp39x021* | death on curing (Doc) protein | -1.51 | 8.39 |
| *ZMOp39x022* | helix-turn-helix DUF1870 domain-containing protein | -1.49 | 8.13 |
| *ZMOp39x023* | P-loop containing nucleoside triphosphate hydrolase domain protein | -2.09 | 9.83 |
| *ZMOp39x024* | hypothetical protein | -1.04 | 4.08 |
| *ZMOp39x025* | ParG family protein | -1.47 | 7.23 |
| *ZMOp39x026* | pseudo gene | -1.89 | 9.07 |
| *ZMOp39x029* | pseudo gene | -2.22 | 7.67 |
| *ZMOp39x030* | uncharacterized protein | -1.89 | 8.67 |
| *ZMOp39x031* | Alpha/Beta hydrolase fold | -2.31 | 9.37 |
| *ZMOp39x032* | winged helix-turn-helix DNA-binding domain protein | -2.20 | 8.27 |
| *ZMOp39x033* | hypothetical protein | -2.69 | 7.42 |
| *ZMOp39x035* | pseudo gene | -2.79 | 9.10 |
| *ZMOp39x036* | hypothetical protein | -2.73 | 10.66 |
| *ZMOp39x037* | hypothetical protein | -3.05 | 9.69 |

**Table S2_f.** List of significantly differentially expressed genes between ZM4 cultured at 36℃ and 40℃. Ratio is the log_2_-based expression difference between ZM4 cultured at 36℃ and 40℃ (36/40). Gene name with red and blue color font indicates up-regulated and down-regulated at 36℃ compared with 40℃, respectively.

| **Name** | **Product** | **Ratio** | **-log_10_ (p-value)** |
| --- | --- | --- | --- |
| **Upregulated gene in ZM4 cultured at 36℃ compared with 40℃** | | | |
| *ZMO0374* | levansucrase | 3.24 | 8.64 |
| *ZMO0375* | levansucrase/invertase | 2.29 | 6.97 |
| *ZMO0379* | PBSX family phage terminase large subunit | 1.95 | 6.27 |
| *ZMO0380* | hypothetical protein | 1.46 | 6.64 |
| *ZMO0381* | hypothetical protein | 1.84 | 7.27 |
| *ZMO0383* | hypothetical protein | 2.54 | 7.71 |
| *ZMO0384* | hypothetical protein | 2.02 | 4.62 |
| *ZMO0387* | uncharacterized protein | 2.09 | 6.57 |
| *ZMO0388* | hypothetical protein | 2.14 | 6.30 |
| *ZMO0389* | hypothetical protein | 1.69 | 5.77 |
| *ZMO0390* | hypothetical protein | 1.62 | 5.81 |
| *ZMO0391* | hypothetical protein | 2.05 | 5.44 |
| *ZMO0392* | hypothetical protein | 1.69 | 6.72 |
| *ZMO0393* | hypothetical protein | 1.22 | 4.15 |
| *ZMO0395* | hypothetical protein | 2.94 | 6.86 |
| *ZMO0397* | hypothetical protein | 2.57 | 6.28 |
| *ZMO0398* | hypothetical protein | 1.98 | 6.55 |
| *ZMO0399* | peptidase S74 domain-containing protein | 1.50 | 5.46 |
| *ZMO0930* | hypothetical protein | 1.68 | 6.53 |
| *ZMO0931* | hypothetical protein | 2.47 | 8.82 |
| *ZMO0932* | DUF847/glycoside hydrolase family 108 domain-containing protein | 2.77 | 6.34 |
| *ZMO0934* | secretion-related protein | 1.98 | 5.36 |
| *ZMO1062* | putative peripheral inner membrane phage shock protein pspD | 1.01 | 4.27 |
| **Downregulated gene in ZM4 cultured at 36℃ compared with 40℃** | | | |
| *ZMO0001* | TonB-dependent receptor | -1.75 | 6.74 |
| *ZMO0003* | adenylyl-sulfate kinase | -1.46 | 2.60 |
| *ZMO0004* | sulfate adenylyltransferase large subunit | -2.98 | 4.79 |
| *ZMO0005* | sulfate adenylyltransferase small subunit | -3.84 | 7.80 |
| *ZMO0006* | siroheme synthase CysG | -4.23 | 12.18 |
| *ZMO0007* | phosphoadenosine phosphosulfate reductase | -1.05 | 2.12 |
| *ZMO0008* | sulfite reductase (NADPH) hemoprotein beta-component | -1.62 | 3.29 |
| *ZMO0009* | sulfite reductase (NADPH) flavoprotein alpha chain | -2.22 | 6.13 |
| *ZMO0055* | anion permerase | -5.14 | 10.60 |
| *ZMO0311* | pyrroline-5-carboxylate reductase | -1.00 | 5.77 |
| *ZMO0747* | uncharacterized protein | -1.18 | 6.79 |
| *ZMO0748* | cysteine synthase | -3.90 | 10.54 |
| *ZMO1261* | ABC transporter substrate-binding protein aliphatic sulfonates family SsuA | -2.73 | 6.50 |
| *ZMO1262* | alkanesulfonate transporter permease subunit SsuC | -2.02 | 4.27 |
| *ZMO1263* | aliphatic sulfonates transport ATP-binding subunit SsuB | -1.10 | 2.47 |
| *ZMO1271* | siroheme synthase CysG | -1.27 | 5.38 |
| *ZMO1456* | LysE family translocator | -1.33 | 5.14 |
| *ZMO1463* | TonB-dependent receptor | -1.52 | 4.75 |
| *ZMO1475* | TonB-dependent receptor plug domain | -3.44 | 8.38 |
| *ZMO1849* | uncharacterized protein | -2.97 | 7.28 |
| *ZMOp32x014* | hypothetical protein | -1.17 | 6.11 |
| *ZMOp36x001* | N-acetylmuramoyl-L-alanine amidase domain protein | -1.56 | 5.04 |
| *ZMOp36x002* | hypothetical protein | -1.13 | 2.27 |
| *ZMOp36x003* | hypothetical protein | -1.10 | 3.09 |
| *ZMOp36x006* | baseplate assembly protein J | -1.76 | 6.23 |
| *ZMOp36x007* | tail protein I | -1.88 | 5.86 |
| *ZMOp36x008* | bulb-type lectin domain protein | -1.17 | 5.18 |
| *ZMOp36x009* | uncharacterized protein | -1.13 | 4.42 |
| *ZMOp36x033* | zinc finger Ogr/Delta-type transcriptional activator | -1.20 | 4.66 |
| *ZMOp36x034* | Azospirillum phage Cd Gp10 family protein | -1.34 | 3.35 |
| *ZMOp36x035* | Azospirillum phage Cd Gp10 family protein | -1.19 | 4.43 |
| *ZMOp36x043* | capsid scaffolding protein GpO | -1.84 | 6.38 |
| *ZMOp36x044* | bacteriophage P2 GpN major capsid | -1.50 | 4.90 |
| *ZMOp36x045* | bacteriophage P2 GpM family protein | -1.25 | 4.39 |
| *ZMOp36x046* | bacteriophage head completion protein GpL | -1.36 | 3.62 |
| *ZMOp36x053* | hypothetical protein | -1.92 | 4.08 |
| *ZMOp39x033* | hypothetical protein | -1.58 | 4.95 |
| *ZMOp39x036* | hypothetical protein | -1.43 | 7.44 |
| *ZMOp39x037* | hypothetical protein | -1.73 | 6.88 |
