# Supplementary material for: Investigation of the impact of a broad range of temperatures on the physiological and transcriptional profiles of Zymomonas mobilis ZM4 for high-temperature-tolerant recombinant strain development
Source: Biotechnol Biofuels. 2021 Jun 27;14:146. doi: 10.1186/s13068-021-02000-1 (PMC8237431; doi:10.1186/s13068-021-02000-1)
Supplement: Supplementary file 3 — Additional file 3: Table S3. List of eleven up-regulated genes of ZM4 cultured at 24 °C and 45 °C compared with 30 °C. Ratio (24/30) is the log2-based expression difference between ZM4 cultured at 24 °C and 30 °C. Ratio (45/30) is the log2-based expression difference between ZM4 cultured at 45 °C and 30 °C. P-value is − log10(P-value). [file 13068_2021_2000_MOESM3_ESM.docx]

**Table S3.** List of eleven upregulated genes of ZM4 cultured at 24℃ and 45℃ compared with 30℃. Ratio (24/30) is the log_2_-based expression difference between ZM4 cultured at 24℃ and 30℃. Ratio (45/30) is the log_2_-based expression difference between ZM4 cultured at 45℃ and 30℃. P-value is -log_10_(p-value).

| **Gene ID** | **Product** | **Ratio (24/30)** | **P-value of (24/30)** | **Ratio (45/30)** | **P-value of (45/30)** |
| --- | --- | --- | --- | --- | --- |
| ZMO0122 | Uncharacterized protein | 1.16 | 5.6 | 2.86 | 9.99 |
| ZMO0286 | DUF541 domain-containing protein | 1.23 | 6.17 | 2.56 | 9.78 |
| ZMO0693 | OsmC family protein | 1.62 | 8.66 | 1.57 | 8.5 |
| ZMO0740 | General stress protein CsbD | 1.89 | 5.74 | 3.06 | 8.03 |
| ZMO1113 | FAD-dependent pyridine nucleotide-disulfide oxidoreductase | 1.07 | 6.34 | 1.62 | 8.34 |
| ZMO1237 | Lactate dehydrogenase | 1.6 | 6.6 | 2.82 | 9.39 |
| ZMO1522 | TonB-dependent receptor | 1.3 | 4.89 | 1.01 | 3.88 |
| ZMO1533 | Hypothetical protein | 1.1 | 7.01 | 3.01 | 12.07 |
| ZMO1754 | Succinate-semialdehyde dehydrogenase SSADH | 1.27 | 5.75 | 3.95 | 11.33 |
| ZMO1776 | Aminopeptidase N | 1.14 | 6.9 | 1.97 | 9.64 |
| ZMO1940 | Hypothetical protein | 1.87 | 3.32 | 4.87 | 7.45 |
