# Supplementary material for: Investigation of the impact of a broad range of temperatures on the physiological and transcriptional profiles of Zymomonas mobilis ZM4 for high-temperature-tolerant recombinant strain development
Source: Biotechnol Biofuels. 2021 Jun 27;14:146. doi: 10.1186/s13068-021-02000-1 (PMC8237431; doi:10.1186/s13068-021-02000-1)
Supplement: Supplementary file 4 — Additional file 4: Table S4. List of primers used in this work. The underlined sequence indicates the homology arm for overlapping PCR or plasmid assembly by T5 exonuclease. [file 13068_2021_2000_MOESM4_ESM.docx]

**Table S4.** List of primers used in this work. The underlined sequence indicates the homology arm for overlapping PCR or plasmid assembly by T5 exonuclease.

| **Primer Name** | **Description** | **Sequence (5’ to 3’)** |
| --- | --- | --- |
| spe-ptet-F | Amplify *aadA_tetR_Ptet* | aattctcatgtttgacagcttatc |
| spe-ptet-R | Amplify *aadA_tetR_Ptet* | gggagatcctttctcctctttag |
| pZM36_005US-F | Amplify *pZM36_005US* | cccccgtaaaaatagccgttct |
| pZM36_005US-R | Amplify *pZM36_005US* | gagaattggatcccgctgacagacaccccgataagataagc |
| pZM36_005DS-F | Amplify *pZM36_005DS* | gaggagaaagggaattcatgaataatccggcatggatggatg |
| pZM36_005DS-R | Amplify *pZM36_005DS* | gtttccggcacagaccaattaacc |
| pZM32_014US-F | Amplify *pZM32_014US* | tttaagaaggagatatacatggaagtttttgcgctaatataccagc |
| pZM32_014US-R | Amplify *pZM32_014US* | gagaattggatcccgcaatatatgttttttattgttgactctttgcgataaaaaatc |
| pZM32_014DS-F | Amplify *pZM32_014DS* | gagaaaggctgcagatggctaataaagagcctcaagcag |
| pZM32_014DS-R | Amplify *pZM32_014DS* | ccttactcgagtttggatccgatcattgacgatctggttaatcag |
| pZM32_028US-F | Amplify *pZM32_028US* | gccgtccttcagtccgtattttg |
| pZM32_028US-R | Amplify *pZM32_028US* | catgagaattctgcagttctgaaatacggtaaaagcctgccg |
| pZM32_028DS-F | Amplify *pZM32_028DS* | gagaaaggggatccatgtccgatgattgcagcgg |
| pZM32_028DS-R | Amplify *pZM32_028DS* | caacccgttccgacttttcgatc |
| ZMO1465US-F | Amplify *ZMO1465US* | gaaggagatatacatatggccaaaactattccgatg |
| ZMO1465US-R | Amplify *ZMO1465US* | tcaaacatgagaatttcatcctgaaacggacataaaatc |
| ZMO1465DS-F | Amplify *ZMO1465DS* | gaggagaaaggatctcccatgtcgaataaacgagaccg |
| ZMO1465DS-R | Amplify *ZMO1465DS* | ctcgagtttggatccgacgcaggcttcacgaaag |
| ZMO1483US-F | Amplify *ZMO1483US* | tttaagaaggagatatacatgacatgctcactgcccaaaacg |
| ZMO1483US-R | Amplify *ZMO1483US* | catgagaattctgcagttctagaataggctgtattgcgcctttc |
| ZMO1483DS-F | Amplify *ZMO1483DS* | gagaaaggggatccatgttctctcttagggcatcgacttattttttattc |
| ZMO1483DS-R | Amplify *ZMO1483DS* | ccttactcgagtttggatcccgcttcattattgacacaatgtgcc |
| ZMO1628US-F | Amplify *ZMO1628US* | tttaagaaggagatatacatcgccgtccgtttttaacagtatc |
| ZMO1628US-R | Amplify *ZMO1628US* | gagaattggatcccgcactgattaaaattagaggggcttaaaatgc |
| ZMO1628DS-F | Amplify *ZMO1628DS* | gaggagaaagggaattcatgacggggggatcgaacag |
| ZMO1628DS-R | Amplify *ZMO1628DS* | ccttactcgagtttggatccccctgacttttctttgttggcc |
| ZMO2037US-F | Amplify *ZMO2037US* | ggtggcatcaccatccgc |
| ZMO2037US-R | Amplify *ZMO2037US* | gagaattggatcccggttaaggggcgaaaaaatccagtattgg |
| ZMO2037DS-F | Amplify *ZMO2037DS* | gaggagaaagggaattcatgatcctcgctctgctcaacc |
| ZMO2037DS-R | Amplify *ZMO2037DS* | gtcggccacctcagcag |
| ZMO0015US-F | Amplify *ZMO0015US* | cttcatcataaggtgcgccctcg |
| ZMO0015US-R | Amplify *ZMO0015US* | ctgtcaaacatgagaattctgatgcctgccctcgtgg |
| ZMO0015DS-F | Amplify *ZMO0015DS* | ggagaaaggctgcagatgtcatccataccggtcatagaattatcatcg |
| ZMO0015DS-R | Amplify *ZMO0015DS* | ccgtgatagggagaggcgataacg |
| ZMO0234US-F | Amplify *ZMO0234US* | gaaggagatatacatcgcttggactcaatcagactctg |
| ZMO0234US-R | Amplify *ZMO0234US* | ctgtcaaacatgagaatttttggcagacctcctgatttttttcgg |
| ZMO0234DS-F | Amplify *ZMO0234DS* | gagaaaggatctcccgtgcgctacgcctatgcc |
| ZMO0234DS-R | Amplify *ZMO0234DS* | ctcgagtttggatccgattaccgatcgccaacaccca |
| ZMO0236US-F | Amplify *ZMO0236US* | gaaggagatatacatctgtgcatctaccggctgc |
| ZMO0236US-R | Amplify *ZMO0236US* | ctgtcaaacatgagaattcaatcaacctgaaatgaaaacaaagagga |
| ZMO0236DS-F | Amplify *ZMO0236DS* | gagaaaggatctcccatggctgaccaagactgcaatg |
| ZMO0236DS-R | Amplify *ZMO0236DS* | ctcgagtttggatccacatcacattcactccagtccg |
| ZMO1335US-F | Amplify *ZMO1335US* | gaaggagatatacatcgcagatatcgggcagacttg |
| ZMO1335US-R | Amplify *ZMO1335US* | ctgtcaaacatgagaattaaggccactattttccagaccatgg |
| ZMO1335DS-F | Amplify *ZMO1335DS* | gagaaaggatctcccatgcctaaaattcttgttctttattattccacttatgg |
| ZMO1335DS-R | Amplify *ZMO1335DS* | ctcgagtttggatcccttattgggctgtcagctttttgg |
| ZMO1636US-F | Amplify *ZMO1636US* | gaaggagatatacatgtgccgatattgaactgcacttcg |
| ZMO1636US-R | Amplify *ZMO1636US* | ctgtcaaacatgagaatttatgcctccaaatgcaaaatacagcag |
| ZMO1636DS-F | Amplify *ZMO1636DS* | gagaaaggatctcccgtggtcgagattgttgttcttgtggta |
| ZMO1636DS-R | Amplify *ZMO1636DS* | ctcgagtttggatcccttgtttgatactacggctgaatgtcc |
| pZM36_005seq-R | verify ZM4_005 | gcagatcagcagaacgacaaacc |
| pZM36_005seq-F | verify ZM4_005 | gtcatcaatctgtctaacagtacctccag |
| pZM32_014seq-F | verify ZM4_014 | cgaaagcagaggttagatcggtg |
| pZM32_014seq-R | verify ZM4_014 | gctgcgccggttctgatc |
| pZM32_028seq-F | verify ZM4_028 | ctcgctttcccctgtgaatagc |
| pZM32_028seq-R | verify ZM4_028 | ccaatctgatcctgaccaatccacc |
| ZMO1465seq-F | verify ZM4_1465 | gccttgttgttggtgcgc |
| ZMO1465seq-R | verify ZM4_1465 | gaagccagcgcccttgc |
| ZMO1483seq-F | verify ZM4_1483 | cagcaagtgatgaggcgg |
| ZMO1483seq-R | verify ZM4_1483 | ccactgcgaaaccaaagctg |
| ZMO1628seq-F | verify ZM4_1628 | gaataggattgcaggcgcg |
| ZMO1628seq-R | verify ZM4_1628 | gaagcctgtccttgccaattcc |
| ZMO2037seq-F | verify ZM4_2037 | ccgttctatggctgatattgtagcgg |
| ZMO2037seq-R | verify ZM4_2037 | ccactgcgaaaccaaagctgac |
| ZMO0234seq-F | verify ZM4_0234 | gcatagcggacacgatacca |
| ZMO0234seq-R | verify ZM4_0234 | tgccgtcagaccattagctc |
| ZMO0236seq-F | verify ZM4_0236 | tgacaacggctggctgtaat |
| ZMO0236seq-R | verify ZM4_0236 | tcgaccgctcttttagcgtc |
| ZMO1335seq-F | verify ZM4_1335 | ttacggctgttgacatggct |
| ZMO1335seq-R | verify ZM4_1335 | tctccgaattccttgccctg |
| ZMO1636seq-F | verify ZM4_1636 | tcaaagccagcagaaggtgg |
| ZMO1636seq-R | verify ZM4_1636 | ctcgacatggaaaacgacgc |
| clpB-F | Amplify *clpB* | aagaggagaaaggatctcccatgaattttgagaaattaacagatcgagcaaagg |
| clpB-R（SacI, BioBrick） | Amplify *clpB* | ctgcagcggccgctactagtgagctccaacgctaatattctgcccttgttatcc |
| clpB-R | Amplify *clpB* | aaatcaaaggagccaaaacgagcaacgctaatattctgcccttg |
| groSL-F | Amplify *groESL* operon | cgttttggctcctttgatttcatgatttattttc |
| groSL-R(SpeI) | Amplify *groESL* operon | gactagtttagaaatccataccacccatgccg |
| 15A(RA,groSL)-R | Amplify pEZ_groESL | aaatcaaaggagccaaaacgctctagaagcggccgcgaattc |
| ZMO0994-F | Amplify *ZMO0994* | aagaggagaaaggatctcccatgtccagcaaccgactgactaaac |
| ZMO0994-R | Amplify *ZMO0994* | ctgcagcggccgctactagtttacttgcccttggacttgttaccac |
| cspL-F | Amplify *cspL* | aagaggagaaaggatctcccatggaacatggtacggttaaatggtttaattc |
| cspL-R | Amplify *cspL* | ctgcagcggccgctactagtttaatcttctttctgaacattggccgc |
