# Supplementary material for: Investigation of the impact of a broad range of temperatures on the physiological and transcriptional profiles of Zymomonas mobilis ZM4 for high-temperature-tolerant recombinant strain development
Source: Biotechnol Biofuels. 2021 Jun 27;14:146. doi: 10.1186/s13068-021-02000-1 (PMC8237431; doi:10.1186/s13068-021-02000-1)
Supplement: Supplementary file 5 — Additional file 5: Table S5. Sequences of pEZ15A and pUC57 vectors, EGFP and cspL genes, as well as promoters and terminators used in this study. [file 13068_2021_2000_MOESM5_ESM.docx]

**Table S5.** Sequences of pEZ15A and pUC57 vectors, *EGFP* and *cspL* genes, as well as promoters and terminators used in this study.

**>pEZ15A vector (5’ to 3’)**

gcgctagcggagtgtatactggcttactatgttggcactgatgagggtgtcagtgaagtgcttcatgtggcaggagaaaaaaggctgcaccggtgcgtcagcagaatatgtgatacaggatatattccgcttcctcgctcactgactcgctacgctcggtcgttcgactgcggcgagcggaaatggcttacgaacggggcggagatttcctggaagatgccaggaagatacttaacagggaagtgagagggccgcggcaaagccgtttttccataggctccgcccccctgacaagcatcacgaaatctgacgctcaaatcagtggtggcgaaacccgacaggactataaagataccaggcgtttccccctggcggctccctcgtgcgctctcctgttcctgcctttcggtttaccggtgtcattccgctgttatggccgcgtttgtctcattccacgcctgacactcagttccgggtaggcagttcgctccaagctggactgtatgcacgaaccccccgttcagtccgaccgctgcgccttatccggtaactatcgtcttgagtccaacccggaaagacatgcaaaagcaccactggcagcagccactggtaattgatttagaggagttagtcttgaagtcatgcgccggttaaggctaaactgaaaggacaagttttggtgactgcgctcctccaagccagttacctcggttcaaagagttggtagctcagagaaccttcgaaaaaccgccctgcaaggcggttttttcgttttcagagcaagagattacgcgcagaccaaaacgatctcaagaagatcatcttattaatcagataaaatatttcttgatttcagtgcaatttatctcttcaaatgtagcacctgaagtcagccccatacgatataagttgtaaattctcatgtttgacagcttatcatcgatggagcacaggatgacgcctaacaattcattcaagccgacaccgcttcgcggcgcggcttaattcaggagttaaacatcatgagggaagcggtgatcgccgaagtatcgactcaactatcagaggtagttggcgtcatcgagcgccatctcgaaccgacgttgctggccgtacatttgtacggctccgcagtggatggcggcctgaagccacacagtgatattgatttgctggttacggtgactgtaaggcttgatgaaacaacgcggcgagctttgatcaacgaccttttggaaacttcggcttcccctggagagagcgagattctccgcgctgtagaagtcaccattgttgtgcacgacgacatcattccgtggcgttatccagctaagcgcgaactgcaatttggagaatggcagcgcaatgacattcttgcaggtatcttcgagccagccacgatcgacattgatctggctatcttgctgacaaaagcaagagaacatagcgttgccttggtaggtccagcggcggaggaactctttgatccggttcctgaacaggatctatttgaggcgctaaatgaaaccttaacgctatggaactcgccgcccgactgggctggcgatgagcgaaatgtagtgcttacgttgtcccgcatttggtacagcgcagtaaccggcaaaatcgcgccgaaggatgtcgctgccgactgggcaatggagcgcctgccggcccagtatcagcccgtcatacttgaagctaggcaggcttatcttggacaagaagatcgcttggcctcgcgcgcagatcagttggaagaatttgttcactacgtgaaaggcgagatcaccaaggtagtcggcaaataatgtctaacaattcgttcaagccgacgccgcttcgcggcgcggcttaactcaagcgttagagagctggggaagactatgcgcgatctgttgaaggtggttctaagcctcgtacttgcgatggcatcggggcaggcacttgctgacctgccaagcaattccgtagtgagtactgaatttattctgattcgtcttgcttttggagcgtctttttgcgttctataactgttgtgaaagctacgcggtcgccattgaaaacgaaattaggattaataaaataccatccttggcgaacatgctttgcaatgattttagctttttctaattcggctagacctcttgcaaaggtagcttgagatagtgccagttttttttcttgtgcgttaagaaagtcctctaaaacgaatttgtctaaagggacgaggtctttgctgatgcctttgtcttgaagtatccaaaccagaacgctgaaagcttttattccagcggctcctagttcaaaagttagcgcgatattggtgctaaataattttacaaattcttcactatcaacacgtctgtaagtcgtcacatgagtgccttgcatctcaccagtggcttgattgaccagaatgttatcatctcgtcctaatcgagataactgaaccctctgacttttaactggcacaaccataccttcgatgaaaggattctcgtcatatctgattggctgctttctcaattttgtcgccatatttgataaacctttaatcaaaaaaaccacattttttgattatacctattcatcgaatgaggcaaggtctatcaattttacccctttttttgatagacggtttaatcaatattgatagaccccttcacagattctgaaaatcgacttccctattttagggatattttcacgattccctttcttagttcttcctagtggggaaattcgttgaatcctgcctcggaaaaaccatgagaaagctgttggttatatacacgggcaaagccaccctatttttagctactggggaaagagataaggcaggtcaccagctcaccgt

**>pUC57 vector (5’ to 3’)**

ggatccaaactcgagtaaggatctccaggcatcaaataaaacgaaaggctcagtcgaaagactgggcctttcgttttatgatatcggatcccgggcccgtcgactgcagaggcctgcatgcaagcttggcgtaatcatggtcatagctgtttcctgtgtgaaattgttatccgctcacaattccacacaacatacgagccggaagcataaagtgtaaagcctggggtgcctaatgagtgagctaactcacattaattgcgttgcgctcactgcccgctttccagtcgggaaacctgtcgtgccagctgcattaatgaatcggccaacgcgcggggagaggcggtttgcgtattgggcgctcttccgcttcctcgctcactgactcgctgcgctcggtcgttcggctgcggcgagcggtatcagctcactcaaaggcggtaatacggttatccacagaatcaggggataacgcaggaaagaacatgtgagcaaaaggccagcaaaaggccaggaaccgtaaaaaggccgcgttgctggcgtttttccataggctccgcccccctgacgagcatcacaaaaatcgacgctcaagtcagaggtggcgaaacccgacaggactataaagataccaggcgtttccccctggaagctccctcgtgcgctctcctgttccgaccctgccgcttaccggatacctgtccgcctttctcccttcgggaagcgtggcgctttctcatagctcacgctgtaggtatctcagttcggtgtaggtcgttcgctccaagctgggctgtgtgcacgaaccccccgttcagcccgaccgctgcgccttatccggtaactatcgtcttgagtccaacccggtaagacacgacttatcgccactggcagcagccactggtaacaggattagcagagcgaggtatgtaggcggtgctacagagttcttgaagtggtggcctaactacggctacactagaagaacagtatttggtatctgcgctctgctgaagccagttaccttcggaaaaagagttggtagctcttgatccggcaaacaaaccaccgctggtagcggtggtttttttgtttgcaagcagcagattacgcgcagaaaaaaaggatctcaagaagatcctttgatcttttctacggggtctgacgctcagtggaacgaaaactcacgttaagggattttggtcatgagattatcaaaaaggatcttcacctagatccttttaaattaaaaatgaagttttaaatcaatctaaagtatatatgagtaaacttggtctgacagttaccaatgcttaatcagtgaggcacctatctcagcgatctgtctatttcgttcatccatagttgcctgactccccgtcgtgtagataactacgatacgggagggcttaccatctggccccagtgctgcaatgataccgcgagacccacgctcaccggctccagatttatcagcaataaaccagccagccggaagggccgagcgcagaagtggtcctgcaactttatccgcctccatccagtctattaattgttgccgggaagctagagtaagtagttcgccagttaatagtttgcgcaacgttgttgccattgctacaggcatcgtggtgtcacgctcgtcgtttggtatggcttcattcagctccggttcccaacgatcaaggcgagttacatgatcccccatgttgtgcaaaaaagcggttagctccttcggtcctccgatcgttgtcagaagtaagttggccgcagtgttatcactcatggttatggcagcactgcataattctcttactgtcatgccatccgtaagatgcttttctgtgactggtgagtactcaaccaagtcattctgagaatagtgtatgcggcgaccgagttgctcttgcccggcgtcaatacgggataataccgcgccacatagcagaactttaaaagtgctcatcattggaaaacgttcttcggggcgaaaactctcaaggatcttaccgctgttgagatccagttcgatgtaacccactcgtgcacccaactgatcttcagcatcttttactttcaccagcgtttctgggtgagcaaaaacaggaaggcaaaatgccgcaaaaaagggaataagggcgacacggaaatgttgaatactcatactcttcctttttcaatattattgaagcatttatcagggttattgtctcatgagcggatacatatttgaatgtatttagaaaaataaacaaataggggttccgcgcacatttccccgaaaagtgccacctgacgtctaagaaaccattattatcatgacattaacctataaaaataggcgtatcacgaggccctttcgtctcgcgcgtttcggtgatgacggtgaaaacctctgacacatgcagctcccggagacggtcacagcttgtctgtaagcggatgccgggagcagacaagcccgtcagggcgcgtcagcgggtgttggcgggtgtcggggctggcttaactatgcggcatcagagcagattgtactgagagtgcaccatatgcggtgtgaaataccgcacagatgcgtaaggagaaaataccgcatcaggcgccattcgccattcaggctgcgcaactgttgggaagggcgatcggtgcgggcctcttcgctattacgccagctggcgaaagggggatgtgctgcaaggcgattaagttgggtaacgccagggttttcccagtcacgacgttgtaaaacgacggccagtgaattcgagctcggtacctcgcgaatgcatctagatatctttacactttatgcttccggctcgtataatgtgtggaattgtgagcggataacaatttcagaattcaaaagatcttttaagaaggagatatacat

**>*EGFP* (5’ to 3’) (Codon optimized for ZM4)**

Atggtgagcaagggcgaggagctgttcaccggggtggtgcccatcctggtcgagctggacggcgacgtaaacggccacaagttcagcgtgcgcggcgagggcgagggcgatgccaccaacggcaagctgaccctgaagttcatctgcaccaccggcaagctgcccgtgccctggcccaccctcgtgaccaccctgacctacggcgtgcagtgcttcagccgctaccccgaccacatgaagcagcacgacttcttcaagtccgccatgcccgaaggctacgtccaggagcgcaccatcagcttcaaggacgacggcacctacaagacccgcgccgaggtgaagttcgagggcgacaccctggtgaaccgcatcgagctgaagggcatcgacttcaaggaggacggcaacatcctggggcacaagctggagtacaacttcaacagccacaacgtctatatcaccgccgacaagcagaagaacggcatcaaggccaacttcaagatccgccacaacgtggaggacggcagcgtgcagctcgccgaccactaccagcagaacacccccatcggcgacggccccgtgctgctgcccgacaaccactacctgagcacccagtccgtgctgagcaaagaccccaacgagaagcgcgatcacatggtcctgctggagttcgtgaccgccgccgggatcactctcggcatggacgagctgtacaagtaa

**>*cspL* (5’ to 3’) (Codon optimized for ZM4)**

ATGGAACATGGTACGGTTAAATGGTTTAATTCTGAAAAAGGCTATGGTTTTATCGAACGGGAAGGTGGTGATGATGTTTTTGTTCATTTTAGCGCGATTCAGGGCGAAGGTTATAAAACGTTGGAAGAAGGCCAGAAAGTTTCTTTTGATGTTGAAGAAGGTTCTCGTGGTCCGCAGGCGGCCAATGTTCAGAAAGAAGATtaa

**>lacUV5 promoter (5’ to 3’)**

tttacactttatgcttccggctcgtataatgtgtggaattgtgagcggataacaatttcagaattcaaaagatcttttaagaaggagatatacat

>**t*etR_Ptet* (TetR Repressor and *Ptet* promoter, 5’ to 3’)**

Ttaagacccactttcacatttaagttgtttttctaatccgcatatgatcaattcaaggccgaataagaaggctggctctgcaccttggtgatcaaataattcgatagcttgtcgtaataatggcggcatactatcagtagtaggtgtttccctttcttctttagcgacttgatgctcttgatcttccaatacgcaacctaaagtaaaatgccccacagcgctgagtgcatataatgcattctctagtgaaaaaccttgttggcataaaaaggctaattgattttcgagagtttcatactgtttttctgtaggccgtgtacctaaatgtacttttgctccatcgcgatgacttagtaaagcacatctaaaacttttagcgttattacgtaaaaaatcttgccagctttccccttctaaagggcaaaagtgagtatggtgcctatctaacatctcaatggctaaggcgtcgagcaaagcccgcttattttttacatgccaatacaatgtaggctgctctacacctagcttctgggcgagtttacgggttgttaaaccttcgattccgacctcattaagcagctctaatgcgctgttaatcactttacttttatctaatctggacatcattaattcctaatttttgttgacactctatcgttgatagagttattttaccactccctatcagtgatagagaaaagtattcaaatgatct

>***rrnB* T1 terminator (5’ to 3’)**

Caaataaaacgaaaggctcagtcgaaagactgggcctttcgtttta

>***T7Te* terminator (5’ to 3’)**

ggctcaccttcgggtgggcctttctgcg
